# Homoeolog-specific activation of genes for heat acclimation in the allopolyploid grass *Brachypodium hybridum*

**DOI:** 10.1093/gigascience/giy020

**Published:** 2018-03-08

**Authors:** Kotaro Takahagi, Komaki Inoue, Minami Shimizu, Yukiko Uehara-Yamaguchi, Yoshihiko Onda, Keiichi Mochida

**Affiliations:** 1 Graduate School of Nanobioscience, Yokohama City University, 22-2 Seto, Kanazawa-ku, Yokohama, Kanagawa 236-0027, Japan; 2 Kihara Institute for Biological Research, Yokohama City University, 641-12 Maioka-cho, Totsuka-ku, Yokohama, Kanagawa 244–0813, Japan; 3 Cellulose Production Research Team, Biomass Engineering Research Division, RIKEN Center for Sustainable Resource Science, 1-7-22 Suehiro-cho, Tsurumi-ku, Yokohama, Kanagawa 230-0045, Japan; 4 Institute of Plant Science and Resources, Okayama University, 2-20-1 Chuo, Kurashiki, Okayama 710-0046, Japan

**Keywords:** abiotic stress response, allopolyploidy, *Brachypodium hybridum*, heat acclimation, homoeolog, hybrid species, transcriptome

## Abstract

**Background:**

Allopolyploid plants often show wider environmental tolerances than their ancestors; this is expected to be due to the merger of multiple distinct genomes with a fixed heterozygosity. The complex homoeologous gene expression could have been evolutionarily advantageous for the adaptation of allopolyploid plants. Despite multiple previous studies reporting homoeolog-specific gene expression in allopolyploid species, there are no clear examples of homoeolog-specific function in acclimation to a long-term stress condition.

**Results:**

We found that the allopolyploid grass *Brachypodium hybridum* and its ancestor *Brachypodium stacei* show long-term heat stress tolerance, unlike its other ancestor, *Brachypodium distachyon*. To understand the physiological traits of *B. hybridum*, we compared the transcriptome of the 3 *Brachypodium* species grown under normal and heat stress conditions. We found that the expression patterns of approximately 26% and approximately 38% of the homoeolog groups in *B. hybridum* changed toward nonadditive expression and nonancestral expression, respectively, under normal condition. Moreover, we found that *B. distachyon* showed similar expression patterns between normal and heat stress conditions, whereas *B. hybridum* and *B. stacei* significantly altered their transcriptome in response to heat after 3 days of stress exposure, and homoeologs that were inherited from *B. stacei* may have contributed to the transcriptional stress response to heat in *B. hybridum*. After 15 days of heat exposure, *B. hybridum* and *B. stacei* maintained transcriptional states similar to those under normal conditions. These results suggest that an earlier response to heat that was specific to homoeologs originating from *B. stacei* contributed to cellular homeostasis under long-term heat stress in *B. hybridum*.

**Conclusions:**

Our results provide insights into different regulatory events of the homoeo-transcriptome that are associated with stress acclimation in allopolyploid plants.

## Background

Polyploidy is a common phenomenon in eukaryotes, especially in plants [1–4], and is recognized as a fundamental mechanism in plant evolution and diversification [5, 6]. It has been suggested that all angiosperms have experienced 1 or more polyploidization events during their evolutionary history [7–10]. Recent evolutionary genomic studies have suggested that genome duplication events occurred widely in plants at the Cretaceous–Paleogene boundary, which is a major extinction event in the earth's history. Evolutionary views represent a hypothesis that plants with duplicated genomes might have a better chance for survival under global adverse conditions [11, 12].

Interspecific hybridization and subsequent genome duplication led to evolutional changes in hybrid species that represented fixed heterozygosity. Allopolyploid plants generally show better growth vigor and stress tolerance than their ancestors [13–15]. For example, relative to their ancestors, allopolyploid *Arabidopsis* (*Arabidopsis suecica*) shows more vigorous growth, and allopolyploid *Spartina* (*Spartina anglica*) shows better tolerance to reducing conditions and sulfite-rich sediments [16–18]. Both natural and synthetic wheat (*Triticum aestivum*) have higher fitness under salt stress than their diploid and tetraploid ancestors [19, 20]. It has been suggested that allopolyploidization may have contributed to the adaptation to a wide range of environmental conditions [21, 22].

The expression levels of homoeologs in allopolyploid species often show nonadditive expressions that are deviated from parental additivity compared to their ancestors [23]. The nonadditive expression has been widely observed in various allopolyploid species [24–26] and can be explained by at least 3 possible scenarios: total gene expression of a homoeolog group in an allopolyploid species is similar to that of 1 of its parental species (expression level dominance [ELD]), total gene expression is lower or higher than in both parents (transgressive expression), and uneven contribution of homoeologs to gene expression (homoeolog expression bias) [23, 27]. Through comparative gene expression analysis of total expression levels of homoeolog groups in an allopolyploid species and “mid-parental values” as the average expression levels of genes in its parents, parental additivity and nonadditivity of homoeologs can be examined. By discrimination of homoeologous gene expression, homoeolog expression bias can be identified across tissues, developmental stages, and environmental conditions [28–30].

The contribution of expression plasticity of duplicated genes observed in diverse allopolyploid species to adaptive and ecological fitness has been debated. Comparative transcriptome analysis of the natural allopolyploid *Coffea arabica* and its ancestral species (*Coffea canephora* and *Coffea eugenioides*) revealed evidence of a genomic ELD that depends on growth temperature [31]. Comparative transcriptome analysis of hexaploid *Triticum aestivum* (AABBDD) and its ancestors (*Triticum turgidum* [AABB] and *Aegilops tauschii* [DD]) demonstrated differential enrichment of overrepresented gene functions between the ELD genes of both parents, which suggests they may have differentially contributed to particular biological functions in hexaploid wheat [32]. Comparative gene expression analysis of a synthetic tetraploid wheat and its parental species showed transgressive expression of genes that are involved in particular biological functions such as transport, modification, and uroporphyrinogen decarboxylase activity, suggesting that transgressive gene expression may have rapidly occurred following allopolyploidization [33]. Studies on the allotetraploid *Arabidopsis kamchatica* and its ancestral species demonstrated homoeolog expression bias in genes related to adaptation to cold and heavy metal environments, which were inherited from the respective ancestors that are tolerant against cold and heavy metal stresses, respectively [34, 35].

*Brachypodium hybridum* (2n = 30) is a natural allopolyploid that is derived from a cross between *Brachypodium distachyon* (2n = 10) and *Brachypodium stacei* (2n = 20) that occurred approximately 1 million years ago (MYA; Additional file 1, Fig. S1) [36–43]. Although these species inhabit a circum-Mediterranean region, their environmental niches are clearly different. *Brachypodium distachyon* grows in higher-altitude, cooler, and wetter areas, whereas *B. stacei* is found in lower-altitude, warmer, and drier areas. The hybrid species *B. hybridum* grows in areas that are overlapping as well as specific to its ancestors; this growth pattern suggests that its speciation is associated with particular environmental conditions and adaptations to diverse environmental conditions [37]. The broader growth area of *B. hybridum* compared to its ancestors led us to the hypothesis that its duplicated genes may have contributed to its adaptive and ecological fitness through gene expression changes following allotetraploidization. Specifically, in the *B. hybridum* transcriptome, each of the homoeologs may show particular expression patterns in response to abiotic stimuli, which are associated with the ancestral growth habitats and gene expression. Therefore, this trio of species has been recently proposed as a model for grass speciation via adaptation and polyploidization [39, 40, 43].

In the current study, we conducted a comparative global transcriptome analysis of *B. hybridum* and its ancestors. The analysis revealed nonadditive transcriptome changes in the leaf and root tissues of *B. hybridum*. Additionally, we performed a homoeolog-specific transcriptome analysis by discriminating RNA-sequencing (RNA-seq) reads of each homoeolog of *B. hybridum* and determined the nonancestral gene expression patterns. Finally, we assessed homoeolog-specific transcriptome changes in response to heat stress in *B. hybridum.* Here, we discuss the differential regulation of the homoeo-transcriptome that is associated with heat stress tolerance in *B. hybridum* inherited from *B. stacei*.

## Data Description

### Plant materials

Three *Brachypodium* species were used in this study: the allotetraploid *B. hybridum* Bd14-1 and the diploid ancestors *B. distachyon* Bd21 and *B. stacei* ABR114. The accessions of these species were provided by the National Plant Germplasm System of the US Department of Agriculture–Agricultural Research Service (David F. Garvin; Plant Science Research Unit, University of Minnesota, USA) and Pilar Catalán (Department of Agriculture and Environment Science, High Polytechnic School of Huesca, University of Zaragoza, Spain). Dry seeds were incubated on wet filter paper in a Petri dish at 4°C in the dark for 6–7 days to synchronize germination. The germinated seeds were grown in a growth chamber at 25°C under a 16-h day photoperiod (60 μmol·m^–2^·s^–1^) for 4 days. The plants were transplanted to pots filled with autoclaved Pro-Mix® BX Mycorrhizae™ (Premier Tech, Quebec, Canada). The potted plants were grown in a growth chamber at 22°C (normal condition) or 32°C (heat stress condition) under a 20-h day photoperiod (100 μmol·m^–2^·s^–1^) and watered with 5000-fold diluted Professional Hyponex 10-30-20 (Hyponex Japan, Osaka, Japan) every 3 or 4 days.

### Whole genome sequence data

Genomic DNA from the leaf tissues of *B. hybridum* and *B. stacei* were extracted using the DNeasy Plant Mini Kit (Qiagen, Tokyo, Japan). Libraries for single-end DNA sequencing were obtained using Ion Xpress™ Plus Fragment library kits (Life Technologies Japan, Tokyo, Japan); semiconductor chips that were used for sequencing were prepared using the Ion OneTouch 2 System (Life Technologies Japan) and Ion P1™ Chip v2 (Life Technologies Japan). The sequencing analyses were performed using an Ion Proton sequencer (Life Technologies Japan). The genome sequence data of *B. hybridum* and *B. stacei* were archived at DNA Data Bank of Japan (DDBJ) under the accession number DRA005717 (Additional file 2, Table S1).

### RNA-seq data

Shoots and roots from each species grown in a growth chamber for 4 days after synchronized germination were sampled to elucidate the global homoeolog expression patterns in *B. hybridum*. Shoots and most young leaf blades from each species grown in a growth chamber under different temperature conditions (22°, normal condition, and 32°C, heat stress condition) for 3 and 15 days, respectively, after being transplanted were sampled to elucidate the homoeolog-specific transcriptional response to heat stress in *B. hybridum*. Total RNA was extracted from each sample using the RNeasy plant mini kit (Qiagen). Poly(A) RNAs were purified using the NEBNext^®^ Poly(A) mRNA Magnetic Isolation Module (New England Biolabs, MA, USA). The libraries for single-end strand-specific RNA-seq were obtained using the Ion Total RNA-seq kit v.2 (Life Technologies Japan). Size-selected libraries were purified using the Agencourt AMPure XP (Beckman Coulter, CA, USA). Semiconductor chips that were used for sequencing were prepared using the Ion PI™ Hi-Q™ OT2 200 kit (Life Technologies Japan), Ion PI™ Hi-Q™ Sequencing 200 kit (Life Technologies Japan), and Ion P1™ Chip v3 (Life Technologies Japan). The sequencing analyses were performed using an Ion Proton sequencer (Life Technologies Japan) with 3 biological replicates. The RNA-seq data were archived at DDBJ under the accession number DRA005699 (Additional file 2, Table S1).

## Results

### *B. hybridum* and *B. stacei* show significant tolerance to long-term heat stress

**Figure 1:**
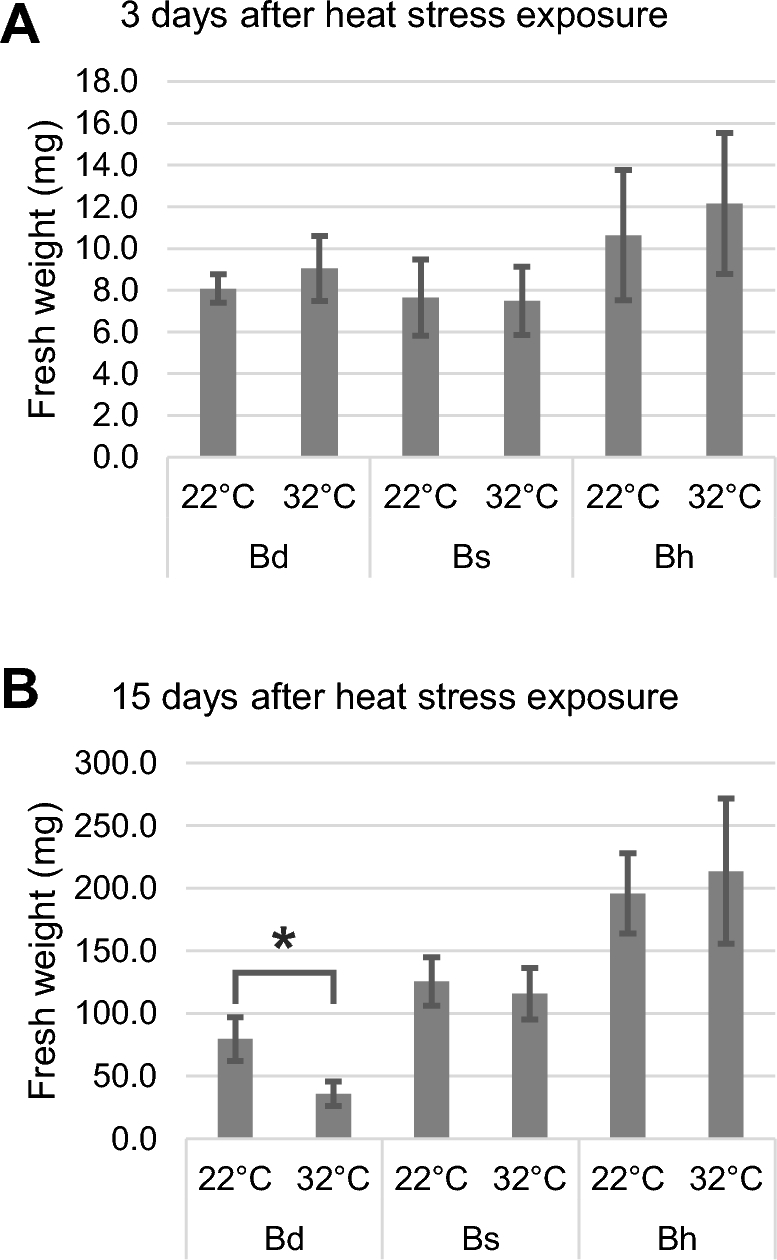
Effect of heat stress on the growth of the 3 *Brachypodium* species. Fresh weight of *B. distachyon*, *B. stacei*, and *B. hybridum* grown under normal (22°C) and heat stress (32°C) conditions at 3 (A) and 15 (B) days after stress exposure. Data are the mean value ± standard deviation for 12 individuals. Statistical differences are indicated by an asterisk (*P* < 0.01, *t* test). Bd, *B. distachyon*; Bs, *B. stacei*; Bh, *B. hybridum*.

Unlike *B. distachyon*, *B. hybridum* and *B. stacei* showed significant tolerance to long-term heat stress. The allopolyploid *B. hybridum* and its ancestor *B. stacei* grow in warmer regions, in contrast to the other ancestor *B. distachyon*, which suggests that *B. hybridum* and *B. stacei*, but not *B. distachyon*, might have adapted to high-temperature conditions. To test this hypothesis, we compared the plant biomasses of *B. hybridum*, *B. stacei*, and *B. distachyon* grown under normal (22°C) and heat stress (32°C) conditions at 2 time points (after 3 and 15 days of heat stress exposure). After 3 days of exposure to heat stress, the 3 species showed no differences in growth (Fig. 1A). Conversely, after 15 days of heat exposure, *B. distachyon* showed a significant decrease in fresh weight (*P* < 0.01, *t* test), whereas *B. hybridum* and *B. stacei* maintained their growth (Fig. 1B). This result indicates that *B. hybridum* and *B. stacei* are thermotolerant species and that *B. hybridum* might have inherited this trait from *B. stacei* via allopolyploidization.

### A virtual *B. stacei* genome generated from the comparative analysis of homoeologous genomes

**Figure 2:**
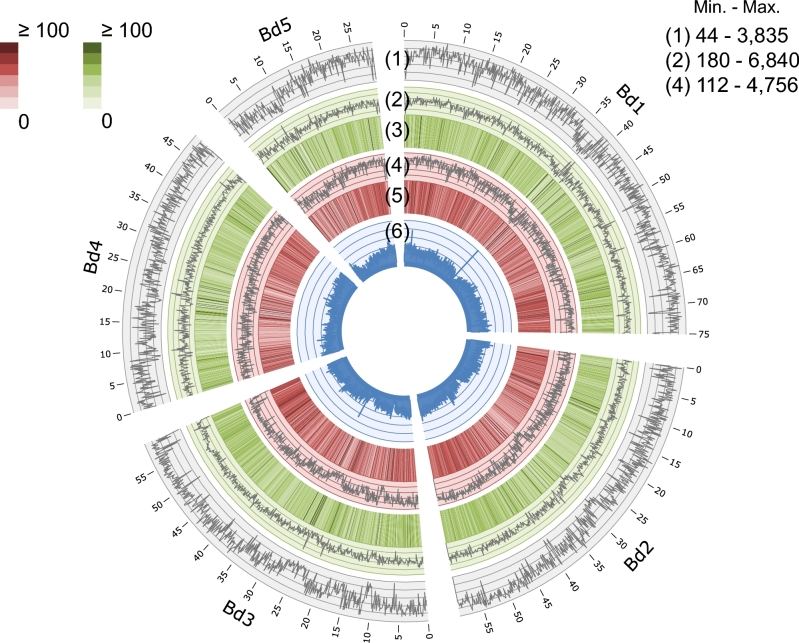
Summary of genome sequence data. Mapping results and polymorphic features of the genomic reads from *B. hybridum* and *B. stacei* superimposed to the reference genome sequence of Bd21 (100-kb sliding windows). 1) Number of homoeologous SNPs. 2) Number of heterogenic SNPs between *B. distachyon* and *B. hybridum*. 3) Average number of *B. hybridum* reads mapped to the reference genome. 4) Number of homogenic SNPs between *B. distachyon* and *B. stacei*. 5) Average number of *B. stacei* reads mapped to the reference genome. 6) Density distribution of the Bd21 annotated genes.

By comparing the genomes of *B. hybridum*, *B. stacei*, and *B. distachyon*, we obtained a comprehensive map of the homoeologous single nucleotide polymorphisms (SNPs) in these *Brachypodium* species. This map enabled the distinction of transcripts expressed from each of the homoeologs in *B. hybridum*. To determine polymorphisms between the homoeologous genomes in *B. hybridum*, we sequenced the genomes of *B. stacei* ABR114 and *B. hybridum* Bd14-1 and mapped the reads to the reference genome sequence of *B. distachyon* Bd21. We found that 85% and 91% of the genomic reads of *B. stacei* and *B. hybridum* mapped to the reference genome and covered 89% and 98% of the genic region of the reference genome, respectively, which suggests high similarity among the homoeologous genomes (Fig. 2 and Additional file 2, Table S2). As for genomic polymorphisms, 11 948 285 SNPs were identified between *B. distachyon* and *B. stacei* and 10 216 010 SNPs were identified between *B. distachyon* and *B. hybridum* (Additional file 2, Table S3). We selected 5 720 539 SNPs to discriminate the homoeologs that were homogenic to the *B. stacei* reads from those that were heterogenic to the *B. hybridum* reads (Fig. 2; Additional file 1, Fig. S2; and Additional file 3). By replacing the nucleotides of the homoeologous SNPs in the *B. distachyon* genome with those in *B. stacei*, we generated virtual homoeolog sequences of *B. stacei* that corresponded to *B. distachyon* counterparts (Additional file 1, Fig. S3).

### Genome-wide polymorphism between *B. distachyon* and *B. stacei* genomes

Through a comprehensive identification of homoeologous SNPs between *B. distachyon* and *B. stacei* genomes, we assessed the distribution of the SNPs within various genomic regions to gain insights into the genetic diversity of the 2 genomes. We found that 22.8% of homoeologous SNPs are distributed within the intergenic regions and that the remaining 77.2% of the SNPs were identified in the genic regions (Additional file 1, Fig. S4A). Specifically, 45.4% and 17.6% of SNPs are distributed in intronic and exonic regions, respectively, and 12.5% and 1.6% of SNPs are distributed in untranslated regions and splice sites, respectively. We assessed the types of polymorphism found in genic regions and found that 26 986 genes (79% of the annotated genes in the Bd21 genome) contain nonsynonymous variations. Of these, 4558 genes (13% of the annotated genes in the Bd21 genome) contain several variations in the *B. stacei* genome; 683, 2980, and 895 genes show start codon loss, stop codon gain, and stop codon loss, respectively (Additional file 1, Fig. S4B and Additional file 2, Table S4). Based on the synonymous substitution rate (*Ks*) between the homoeologs, we estimated a divergence time of approximately 6.4 MYA between *B. distachyon* and *B. stacei*, which is in accordance with the divergence time of *B. stacei* of 5.8–16.4 MYA previously estimated by Catalan et al. [38]. Moreover, we computed the nonsynonymous substitution rate (*Ka*) and *Ka*/*Ks* ratio and identified 1714 homoeologs whose *Ka*/*Ks* values were greater than 1, supporting their accelerated evolution. Interestingly, we found that genes involved in DNA mismatch repair with gene ontology (GO) terms such as proliferating cell nuclear antigen complex and DNA polymerase processivity factor activity (GO:00 43626 and GO:00 30337, respectively, *P* < 0.01) are significantly enriched in those genes, which suggested that DNA damage tolerance may reflect selection and adaptive divergence between *B. distachyon* and *B. stacei*.

### Nonadditive gene expression in *B. hybridum*

**Figure 3:**
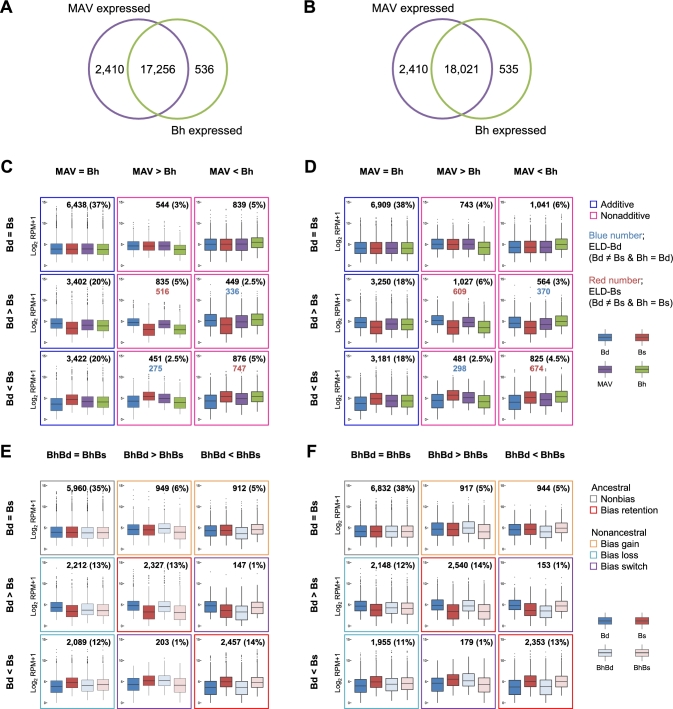
Global gene expression patterns in *B. hybridum* compared with its ancestors. A and B) Venn diagrams of genes expressed in the MAV (left) and homoeolog groups expressed in *B. hybridum* (right) in leaf (A) and root (B) tissues. C and D) Additive and nonadditive gene expression patterns in leaf (C) and root (D) tissues of *B. hybridum*. Genes (homoeolog groups) expressed in both the MAV and *B. hybridum* (17 256 genes [homoeolog groups]) were analyzed. E and F) Ancestral and nonancestral gene expression patterns in leaf (E) and root (F) tissues in *B. hybridum*. Genes (homoeolog groups) expressed in both the MAV and *B. hybridum* (18 021 genes [homoeolog groups]) were analyzed. “ = ” indicates that the gene expression level is not significantly different between the compared groups. “>” and “<” indicate that the gene expression level is significantly different between the compared groups. The significance threshold is set at FDR ≤0.001. Bd, *B. distachyon*; Bs, *B. stacei*; MAV, mid-ancestral value; BhBd, Bd-homoeologs in *B. hybridum*; BhBs, Bs-homoeologs in *B. hybridum*; Bh, *B. hybridum*.

To identify gene expression changes in *B. hybridum* as a result of the tetraploidization, we examined expression levels of its homoeolog groups in comparison to mid-ancestral values (MAVs) of those estimated from each of the counterpart homoeologs in its ancestors, *B. distachyon* and *B. stacei*, and identified the homoeolog groups that showed nonadditive expression in the leaf and root transcriptomes of *B. hybridum*. To this end, we performed RNA-seq–based transcriptome analysis of leaf and root mRNA samples of *B. distachyon*, *B. stacei*, and *B. hybridum* and quantified gene expression of the homoeologs and their counterparts in the ancestors by mapping the RNA-seq reads to the Bd21 genome (*B. distachyon* reads), the virtual *B. stacei* genome (*B. stacei* reads), or both genomes (*B. hybridum* reads) (Additional file 1, Fig. S5A). Through comparisons of the MAV estimated from the ancestors and expression level of the homoeolog groups in *B. hybridum*, we found that 17 256 and 18 021 (50% and 53% of the annotated genes in the Bd21 genome) of genes and homoeolog groups are expressed (supported by MAV ≥1 and reads per million mapped reads [RPM] ≥1 in *B. hybridum*) in the root and leaf transcriptomes, respectively (Fig. 3A and B). Then, we classified these expressed homoeolog groups according to additive or nonadditive expression and found that 3994 and 4681 homoeolog groups (23% and 26% of the analyzed homoeolog groups in leaf and root tissues, respectively) were nonadditively expressed in the *B. hybridum* leaf and root transcriptomes, respectively (the remaining 13 262 and 13 340 homoeolog groups in leaf and root tissues, respectively, were additively expressed; Fig. 3C and D). Moreover, we assessed ELD in the nonadditively expressed homoeolog groups. We found that 611 and 668 homoeolog groups in leaf and root tissues, respectively (3.5% and 3.7% of the analyzed homoeolog groups), in *B. hybridum* showed ELD, with expression levels similar to those in *B. distachyon* (ELD-Bd) (Fig. 3C and D). On the other hand, 1263 and 1283 homoeolog groups in leaf and root tissues, respectively (7.3% and 7.1% of analyzed homoeolog groups), in *B. hybridum* showed ELD of *B. stacei* (ELD-Bs) (Fig. 3C and D). These findings indicate that the number of homoeolog groups of ELD-Bs is nearly twice that of ELD-Bd, suggesting that gene expression patterns of *B. stacei* are preferentially inherited to the *B. hybridum* transcriptome in leaf and root tissues. We also identified 627 and 850 homoeolog groups in *B. hybridum* leaf and root tissues, respectively (3.6% and 4.7% of the analyzed homoeolog groups), that showed transgressive expression, i.e., higher expression than in both ancestors. Various GO terms, such as primary metabolic processes, primary cellular processes, stress response, protein transport, localization, and translation, were enriched in the set of homoeolog groups that showed transgressive expression (Additional file 2, Tables S5 and S6), suggesting that the overproduction of transcripts may affect various biological functions through the tetraploidization.

### Homoeolog expression bias in *B. hybridum*

To identify homoeolog expression bias in *B. hybridum*, we sorted the *B. hybridum* RNA-seq reads to the 2 homoeologous genomes of *B. distachyon* (Bd-subgenome) and *B. stacei* (Bs-subgenome), based on their sequence identities after alignments with both genomes, and quantified the expression levels of each of the Bd- and Bs-homoeologs in the *B. hybridum* transcriptome. Through homoeolog-specific RNA-seq read sorting, we discriminated 75–79% of the RNA-seq reads between the Bd-subgenome origin reads and Bs-subgenome origin reads (Additional file 1, Fig. S5 and S6). We found that 6512 and 6296 homoeolog groups show nonancestral expression patterns in the *B. hybridum* leaf and root transcriptomes, respectively (38% and 35% of the analyzed homoeolog groups; orange, light blue, and purple boxes in Fig. 3E and F), and the remaining 10 744 and 11 725 retained the ancestral expression patterns (gray and red boxes in Fig. 3E and F). In the homoeolog groups showing ancestral expression patterns, we found that similar proportions of the homoeolog groups retained the expression bias of each of the ancestral transcriptomes (27% of the analyzed homoeolog groups in leaf and root tissues; red boxes in Fig. 3E and F). In the homoeolog groups showing nonancestral expression patterns, we observed bias loss in most cases in the *B. hybridum* transcriptomes (25% and 23% of the analyzed homoeolog groups in leaf and root tissues, respectively; light blue boxes in Fig. 3E and F). These findings suggest that more than 30% of the homoeologous gene pairs in *B. hybridum* had changed their expression pattern toward nonancestral expression during speciation after the genome-scale gene duplication via allopolyploidization.

### Homoeolog-specific transcriptional response to heat stress in *B. hybridum*

**Figure 4:**
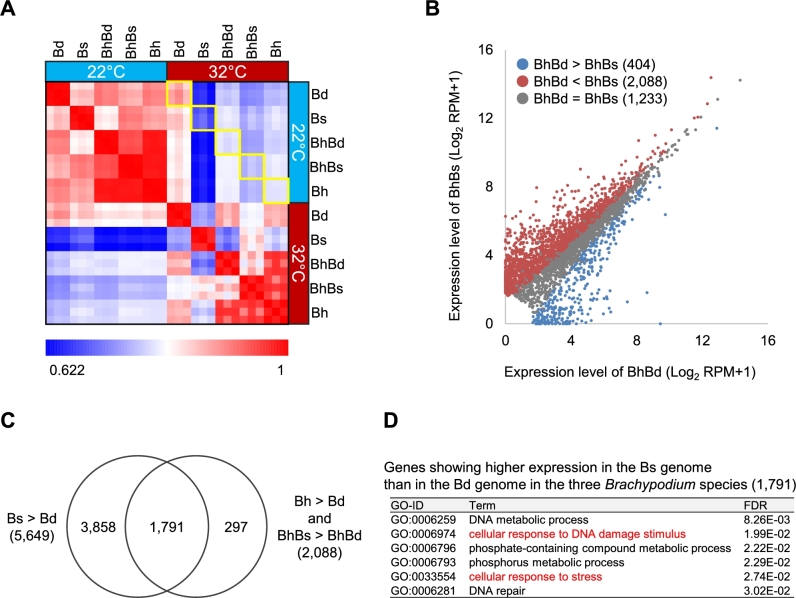
Heat stress response in *B. hybridum*. A) Heat map of Pearson correlation coefficients (PCCs) based on the gene expression profiles of each species at 3 days after heat stress exposure. PCC values between different conditions are shown in yellow squares. B) Gene expression profiles of the Bd- and Bs-homoeologs in homoeolog groups showing significantly higher expression in *B. hybridum* than in *B. distachyon* under heat stress conditions at 3 days after stress exposure. Each dot represents the average expression value of 3 biological replicates. Blue dots represent genes showing significantly higher expression in Bd-homoeologs than in Bs-homoeologs, and red dots represent genes showing significantly higher expression in Bs-homoeologs than in Bd-homoeologs (FDR ≤0.001). C) Genes showing significantly higher expression in the *B. stacei* genome than in the *B. distachyon* genome in the 3 *Brachypodium* species under heat stress conditions for 3 days. The left circle represents genes showing significantly higher expression in *B. stacei* than in *B. distachyon*. The right circle represents genes showing significantly higher expression in Bs-homoeologs than in Bd-homoeologs in homoeolog groups showing significantly higher expression in *B. hybridum* than in *B. distachyon*. D) Enriched GO terms in the biological process ontology of 1791 intersectional genes in the Venn diagram shown in (C). Bd, *B. distachyon*; Bs, *B. stacei*; BhBd, Bd-homoeologs in *B. hybridum*; BhBs, Bs-homoeologs in *B. hybridum*; Bh, *B. hybridum*.

*Brachypodium distachyon* transcriptomes were not significantly different between normal (22°C) and heat stress (32°C) conditions after 3 days of exposure to heat stress, whereas the *B. hybridum* transcriptome was noticeably altered under the heat stress as compared to the normal condition; this might reflect physiological traits inherited from *B. stacei*. When gene expression patterns of *B. distachyon*, *B. stacei*, and *B. hybridum*, as well as the Bd- and Bs-homoeologs of *B. hybridum* after 3 days of exposure to heat stress were compared, we found that the transcriptomes of *B. stacei* and *B. hybridum* showed lower correlations between normal and heat stress conditions than those of *B. distachyon* (Pearson correlation coefficient [PCC], 0.68–0.75, 0.87, and 0.92–0.95 in *B. stacei*, *B. hybridum*, and *B. distachyon*, respectively; Fig. 4A), suggesting that *B. stacei* and *B. hybridum* respond more strongly to heat stress than *B. distachyon*. We also found that after 3 days of stress exposure, 5649 genes and 3725 homoeolog groups showed significantly higher expression in *B. stacei* and *B. hybridum*, respectively, than in *B. distachyon* (false discovery rate [FDR], ≤0.001). We then dissected the expression patterns of the 3725 homoeolog groups from *B. hybridum* into Bd- and Bs-homoeologs and found that 2088 homoeolog groups (56% of the 3725 homoeolog groups) were preferentially expressed by the Bs-homoeologs (Fig. 4B). The distribution of fold-change expression values between Bd- and Bs-homoeologs in all expressed homoeolog groups in *B. hybridum* under heat stress for 3 days indicated that most of the homoeologs were evenly expressed by both subgenomes (Additional file 1, Fig. S7A), whereas the 3725 homoeolog groups showed abundant expression in the Bs-homoeologs (Additional file 1, Fig. S7B). Additionally, of the 2088 Bs-homoeologs, 1791 genes were shared with the 5649 genes that showed higher expression in *B. stacei* than in *B. distachyon*; these genes included those specifically involved in metabolic processes as well as cellular response to stress and damage stimulus (Fig. 4C and D). These results suggest that the functions of *B. hybridum* genes that were inherited from *B. stacei* may have contributed to the transcriptional stress response and associated metabolic changes in the *B. hybridum* transcriptome during the earlier response to heat stress.

### Early transcriptional responses of Bs-homoeologs contribute to the maintenance of cellular functions in *B. hybridum* under long-term heat stress

**Figure 5:**
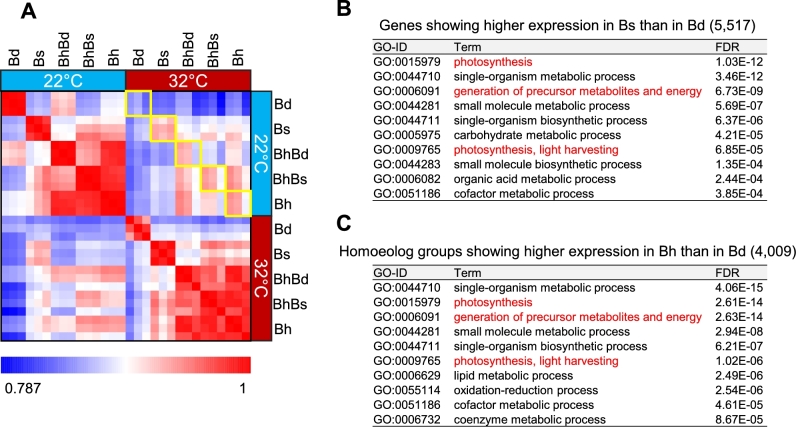
Maintenance of transcriptional states in *B. hybridum* and *B. stacei* under long-term heat stress. A) Heat map of Pearson's correlation coefficients (PCCs) based on the gene expression profiles of each species at 15 days after heat stress exposure. PCC values between different conditions are marked by yellow squares. B and C) Top 10 enriched GO terms in the biological process ontology of genes showing significantly higher expression in *B. stacei* than in *B. distachyon* (B) and homoeolog groups showing significantly higher expression in *B. hybridum* than in *B. distachyon* (C) at 15 days after heat stress exposure. All enriched GO terms for these genes and homoeolog groups are shown in Additional file 2, Tables S7 and S8. Bd, *B. distachyon*; Bs, *B. stacei*; BhBd, Bd-homoeologs in *B. hybridum*; BhBs, Bs-homoeologs in *B. hybridum*; Bh, *B. hybridum*.

After 15 days of exposure to heat stress, *B. hybridum* and *B. stacei* maintained their transcriptional states similar to those under normal condition, in contrast to the severely damaged cellular system of *B. distachyon*. When we compared the expression patterns between *B. distachyon*, *B. stacei*, and *B. hybridum* after 15 days of exposure to heat stress, we found that *B. stacei* and *B. hybridum* showed similar expression patterns between stress and normal conditions (PCC, 0.94–0.96 and 0.92–0.96, respectively), whereas *B. distachyon* showed remarkable changes in its transcriptome (PCC, 0.84–0.89), reflecting the severe decrease in its biomass (Fig. 1B and 5A). Specifically, genes involved in primary metabolism, such as photosynthesis and metabolite and energy generation, were significantly less represented in *B. distachyon* than in *B. stacei* and *B. hybridum* under long-term heat stress (Fig. 5B and C and Additional file 2, Tables S7 and S8), which likely indicates the physiological sensitivity of its cellular system to heat stress. The number of Bs-homoeologs that were expressed more abundantly than Bd-homoeologs was reduced in homoeolog groups that were highly expressed in *B. hybridum* than in *B. distachyon* at 15 days after exposure to heat stress compared to that found after 3 days of heat stress exposure (Fig. 4B and Additional file 1, Fig. S8), which suggests that Bs-homoeologs are significantly activated at the earlier phase of transcriptional response to heat stress. We also found significantly higher expression of Bs-homoeologs and genes in *B. stacei* encoding A2-type heat shock transcription factor (*HsfA2*) as well as putative *HsfA2*-targeted genes such as heat shock protein 101 (*Hsp101*) and ascorbate peroxidase 2-like (*APX2*-like; homologs of *Arabidopsis APX1* and rice *APX2*), which are known as key factors in response to heat [44–46] compared to expression of their counterparts in *B. distachyon* at 3 days after exposure to 32°C (Additional file 1, Fig. S9). To validate the homoeolog-specific gene expression in *B. hybridum* observed in our RNA-seq data, we quantified the polymorphic nucleotides in the cDNA amplicons of *B. hybridum* using a TaqMan SNP genotyping assay in combination with a digital polymerase chain reaction (PCR) system. The assay discriminates between 2 homoeo-alleles of a specific SNP that are labeled with 2 fluorescent dyes (FAM™ and VIC™ (Thermo Fisher Scientific, MA, USA)) and quantifies the expression of each allele by detecting the fluorescence. Specifically, we assessed the quantitative distribution of the decisive SNPs in the 2 homoeolog groups corresponding to the genes annotated in the Bd21 genome, Bradi1g16510 (*APX2*-like) and Bradi2g49660 (*Hsp101*). We found that the proportions of SNPs in the cDNA amplicons from the *B. hybridum* RNAs sampled after 3 days of exposure to 32°C were comparable to their expression bias observed in the RNA-seq analysis (Additional file 1, Fig. S10 and S11). These results suggest that the earlier response to heat stress that was specific to the Bs-homoeologs likely contributed to the maintenance of their cellular homeostasis under the long heat exposure.

## Discussion

### Evolutionary nonadditive gene expression in the hybrid grass *B. hybridum* likely underlies its enhanced invasive behavior

Our transcriptome analysis provides comprehensive evidence for nonadditive gene expression in the hybrid species *B. hybridum*, suggesting the global transcriptional changes in its leaves and roots evolved through allopolyploidization. The GO analysis of nonadditively expressed homoeolog groups in leaf and root tissues of *B. hybridum* relative to both ancestral species showed an overrepresentation of genes that were involved in the response to stimulus and abiotic stimulus (Additional file 2, Tables S9 and S10). Such functions were also enriched in the homoeolog groups in leaf tissues of *B. hybridum* that showed nonadditive expression under heat stress conditions (Additional file 2, Tables S11 and S12), which suggests that increased expression divergence of genes related to such functions enhanced this species’ ability to respond to environmental change and to adapt to ecological niches. Increased expression divergence of these classes of genes has also been reported in allotetraploid *Arabidopsis* and wheat [24–26]. Although such examples have been reported in few species, future progress in transcriptome datasets of hybrid species and their ancestors might enable the application of universal rules for determining transcriptional changes when new hybrid species are generated. Allopolyploid species have long been hypothesized to possess greater environmental adaptability to wider niches than their ancestors. Specifically, enhanced heterozygosity and genetic diversity that result from the hybridization of multiple diverged genomes have been thought to upgrade stress tolerances and contribute to the expansion of niches in hybrid species [47–49]. Although *B. hybridum* shows the largest niche overlap compared with its diploid ancestral species, it shows a niche breadth that is smaller than that of *B. distachyon* and slightly greater than that of *B. stacei* [37, 38]. However, *B. hybridum* also successfully colonized nonnative regions of the world [37], which suggests it has greater ecological tolerance than other diploids. With the nonadditive transcriptional changes in *B. hybridum*, the expanded diversity of gene expression might contribute to the colonization of nonnative areas while avoiding inbreeding depression and might boost its diversifying selection [50, 51].

### Homoeolog-specific gene expression underlies acclimation to heat stress

**Figure 6:**
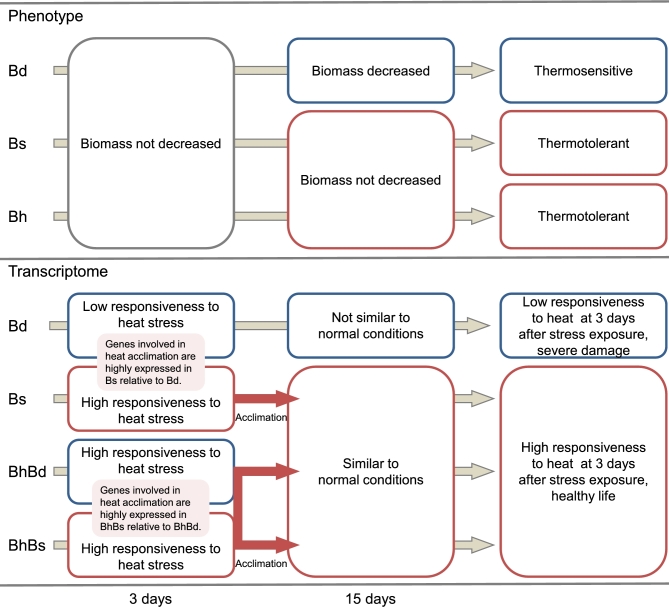
Model of phenotype and transcriptome of the 3 *Brachypodium* species under long-term heat stress. Bd, *B. distachyon*; Bs, *B. stacei*; BhBd, Bd-homoeologs in *B. hybridum*; BhBs, Bs-homoeologs in *B. hybridum*; Bh, *B. hybridum*.

Although no differences in visible traits were found between the 3 *Brachypodium* species after 3 days of exposure to heat stress, significant differences in physiological traits in the early stage of heat exposure were revealed from their transcriptomes. The comprehensive list of Bs-homoeolog and *B. stacei* genes with significantly higher expression than the *B. distachyon* genes after 3-day exposure to 32°C included genes involved in the regulation of acclimation to heat in plants, such as heat shock transcription factor, heat shock protein, and DNAJ [52–55] (1791 genes in Fig. 4C, Additional file 2, Table S13). Thus, the functions of *B. hybridum* in acclimating to heat are likely specifically inherited from *B. stacei* via allopolyploidization. Based on our transcriptome analysis using the *Brachypodium* trio, we hypothesize that the transcriptional response of Bs-homoeologs in *B. hybridum* and their counterparts in *B. stacei* during the early phase of heat exposure contributes to the maintenance of their physiological equilibrium under long-term heat exposure and might presumably be associated with their heat stress tolerance (Fig. 6). Previous evolutional and ecological studies suggested that *B. distachyon* could have adapted to different environments by diverging from *B. stacei* [37, 38]. The distribution areas of *B. distachyon* and *B. stacei* suggest that the 2 *Brachypodium* species have adapted to cooler and wetter areas and to warmer and drier areas, respectively, via diversification [37]. Therefore, the heat acclimation function in *B. distachyon* might have been lost during its adaptation process after branching from the common ancestor of these diploids. The heat-adaptive trait in the *B. stacei* genome could influence the survival of both individual plants and hybrid progeny under heat stress conditions.

## Methods

### Fresh weight measurement

The aboveground parts of plants of each species grown under normal and heat stress conditions for 3 or 15 days were used to measure fresh weight. Twelve individuals were used for the measurement. A *t* test was used for statistical comparisons between the plants grown under the different conditions. The significance threshold was set at *P* < 0.01.

### Coverage calculation and SNP calling

To obtain high-quality reads, the genome sequence reads were trimmed by cutting bases off from the start and end of reads if quality ≤20 and by removing the reads with final read length <50 using Trimmomatic v.0.32 (Trimmomatic, RRID:SCR_011848) [56] with the -thread 2 LEADING: 20 TRAILING: 20 MINLEN: 50 commands. The trimmed reads were mapped to the reference genome sequence of Bd21 downloaded from Phytozome [57] using TMAP (v.3.1.4; Life Technologies Japan, Tokyo, Japan) with mapall -n 4 -v -Y -u -o 2 stage1 map4 commands. The coverage of genome sequence reads on the reference genome was calculated by removing the nonmapped reads and merging multiple mapping data of the same species from the raw mapping data using SAMTools v.0.1.19 (SAMTools, RRID:SCR_002105) [58] with the view -F 4 and merge commands. The merged data were subjected to coverage calculation using the genomeCoverageBed in BEDTools v.2.20.1 (BEDTools, RRID:SCR_006646) [59] with the default setting. SNPs between the genome sequence reads and reference genome were called by removing the nonmapped reads and possible duplicate reads and merging the multiple mapping data of the same species from the raw mapping data using SAMTools with the view -F 4, rmdup, and merge commands. The merged data were subjected to SNP calling using VarScan pileup2snp (v.2.3.7) [60] with the default settings (minimum read depth to make a call is 8 and minimum supporting reads to call variants is 2), except –*P* value 0.01 commands.

### Homoeologous SNP identification and virtual *B. stacei* genome construction

Common homogenic SNPs between *B. distachyon* and *B. stacei* and heterogenic SNPs between *B. distachyon* and *B. hybridum* were identified as homoeologous SNPs (Additional file 3); these were used to identify homoeologous genomes in *B. hybridum*. The homoeolog-specific SNPs and their impact on the transcripts and deduced protein sequences were predicted using SnpEff (v.4.2) [61] with the gene structural annotation of Bd21 retrieved from Phytozome (Bdistachyon_314_v3.1.gene_exons.gff3.gz) [57] and -ud 0 command. A virtual *B. stacei* genome was constructed by replacing the nucleotides of the homoeologous SNPs in the *B. distachyon* genome with those in the *B. stacei* genome using an original Perl script (Additional file 4).

### *Ka* and *Ks* calculation and estimation of the speciation time of *B. distachyon* and *B. stacei*

The *Ka* and *Ks* values between genes of homoeolog groups were calculated with Ka_Ks Calculator (v.2.0) [62] using a modified version that implements the Yang-Nielsen algorithm (MYN) method [63]. The speciation time (*T*) based on the *Ks* value was estimated using the equation *T* = *Ks*/2*λ*, where *λ* = 6.1 × 10^−9^ [64, 65], based on the average *Ks* value of each homoeolog group.

### Read count and reads per million calculations

To obtain high-quality reads, the RNA-seq reads were trimmed by cutting bases off from the start and end of reads if quality ≤20 and by removing the reads with final read length <50 using Trimmomatic with -thread 4 LEADING: 20 TRAILING: 20 MINLEN: 50 commands. The trimmed reads were mapped to Bd21 and the virtual *B. stacei* genome using TMAP with mapall -n 4 -v -Y -u -o 2 stage1 map4 commands; *B. distachyon* reads were mapped to the Bd21 genome; *B. stacei* reads were mapped to the virtual *B. stacei* genome; and *B. hybridum* reads were mapped to both of these genomes. The expression levels of the homoeologs in *B. hybridum* were quantified by classifying the RNA-seq reads into the following 3 groups based on the number of mismatches between read and both genomes using original Perl script (Additional file 5): Bd-subgenome origin reads, Bs-genome origin reads, and unclassified reads (Additional file 1, Fig. S5B). The mapping data from the *B. distachyon* reads, *B. stacei* reads, Bd-subgenome origin reads, and Bs-genome origin reads were subjected to read count using featureCounts (v.1.4.6) [66] with the gene structural annotation of Bd21. For the MAV, which is the average of the expression values in both ancestors, the total read count data from the *B. distachyon* and *B. stacei* reads were used; for the entire expression value of the homoeolog group in *B. hybridum*, the total read count data for the Bd- and the Bs-subgenome origin reads were used (Additional file 1, Fig. S5A). The RPM values were calculated for all annotated genes based on the read count data. Genes, MAVs, homoeologs, and homoeolog groups with RPM ≥1 in all 3 biological replicates were defined as expressed.

### Validation of homoeolog-specific gene expression in *B. hybridum*

The ratios of expression between homoeologs were verified using a digital PCR system in combination with the TaqMan^®^ SNP genotyping assay method (Applied Biosystems, CA, USA) [67]. PCR primers were designed at conserved regions between homoeologs, and a targeted homoelogous SNP was included in the amplified region. Two dye-labeled (FAM and VIC) TaqMan probes were designed for quantifying homoeologous SNPs using the TaqMan MGB SNP kit (Applied Biosystems). cDNA was prepared from *B. hybridum* RNA sampled after 3-day exposure to heat stress (with 3 biological replicates) using SuperScript^®^ IV reverse transcriptase (Invitrogen, CA, USA) and was used for quantitative PCR. The thermocycling conditions were initiated at 96°C for 10 min, followed by 39 cycles of annealing and extension at 56°C for 2 min and denaturation at 98°C for 30 s, followed by a final extension at 56°C for 2 min. Homoeologous SNPs in the cDNA amplicons were detected with QuantStudio™ 3D (Life Technologies Japan). The PCR primers and TaqMan probes used in this assay are listed in Additional file 2, Table S14.

### Differentially expressed genes analysis

Differentially expressed genes between the 3 *Brachypodium* species, homoeolog groups, and growth conditions were identified using the DESeq2 package (v.1.10.1) [68] in R (v.3.2.4) with the Wald test based on the read count data. The FDR for each comparison was calculated by adjusting the *P* value using the Benjamini-Hochberg procedure. Genes with an FDR ≤0.001 were defined as differentially expressed.

### GO enrichment analysis

GO annotations of the *B. distachyon* genes were prepared using the method used in Koda et al. [69]. GO terms for the *B. distachyon* genes were used from the annotation information retrieved from Phytozome (Bdistachyon_314_v3.1.annotation_info.txt) [57]. Additional GO terms were associated with the *B. distachyon* genes based on GO terms of transcripts for *Arabidopsis thaliana* and rice in the “Best-hit-arabi-name” and “Best-hit-rice-name” row in the annotation file. The GO terms for genes annotated in *A. thaliana* and rice were used from the annotation information downloaded from Phytozome (Athaliana_167_TAIR10.annotation_info.txt [70], and Osativa_204_v7.0.annotation_info.txt [71]). To reduce bias, GO terms assigned to more than 3000 genes in *B. distachyon* were excluded. Enriched GO terms for selected genes were identified using Basic Local Alignment Search Tool 2GO (v.3.3.5) [72] with the Fisher exact test. All annotated genes in the Bd21 genome were used as a reference set. The enriched GO terms were summarized using the web-based tool REVIGO [73] if more than 10 enriched GO terms were found. GO annotations of the *B. distachyon* genes used in this study are provided in Additional file 6.

## Availability of supporting data

All sequencing data were archived at DDBJ under accession numbers DRA005717 and DRA005699 (BioProject accessions: PRJDB5654 and PRJDB5657). Supporting data are available in the *GigaScience* repository GigaDB [74] and via additional files. Additional files 1 and 2 provide Supplementary Figures and Supplementary Tables, respectively. Additional file 3 provides the homoeologous SNP dataset used in this study. Additional files 4 and 5 provide the original Perl script codes used in this study. Additional file 6 provides the GO annotations of the *B. distachyon* genes used in this study. The protocol for detection of allele frequencies in the cDNA sample is available via protocols.io [75].

## Additional files

Additional file 1 Figure S1: Phylogenetic relationships among the three *Brachypodium* species.

Additional file 1 Figure S2: Results of homoeologous SNP identification.

Additional file 1 Figure S3: Overview of genomic sequence data analysis.

Additional file 1 Figure S4: Location and effect of homoeologous SNPs.

Additional file 1 Figure S5: Overview of RNA-seq data analysis.

Additional file 1 Figure S6: Classification of the read origin of the *B. hybridum* RNA reads from leaf and root tissues.

Additional file 1 Figure S7: Log_2_ fold-change distribution of homoeolog expression in *B. hybridum* under heat stress conditions for 3 days.

Additional file 1 Figure S8: Gene expression profiles of the Bd- and Bs-homoeologs in homoeolog groups showing significantly higher expression in *B. hybridum* than in *B. distachyon* under heat stress conditions for 15 days.

Additional file 1 Figure S9: Gene expression profiles of the *Brachypodium HsfA2* and putative *HsfA2*-targeted genes at 3 days after heat stress exposure.

Additional file 1 Figure S10: Quantitative detection of fluorescence of homoeologs in *B. hybridum*.

Additional file 1 Figure S11: Expression ratios of Bd- and Bs-homoeologs based on RNA-seq analysis and TaqMan SNP Genotyping Assay.

Additional file 2 Table S1: Summary of the sequencing analysis and accession numbers.

Additional file 2 Table S2: Summary of the whole genome sequencing analysis and mapping results.

Additional file 2 Table S3: Results of SNP calling.

Additional file 2 Table S4: Numbers of variant effects in genes.

Additional file 2 Table S5: Enriched GO terms in the biological process ontology of homoeolog groups showing transgressive expression in *B. hybridum* leaf under normal condition.

Additional file 2 Table S6: Enriched GO terms in the biological process ontology of homoeolog groups showing transgressive expression in *B. hybridum* root under normal condition.

Additional file 2 Table S7: Enriched GO terms in the biological process ontology of genes showing significantly higher expression in *B. stacei* than in *B. distachyon* at 15 days after heat stress exposure.

Additional file 2 Table S8: Enriched GO terms in the biological process ontology of homoeolog groups showing significantly higher expression in *B. hybridum* than in *B. distachyon* at 15 days after heat stress exposure.

Additional file 2 Table S9: Enriched GO terms in the biological process ontology of homoeolog groups showing non-additive expression in *B. hybridum* leaf under normal condition.

Additional file 2 Table S10: Enriched GO terms in the biological process ontology of homoeolog groups showing non-additive expression in *B. hybridum* root under normal condition.

Additional file 2 Table S11: Enriched GO terms in the biological process ontology of homoeolog groups showing non-additive expression in *B. hybridum* leaf at 3 days after heat stress exposure.

Additional file 2 Table S12: Enriched GO terms in the biological process ontology of homoeolog groups showing non-additive expression in *B. hybridum* leaf at 15 days after heat stress exposure.

Additional file 2 Table S13: Genes showing higher expression in the *B. stacei* genome than in the *B. distachyon* genome in the three *Brachypodium* species at 3 days after heat stress exposure.

Additional file 2 Table S14: Amplicon primer and TaqMan probes used in this study.

Additional file 3: Homoeologous SNP dataset used in this study.

Additional file 4: Original Perl script code used to construct the virtual *B. stacei* genome by replacing the nucleotides of the homoeologous SNPs in the *B. distachyon* genome with those in the *B. stacei* genome.

Additional file 5: Original Perl script code used to classify the RNA-seq reads of *B. hybridum* into the Bd-subgenome origin reads, Bs-subgenome origin reads, and unclassified reads.

Additional file 6: GO annotations of the *B. distachyon* genes used in this study.

## Abbreviations

ELD: expression level dominance; FDR: false discovery rate; GO: gene ontology; MAV: mid-ancestral value;MYA: million years ago; PCC: Pearson correlation coefficient; PCR: polymerase chain reaction; RNA-seq: RNA-sequencing; RPM: reads per million mapped reads; SNP: single nucleotide polymorphism.

## Supplementary Material

GIGA-D-17-00181_Original_Submission.pdf

GIGA-D-17-00181_Revision_1.pdf

GIGA-D-17-00181_Revision_2.pdf

Response_to_Reviewer_Comments_Original_Submission.pdf

Response_to_Reviewer_Comments_Revision_1.pdf

Reviewer_1_Report_(Original_Submission) -- Julie Ferreira de Carvalho 07 Sep 2017 Reviewed

Reviewer_1_Report_(Revision_1) -- Julie Ferreira de Carvalho 18 Jan 2018 Reviewed

Reviewer_2_Report_(Original_Submission) -- Kranthi Mandadi 14 Sep 2017 Reviewed

Reviewer_2_Report_(Revision_1) -- Kranthi Mandadi 31 Jan 2018 Reviewed

Additional Files
