# Supplementary material for: Homoeolog-specific activation of genes for heat acclimation in the allopolyploid grass Brachypodium hybridum
Source: Gigascience. 2018 Mar 8;7(4):giy020. doi: 10.1093/gigascience/giy020 (PMC5915950; doi:10.1093/gigascience/giy020)
Supplement: GIGA-D-17-00181_Original_Submission.pdf [file giy020_giga-d-17-00181_original_submission.pdf]

GigaScience

Homoeolog-specific activation for heat acclimation in the allopolyploid grass <i>Brachypodium hybridum</i>

--Manuscript Draft--

**Manuscript Number:**	GIGA-D-17-00181
**Full Title:**	Homoeolog-specific activation for heat acclimation in the allopolyploid grass <i>Brachypodium hybridum</i>
**Article Type:**	Research
**Funding Information:**	Japan Society for the Promotion of Science (26712003) — Dr. Keiichi Mochida<br>Japan Science and Technology Agency (J2013403) — Dr. Keiichi Mochida
**Abstract:**	Background: Allopolyploid plants often show wider environmental tolerances than their ancestors; this difference would be expected due to the merger of multiple distinct genomes with a fixed heterozygosity. The complex homoeologous gene expression could have been evolutionarily advantageous for the adaptation of allopolyploid plants. Despite multiple previous studies reporting homoeolog-specific gene expression in allopolyploid species, there are no clear examples of homoeolog-specific function in acclimation to a long-term stress condition.<br>Results: We found that the allopolyploid grass <i>Brachypodium hybridum</i> and its ancestor <i>Brachypodium stacei</i> show long-term heat stress tolerance, unlike its another ancestor <i>Brachypodium distachyon</i>. To understand the physiological traits of <i>B. hybridum</i>, we compared the transcriptome of the three <i>Brachypodium</i> species grown under normal and heat stress conditions. We found that ~26% and ~38% of the homoeologous gene pairs in <i>B. hybridum</i> changed its expression pattern towards non-additive expression and non-parental expression, respectively, under normal condition. Moreover, we found that <i>B. distachyon</i> was transcriptionally insensitive, whereas <i>B. hybridum</i> and <i>B. stacei</i> were sensitive to heat at 3 days after stress exposure, and homoeologs that were inherited from <i>B. stacei</i> may have contributed to the transcriptional stress response to heat in <i>B. hybridum</i>. At 15 days after heat exposure, <i>B. hybridum</i> and <i>B. stacei</i> maintained transcriptional states similar to those under normal conditions. These results suggested an earlier response to heat that was specific to homoeologs originating from <i>B. stacei</i> contributed to cellular homeostasis under long-term heat stress in <i>B. hybridum</i>.<br>Conclusions: Our results provide insights into different regulatory events of the homoeo-transcriptome that are associated with stress acclimation in allopolyploid plants.
**Corresponding Author:**	Keiichi Mochida<br><br>JAPAN
**Corresponding Author Secondary Information:**	
**Corresponding Author's Institution:**	
**Corresponding Author's Secondary Institution:**	
**First Author:**	Kotaro Takahagi
**First Author Secondary Information:**	
**Order of Authors:**	Kotaro Takahagi<br>Komaki Inoue<br>Minami Shimizu<br>Yukiko Uehara-Yamaguchi

Powered by Editorial Manager® and ProduXion Manager® from Aries Systems Corporation

	Yoshihiko Onda
	Keiichi Mochida
**Order of Authors Secondary Information:**	
**Opposed Reviewers:**	John Vogel<br>DOE Joint Genome Institute<br><br>Competitor in genome sequencing of <i>Brachypodium hybridum</i> and <i>Brachypodium stacei</i>.
	Todd Mockler<br>Donald Danforth Plant Science Center<br><br>Competitor in genome sequencing of <i>Brachypodium hybridum</i> and <i>Brachypodium stacei</i>.
	Henry Priest<br>Donald Danforth Plant Science Center<br><br>Competitor in genome sequencing of <i>Brachypodium hybridum</i> and <i>Brachypodium stacei</i>.
**Additional Information:**	
**Question**	**Response**
Are you submitting this manuscript to a special series or article collection?	No
**Experimental design and statistics**<br><br>Full details of the experimental design and statistical methods used should be given in the Methods section, as detailed in our Minimum Standards Reporting Checklist. Information essential to interpreting the data presented should be made available in the figure legends.<br><br>Have you included all the information requested in your manuscript?	Yes
**Resources**<br><br>A description of all resources used, including antibodies, cell lines, animals and software tools, with enough information to allow them to be uniquely identified, should be included in the Methods section. Authors are strongly encouraged to cite Research Resource Identifiers (RRIDs) for antibodies, model organisms and tools, where possible.<br><br>Have you included the information requested as detailed in our Minimum Standards Reporting Checklist?	Yes

Powered by Editorial Manager® and ProduXion Manager® from Aries Systems Corporation

**Availability of data and materials**<br><br>All datasets and code on which the conclusions of the paper rely must be either included in your submission or deposited in publicly available repositories (where available and ethically appropriate), referencing such data using a unique identifier in the references and in the "Availability of Data and Materials" section of your manuscript.<br><br>Have you have met the above requirement as detailed in our Minimum Standards Reporting Checklist?	Yes

Powered by Editorial Manager® and ProduXion Manager® from Aries Systems Corporation

Manuscript Click here to download Manuscript Manuscript.docx

Homoeolog-specific activation for heat acclimation in the allopolyploid grass *Brachypodium hybridum*

Kotaro Takahagi[1,2,3], Komaki Inoue[3], Minami Shimizu[2,3], Yukiko Uehara-Yamaguchi[3], Yoshihiko Onda[2,3] and Keiichi Mochida[1,2,3,4]

[1]Graduate School of Nanobioscience, Yokohama City University, 22-2 Seto, Kanazawa-ku, Yokohama, Kanagawa 236-0027, Japan.

[2]Kihara Institute for Biological Research, Yokohama City University, 641-12 Maioka-cho, Totsuka-ku, Yokohama, Kanagawa 244-0813, Japan.

[3]Cellulose Production Research Team, Biomass Engineering Research Division, RIKEN Center for Sustainable Resource Science, 1-7-22 Suehiro-cho, Tsurumi-ku, Yokohama, Kanagawa 230-0045, Japan.

[4]Institute of Plant Science and Resources, Okayama University, 2-20-1 Chuo, Kurashiki, Okayama 710-0046, Japan.

E-mail addresses

Kotaro Takahagi n155267a@yokohama-cu.ac.jp

Komaki Inoue komaki.inoue@riken.jp

Minami Shimizu minami.shimizu@riken.jp

Yukiko Uehara-Yamaguchi yukiko.uehara@riken.jp

Yoshihiko Onda yoshihiko.onda@riken.jp

Keiichi Mochida keiichi.mochida@riken.jp

Corresponding author

Keiichi Mochida, Cellulose Production Research Team, Biomass Engineering Research Division, RIKEN Center for Sustainable Resource Science, 1-7-22 Suehiro-cho, Tsurumi-ku, Yokohama, Kanagawa 230-0045, Japan. Tel: +81-45-503-9111, E-mail: keiichi.mochida@riken.jp

Abstract

Background: Allopolyploid plants often show wider environmental tolerances than their ancestors; this difference would be expected due to the merger of multiple distinct genomes with a fixed heterozygosity. The complex homoeologous gene expression could have been evolutionarily advantageous for the adaptation of allopolyploid plants. Despite multiple previous studies reporting homoeolog-specific gene expression in allopolyploid species, there are no clear examples of homoeolog-specific function in acclimation to a long-term stress condition.

Results: We found that the allopolyploid grass *Brachypodium hybridum* and its ancestor *Brachypodium stacei* show long-term heat stress tolerance, unlike its another ancestor *Brachypodium distachyon*. To understand the physiological traits of *B. hybridum*, we compared the transcriptome of the three *Brachypodium* species grown under normal and heat stress conditions. We found that ~26% and ~38% of the homoeologous gene pairs in *B. hybridum* changed its expression pattern towards non-additive expression and non-parental expression, respectively, under normal condition. Moreover, we found that *B. distachyon* was transcriptionally insensitive, whereas *B. hybridum* and *B. stacei* were sensitive to heat at 3 days after stress exposure, and homoeologs that were inherited from *B. stacei* may have contributed to the transcriptional stress response to heat in *B. hybridum*. At 15 days after heat exposure, *B. hybridum* and *B. stacei* maintained transcriptional states similar to those under normal conditions. These results suggested an earlier response to heat that was specific to homoeologs originating from *B. stacei* contributed to cellular homeostasis under

long-term heat stress in *B. hybridum*.

Conclusions: Our results provide insights into different regulatory events of the homoeo-transcriptome that are associated with stress acclimation in allopolyploid plants.

Keywords

Abiotic stress response; Allopolyploidy; *Brachypodium hybridum*; Heat acclimation; Homoeolog; Hybrid species; Transcriptome

Background

Polyploidy is a common phenomenon in eukaryotes, especially in plants [1-4], and is recognized as a fundamental mechanism in plant evolution and diversification [5, 6]. It has been suggested that all angiosperms have experienced one or more polyploidization events during their evolutionary history [7-10]. Recent evolutionary genomic studies have suggested that genome duplication events occurred widely in plants at the Cretaceous–Paleogene boundary, which is a major extinction event in the earth's history. Evolutionary views represent a hypothesis that plants with duplicated genomes might have a better chance for survival under global adverse conditions [11, 12].

Interspecific hybridization and subsequent genomic duplication led to evolutional changes in hybrid species that represented fixed heterozygosity. Allopolyploid plants generally show better growth

vigour and stress tolerance than their ancestors [13-15]. For example, relative to their ancestors, allopolyploid *Arabidopsis* (*Arabidopsis suecica*) shows more vigorous growth, and allopolyploid *Spartina* (*Spartina anglica*) shows better tolerance to reducing conditions and sulfite-rich sediments [16-18]. Moreover, both natural and synthesized wheat (*Triticum aestivum*) have higher fitness under salt stress than their diploid and tetraploid ancestors [19, 20]. It has been suggested that allopolyploidization may have contributed to the adaptation to a wide range of environmental conditions [21, 22].

Allopolyploid species often show differential gene expression patterns between homoeologs [23-29]. Multiple studies have shown that homoeologous gene pairs are differentially regulated depending on developmental stages, organs, and environmental conditions [30]. Some of the differential transcriptional regulation of homoeologs may be associated with stress tolerances inherited from their ancestral species [31, 32]. The complex gene expression patterns caused by duplicated genomes could have been evolutionarily advantageous for the adaptation of allopolyploid plants.

Brachypodium hybridum (*B. hybridum*, 2n=30) is a natural allopolyploid that is derived from a cross between *Brachypodium distachyon* (*B. distachyon*, 2n=10) and *Brachypodium stacei* (*B. stacei*, 2n=20) that occurred approximately 1 million years ago (MYA; Additional file 1: Figure S1) [33-40]. Although these species inhabit a circum-Mediterranean region, their environmental niches are clearly different. *B. distachyon* grows in higher, cooler, and wetter areas, whereas *B. stacei* is found in lower, warmer, and drier areas. The hybrid species *B. hybridum* grows in areas that are overlapping as well as specific to its ancestors; this growth

pattern suggests that speciation is associated with particular environmental conditions and adaptations to diverse environmental conditions [34]. Recently, this trio of species has been proposed as a model for grass speciation via adaptation and polyploidization [36, 37, 40]

Herein, we determined the global properties of the transcriptome of *B. hybridum* and compared these properties to those of its ancestors. Our comparative transcriptome analysis between the three *Brachypodium* species shows non-additive transcriptome changes that are found in the leaf and root tissues of *B. hybridum*. Moreover, we performed a homoeolog-specific transcriptome analysis by discriminating the RNA-Seq reads of each homoeolog of *B. hybridum* and determined the non-parental gene expression patterns. Finally, we assessed homoeolog-specific transcriptome changes in response to heat stress in *B. hybridum* and discussed the differential regulation of the homoeo-transcriptome that is associated with heat stress tolerance in *B. hybridum* inherited from *B. stacei*.

Data Description

Plant materials

Three *Brachypodium* species were used in this study: the allotetraploid *Brachypodium* (*B. hybridum* Bd14-1) and the diploid ancestors (*B. distachyon* Bd21 and *B. stacei* ABR114). The accessions of these species were provided by the National Plant Germplasm System of USDA-ARS, David F. Garvin (USDA-ARS Plant Science Research Unit, University of Minnesota, USA) and Pilar Catalán (Department of Agriculture and

Environment Science, High Polytechnic School of Huesca, University of Zaragoza, Spain). Dry seeds for the plants were incubated on wet filter paper in a Petri dish at 4°C in the dark for 6-7 days to synchronize germination. The germinated seeds were grown in a growth chamber at 25°C under a 16-h day photoperiod (60 $\mu mol \cdot m^{-2} \cdot s^{-1}$) for 4 days. The plants were transplanted to pots filled with autoclaved PRO-MIX BX MYCORRHIZAE (Premier Tech, Quebec, Canada). The potted plants were grown in a growth chamber at 22°C (normal conditions) or 32°C (heat stress conditions) under a 20-h day photoperiod (100 $\mu mol \cdot m^{-2} \cdot s^{-1}$), and watered with 5,000-fold diluted Professional Hyponex 10-30-20 (Hyponex Japan, Osaka, Japan) every three or four days.

Whole genome sequence data

Genomic DNA from the leaf tissues of *B. hybridum* and *B. stacei* were extracted using the DNeasy Plant Mini Kit (QIAGEN K.K, Tokyo, JAPAN). Libraries for single-end DNA sequencing were obtained using the Ion Xpress™ Plus Fragment Library Kits (Life Technologies Japan Ltd, Tokyo, JAPAN), and semiconductor chips that were used for sequencing were prepared using Ion OneTouch 2 System (Life Technologies Japan Ltd, Tokyo, JAPAN) and Ion P1™ Chip v2 (Life Technologies Japan Ltd, Tokyo, JAPAN). The sequencing analyses were performed using an Ion Proton sequencer (Life Technologies Japan Ltd, Tokyo, JAPAN). The genome sequence data of *B. hybridum* and *B. stacei* were archived at DDBJ under the accession number DRA005717 (Additional file 2: Table S1).

RNA sequence data

Shoots and roots from each species grown in a growth chamber for 4 days after synchronized germination were sampled to elucidate the global homoeolog expression patterns in *B. hybridum*. Shoots and most young leaf blades from each species grown in a growth chamber under different temperature conditions for 3 and 15 days, respectively, after being transplanted were sampled to elucidate the homoeolog-specific transcriptional response to heat stress in *B. hybridum*. Total RNA was extracted from each sample using the RNeasy plant mini kit (QIAGEN K.K, Tokyo, JAPAN). Poly(A) RNAs were purified using the NEBNext® Poly(A) mRNA Magnetic Isolation Module (New England Biolabs, MA, USA). The libraries for single-end strand-specific RNA sequencing were obtained using the Ion Total RNA-Seq Kit v.2 (Life Technologies Japan Ltd, Tokyo, JAPAN). Size-selected libraries were purified using Agencourt AMPure XP (Beckman Coulter, CA, USA). Semiconductor chips that were used for sequencing were prepared using the Ion PI™ Hi-Q™ OT2 200 Kit (Life Technologies Japan Ltd, Tokyo, JAPAN), Ion PI™ Hi-Q™ Sequencing 200 Kit (Life Technologies), and Ion P1™ Chip v3 (Life Technologies Japan Ltd, Tokyo, JAPAN). The sequencing analyses were performed using an Ion Proton sequencer (Life Technologies Japan Ltd, Tokyo, JAPAN) with three biological replicates. The RNA-sequencing data were archived at DDBJ under the accession number DRA005699 (Additional file 2: Table S1).

Results

B. hybridum and *B. stacei* showed significant tolerance to long-term heat stress

Unlike *B. distachyon*, *B. hybridum* and *B. stacei* showed significant tolerance to long-term heat stress. The allopolyploid *B. hybridum* and its ancestor *B. stacei* grow in warmer regions, in contrast to the other ancestor *B. distachyon*, which suggests that *B. hybridum* and *B. stacei*, but not *B. distachyon*, might have adapted to high-temperature conditions. To test this hypothesis, we compared the plant biomasses of *B. hybridum*, *B. stacei*, and *B. distachyon* grown under normal (22°C) and heat stress (32°C) conditions at two time points (3 and 15 days after heat stress exposure). At 3 days after exposure to heat stress, the three species showed no differences in their growth (Figure 1A). Conversely, at 15 days after heat exposure, *B. distachyon* showed a significant decrease in fresh weight ($P < 0.01$, *t*-test), whereas *B. hybridum* and *B. stacei* maintained their growth (Figure 1B). This result indicated that *B. hybridum* and *B. stacei* are thermotolerant species and that *B. hybridum* might have inherited this trait from *B. stacei* via allopolyploidization.

A virtual *B. stacei* genome generated from the comparative analysis of homoeologous genomes

By comparing the genomes of *B. hybridum*, *B. stacei*, and *B. distachyon*, we obtained a comprehensive map of the homoeologous single nucleotide polymorphisms (SNPs) in these *Brachypodium* species. This map enabled the distinction of transcripts expressed from each of the homoeologs in *B. hybridum*. To determine polymorphisms between the homoeologous genomes in *B. hybridum*, we sequenced the genomes of *B. stacei* ABR114 and *B. hybridum* Bd14-1 and mapped the reads to the reference genome sequence of *B. distachyon*

Bd21. We found that 85% and 91% of the genomic reads of *B. stacei* and *B. hybridum* mapped to the reference genome and covered 89% and 98% of the genic region of the reference genome, respectively, which suggests high similarity among the homoeologous genomes (Figure 2 and Additional file 2: Table S2). We identified genomic polymorphisms; 11,948,285 SNPs were identified between *B. distachyon* and *B. stacei* and 10,216,010 SNPs between *B. distachyon* and *B. hybridum* (Additional file 2: Table S3). We selected 5,720,539 SNPs to discriminate the homoeologs that were homogenic to the *B. stacei* reads from those that heterogenic to the *B. hybridum* reads (Figure 2, Additional file 1: Figure S2 and Additional file 3). By replacing the nucleotides of the homoeologous SNPs in the *B. distachyon* genome with those in *B. stacei*, we generated virtual homoeolog sequences of *B. stacei* that corresponded to *B. distachyon* counterparts (Additional file 1: Figure S3).

Homoeolog-specific transcriptome analysis represents non-additive and non-parental gene expression patterns in *B. hybridum*

The sequences of the *B. hybridum* transcriptome were sorted by mapping to the *B. distachyon* and virtual *B. stacei* genome, and the expression patterns of the homoeologous genes in the leaf and root tissues under normal conditions were investigated; global homoeolog expression patterns in *B. hybridum* were thus revealed. We sorted approximately 79% of the RNA-Seq reads of *B. hybridum* to the two homoeologous genomes of *B. distachyon* (Bd-homoeologs) and *B. stacei* (Bs-homoeologs) based on their sequence identities after alignments with both genomes (Additional file 1: Figures S4 and S5). The homoeologous transcripts

were almost evenly expressed between the *B. distachyon* and *B. stacei* genomes in the *B. hybridum* transcriptome of the leaf and root tissues under normal conditions (Additional file 1: Figure S5). When we compared the expression patterns of the genes expressed in both the mid-ancestral values (MAVs) (Additional file 1: Figure S4A) and *B. hybridum*, we found that 23% and 26% were non-additively expressed in the *B. hybridum* leaf and root transcriptomes, respectively (the remaining 77% and 74% of these were additively expressed; Figures 3A, B, C and D). Furthermore, 38% and 35% of the genes showed non-ancestral expression patterns in the *B. hybridum* leaf and root transcriptomes, respectively (the remaining 62% and 65% of those genes were inherited ancestral expression patterns; Figures 3A, B, E and F). These data suggest that ~26% and ~38% of the homoeologous gene pairs in *B. hybridum* changed its expression pattern towards non-additive expression and non-parental expression, respectively, after the genome-scale gene duplication via allopolyploidization that occurred more than 1 MYA.

Homoeolog-specific transcriptional response to heat stress in *B. hybridum*

B. distachyon was transcriptionally insensitive to heat after 3 days of exposure to heat stress (32°C), whereas the *B. hybridum* transcriptome noticeably responded to heat stress; this response of *B. hybridum* might be a physiological trait that is inherited from *B. stacei*. When the gene expression patterns were compared between *B. distachyon*, *B. stacei*, and *B. hybridum*, as well as the Bd- and Bs-homoeologs of *B. hybridum*, after 3 days of exposure to heat stress, the transcriptomes of *B. stacei* and *B. hybridum* noticeably changed in response to heat stress compared with those under normal conditions (22°C; Pearson's correlation coefficient (PCC)

0.68–0.75 and 0.87, respectively; Figure 4A). In contrast, *B. distachyon* showed smaller changes in its transcriptome between heat stress and normal conditions (PCC 0.92–0.95; Figure 4A). At 3 days of exposure to heat stress, 5,649 genes and 3,725 gene groups showed significantly higher expression in *B. stacei* and *B. hybridum*, respectively, than in *B. distachyon* (False discovery rate (FDR) ≤ 0.001). We dissected the expression patterns of the 3,725 gene groups from *B. hybridum* into Bd- and Bs-homoeologs and found that 2,088 gene groups were preferentially expressed by the Bs-homoeologs (Figure 4B). The homoeologs of *B. hybridum* were evenly expressed by both homoeologous genomes, whereas in the 3,725 gene groups that were highly expressed in *B. hybridum* compared with *B. distachyon*, the Bs-homoeologs showed abundant expression compared to the Bd-homoeologs, which suggests ancestral-biased gene expression in the transcriptome of *B. hybridum* in response to heat stress (Additional file 1: Figure S6). Of the 2,088 Bs-homoeologs, 1,791 genes were shared with the 5,649 genes that showed higher expression in *B. stacei* than in *B. distachyon*; these genes included those that were specifically involved in metabolic processes as well as cellular response to stress and damage stimulus (Figures 4C and D). These results suggested that the functions of *B. hybridum* genes that were inherited from *B. stacei* may have contributed to the transcriptional stress response and associated metabolic changes in the *B. hybridum* transcriptome during the earlier response to heat stress.

Early transcriptional responses of the Bs-homoeologs contribute to the maintenance of cellular

functions in *B. hybridum* under long-term heat stress

After 15 days of exposure to heat stress, *B. hybridum* and *B. stacei* maintained their transcriptional states similar to those under normal condition, in contrast to the severely damaged cellular system of *B. distachyon*. When we compared the expression patterns between *B. distachyon*, *B. stacei*, and *B. hybridum* after 15 days of exposure to heat stress, we found that *B. stacei* and *B. hybridum* showed similar expression patterns between stress and normal conditions (PCC 0.95), whereas *B. distachyon* showed remarkable changes in its transcriptome (PCC 0.87) reflecting a severe decrease in its biomass (Figures 1B and 5A). Specifically, genes involved in primary metabolism, such as photosynthesis and metabolite and energy generation, were significantly less represented in *B. distachyon* than in *B. stacei* and in *B. hybridum* under heat stress (Figures 5B and C and Additional file 2: Tables S4 and S5), which likely indicates the physiological sensitivity of its cellular system against heat stress. The number of Bs-homoeologs that were expressed more abundantly than Bd-homoeologs were reduced in gene groups that were highly expressed in *B. hybridum* than in *B. distachyon* at 15 days after exposure to heat stress compared to that found after 3 days of heat stress exposure (Figure 4B and Additional file 1: Figure S7), which suggests that Bs-homoeologs are significantly activated at the earlier phase of transcriptional response to heat stress. We also found significantly higher expression of Bs-homoeologs and genes in *B. stacei* encoding A2-type heat shock transcription factor (*HsfA2*) as well as putative *HsfA2*-targeted genes such as heat shock protein 101 (*Hsp101*) and ascorbate peroxidase 2-like (*APX2*-like; homologs of Arabidopsis *APX1* and rice *APX2*), which are known as key factors in response to

heat [41-43], comparing to expression of the counterparts in *B. distachyon* at 3 days after exposure to 32°C (Additional file 1: Figure S8). These results suggested that the earlier response to heat stress that was specific to the Bs-homoeologs likely contributed to the maintenance of their cellular homeostasis under the long heat exposure, which might be associated with their heat stress tolerance capacity.

Discussion

Evolutionary non-additive gene expression in the hybrid grass could boost its invasion

Our transcriptome analysis comprehensively provided the non-additive gene expression pattern in the hybrid species *B. hybridum*; this pattern suggests that the global transcriptional changes in its leaves and roots evolved through allopolyploidization. The gene ontology (GO) analysis of non-additively expressed genes in *B. hybridum* relative to both ancestral species showed an overrepresentation of genes that were involved in response to stimulus and abiotic stimulus (Additional file 2: Tables S6 and S7), which suggests that increased expression divergence of genes related to such functions enhanced their ability to respond to environmental change and to adapt to ecological niches. An increased expression divergence of such genes has also been reported in *Arabidopsis* allotetraploid *A. suecica* and wheat [44, 45]. Although such examples have been reported in few species, future progress in transcriptome datasets of hybrid species and their ancestors might enable the application of universal rules for determining transcriptional changes when new hybrid species are generated. Allopolyploid species have long been hypothesized to possess greater

environmental adaptation to wider niches than their ancestors. Specifically, enhanced heterozygosity and genetic diversity resulting from the hybridization of multiple diverged genomes have been thought to upgrade stress tolerances and contribute to the expansion of niches in hybrid species [46-48]. Although *B. hybridum* shows the largest niche overlap compared with its diploid ancestral species, it shows a niche breadth that is smaller than that of *B. distachyon* and slightly greater than that of *B. stacei* [34, 35]. However, *B. hybridum* could also successfully colonize other non-native world regions [34], which suggests its greater ecological tolerance compared with that of other diploids. With the non-additive transcriptional changes in *B. hybridum*, the expanded diversity of gene expression might contribute to the colonization of non-native areas while avoiding inbreeding depression and might boost its diversifying selection [49, 50].

Homoeolog-specific gene expression causes acclimation to heat stress

Although no differences in visible traits were found between the three *Brachypodium* species at 3 days after exposure to heat stress, significant differences in physiological traits that were revealed from their transcriptomes were found in the early stage of heat exposure. The comprehensive list of Bs-homoeolog and *B. stacei* genes with significantly higher expression than the *B. distachyon* genes at 3 days after exposure to 32°C included genes involved in the regulation of acclimation to heat in plants, such as heat shock transcription factor (Hsf), heat shock protein (Hsp), and DNAJ [51-54] (Additional file 2: Table S8). Thus, the functions of *B. hybridum* in acclimating to heat are likely specifically inherited from *B. stacei* via allopolyploidization (Figure 6). Previous evolutional and ecological studies suggested that *B. distachyon*

could have adapted to different environments by diverging from *B. stacei* [34, 35]. The distribution areas of *B. distachyon* and *B. stacei* suggested that the two *Brachypodium* species have adapted to cooler and wetter areas and to warmer and drier areas, respectively, via their diversification [34]. In *B. distachyon*, the heat acclimation function might have been lost during its adaptation process after branching from the most recent common ancestor of these diploids. The adaptive trait to heat stress in the *B. stacei* genome could influence the survival of both individual plants and hybrid progeny under heat stress conditions.

Methods

Fresh weight measurement

The aboveground parts of each species that were grown under normal and heat stress conditions at 3 and 15 days after stress exposure were used to measure fresh weight. Twelve individuals were used for the measurement. A *t*-test was used for statistical comparisons between the plants grown under the different conditions. The significance threshold was set at $P < 0.01$.

Coverage calculation and SNP calling

The genome sequence reads were trimmed using Trimmomatic (v.0.32) [55] with the -thread 2 LEADING: 20 TRAILING: 20 MINLEN: 50 commands. The trimmed reads were mapped to the reference genome sequence of Bd21 downloaded from Phytozome (Bdistachyon_314_v3.0.fa.gz, http://genome.jgi.doe.gov/pages/dynamicOrganismDownload.jsf?organism=Bdistachyon) using TMAP

(v.3.1.4; Life Technologies Japan Ltd, Tokyo, JAPAN) with the mapall -n 4 -v -Y -u -o 2 stage1 map4 commands. The coverage of genome sequence reads on the reference genome was calculated by removing the non-mapped reads and merging multiple mapping data of the same species from the raw mapping data using SAMTools (v.0.1.19) [56] with the view –F 4 and merge commands. The merged data were subjected to coverage calculation using the genomeCoverageBed in BEDTools (v.2.20.1) [57]. The SNPs between the genome sequence reads and reference genome were called by removing the non-mapped reads and possible duplicate reads and merging the multiple mapping data of the same species from the raw mapping data using SAMTools with the view -F 4, rmdup, and merge commands. The merged data were subjected to SNP calling using VarScan (v.2.3.7) [58] with the pileup2snp –p-value 0.01 commands.

Homoeologous SNP identification and virtual *B. stacei* genome construction

Common homogenic SNPs between *B. distachyon* and *B. stacei* and heterogenic SNPs between *B. distachyon* and *B. hybridum* were identified as homoeologous SNPs (Additional file 3); these were used to identify homoeologous genomes in *B. hybridum*. A virtual *B. stacei* genome was constructed by replacing the nucleotides of the homoeologous SNPs in the *B. distachyon* genome with those in *B. stacei*. The process was conducted using original Perl script (Additional file 4).

Read count and reads per million calculation

The RNA-Seq reads were trimmed using Trimmomatic with the -thread 4 LEADING: 20 TRAILING: 20 MINLEN: 50 commands. The trimmed reads were mapped to Bd21 and the virtual *B. stacei* genome using

TMAP with the mapall -n 4 -v -Y -u -o 2 stage1 map4 commands; *B. distachyon* reads were mapped to the Bd21 genome, *B. stacei* reads were mapped to the virtual *B. stacei* genome, and *B. hybridum* reads were mapped to both the Bd21 and virtual *B. stacei* genomes. The expression levels of the homoeologous genes in *B. hybridum* were quantified by classifying the RNA-Seq reads into the following three groups based on their sequence identities in the alignments with both genomes using the original Perl script (Additional file 5): *B. distachyon* genome origin reads, *B. stacei* gnome origin reads, and unclassified reads. The mapping data from the *B. distachyon* reads, *B. stacei* reads, *B. distachyon* genome origin reads, and *B. stacei* genome origin reads were subjected to read count using featureCounts (v.1.4.6) [59] with the gene structural annotation of Bd21 (Bdistachyon_314_v3.1.gene_exons.gff3.gz, http://genome.jgi.doe.gov/pages/dynamicOrganismDownload.jsf?organism=Bdistachyon). As with the MAV, the total read count data from the *B. distachyon* and *B. stacei* reads was used as well as the entire *B. hybridum* gene group expression value, the total read count data of the *B. distachyon* genome origin reads, and the *B. stacei* genome origin reads. The reads per million mapped reads (RPM) values were calculated for all annotated genes based on the read count data. Genes with the RPM value ≥ 1 in all three replicates were defined as expressed.

Deferentially expressed genes analysis

Differentially expressed genes between the three *Brachypodium* species, homoeologous gene pairs, and growth conditions were identified using DESeq2 package (v.1.10.1) [60] in R (v.3.2.4) with the Wald test

based on the read count data. FDR for each comparison was calculated by adjusting p-value by the Benjamini-Hochberg procedure. Genes with an FDR ≤ 0.001 were defined as differentially expressed.

Gene Ontology enrichment analysis

GO terms for the *B. distachyon* genes were used from the gene annotation information downloaded from Phytozome (Bdistachyon_314_v3.1.annotation_info.txt, http://genome.jgi.doe.gov/pages/dynamicOrganismDownload.jsf?organism=Bdistachyon). Additional GO terms were associated with the *B. distachyon* genes using GO terms that related to transcripts for *A. thaliana* and rice in the “Best-hit-arabi-name” and “Best-hit-rice-name” row in the annotation file. The GO terms for *A. thaliana* and rice were used from the gene annotation information downloaded from Phytozome (Athaliana_167_TAIR10.annotation_info.txt, http://genome.jgi.doe.gov/pages/dynamicOrganismDownload.jsf?organism=Athaliana, and Osativa_204_v7.0.annotation_info, http://genome.jgi.doe.gov/pages/dynamicOrganismDownload.jsf?organism=Osativa). To help reduce bias, GO terms that were assigned to more than 3000 *Brachypodium* genes were excluded. Enriched GO terms for selected genes were identified using BLAST2GO (v.3.3.5) [61] with the Fisher`s exact test using the following settings: P-Value Filter Value, 0.05 and P-Value Filter Mode, FDR. The information of GO annotations of the *B. distachyon* genes is provided in Additional file 6.

Availability of supporting data

All sequencing data were archived at DDBJ under the accession number DRA005717and DRA005699. The other datasets supporting the results of this article are included additional files. Additional file 1 and 2 provide Supplementary Figures and Supplementary Tables, respectively. Additional file 3 provides the homoeologous SNPs dataset used in this study. Additional file 4 and 5 provide the original Perl script codes used in this study. Additional file 6 provides the GO annotations of the *B. distachyon* genes used in this study.

Additional files

Additional file 1: Supplementary Figures

Figure S1. Phylogenetic relationships among the three *Brachypodium* species.

Figure S2. Results of homoeologous SNP identification.

Figure S3. Overview of genomic sequence data analysis.

Figure S4. Overview of RNA-Seq data analysis.

Figure S5. Classification of the read origin of the *B. hybridum* RNA reads from leaf and root tissues.

Figure S6. Log_2 Fold-change distribution of homoeolog expression in *B. hybridum* under heat stress condition for 3 days.

Figure S7. Gene expression profiles of the Bd- and Bs-homoeologs in gene groups showing significantly higher expression in *B. hybridum* than in *B. distachyon* under heat stress condition at 15 days after stress exposure.

Figure S8. Gene expression profiles of the *Brachypodium HsfA2* and putative *HsfA2*-targeted genes at 3 days after heat stress exposure.

Additional file 2: Supplementary Tables

Table S1. Summary of the sequencing analysis and accession numbers

Table S2. Summary of the whole genome sequencing analysis and mapping results.

Table S3. Results of SNP calling.

Table S4. Enriched GO terms in the biological process ontology of genes showing significantly higher expression in *B. stacei* than in *B. distachyonon* at 15 days after heat stress exposure

Table S5. Enriched GO terms in the biological process ontology of gene groups showing significantly higher expression in *B. hybridum* than in *B. distachyonon* at 15 days after heat stress exposure

Table S6. Enriched GO terms in the biological process ontology of genes showing non-additive expression in *B. hybridum* leaf under normal condition.

Table S7. Enriched GO terms in the biological process ontology of genes showing non-additive expression in *B. hybridum* root under normal condition.

Table S8. Genes showing higher expression in the *B. stacei* genome than in the *B. distachyon* genome in the

three *Brachypodium* species under heat stress at 3 days after stress exposure.

Additional file 3: Homoeologous SNPs dataset used in this study

Additional file 4: Original perl script code used to construct the virtual *B. stacei* genome by replacing the nucleotides of the homoeologous SNPs in the *B. distachyon* genome with those in *B. stacei*

Additional file 5: Original perl script code used to classify the RNA-Seq reads of *B. hybridum* into the *B. distachyon* genome origin reads, *B. stacei* gnome origin reads, and unclassified reads

Additional file 6: GO annotations of the *B. distachyon* genes used in this study

Abbreviations

DNA, Deoxyribonucleic acid; FDR, False discovery rate; GO, Gene ontology; MAV, Mid-ancestral value; MAY, Million years ago; PCC, Pearson's correlation coefficient; RNA, Ribonucleic acid; RPM, Reads per million mapped reads; SNP, Single nucleotide polymorphism

Competing interests

The authors declare that they have no competing interests.

Author contributions

KT and KM designed the work. KT and YO grew and sampled the plants. MS, YU-Y and YO generated the

genome sequencing data. KT and KI generated the RNA sequencing data. KT performed in silico analyses using the genome and RNA sequencing data. KM directed this study. KT and KM wrote the manuscript with all authors.

Acknowledgments

The authors thank the National Plant Germplasm System of USDA-ARS, David F. Garvin and Pilar Catalán for providing *Brachypodium* seeds. This work was partially supported by grants-in-aid for Young Scientists (A) (grant no. 26712003 to KM) from the Japan Society for the Promotion of Science (JSPS) and by funds to KM from the Advanced Low Carbon Technology Research and Development Program (ALCA, J2013403) of the Japan Science and Technology Agency (JST). This work was also supported by RIKEN Junior Research Associate Program.

References

1. Soltis PS and Soltis DE. Polyploidy and genome evolution. Berlin ; New York: Springer Verlag; 2012.
2. Comai L. The advantages and disadvantages of being polyploid. Nat Rev Genet. 2005;6 11:836-46.
3. Leitch AR and Leitch IJ. Perspective - Genomic plasticity and the diversity of polyploid plants. Science. 2008;320 5875:481-3.

4. Bowman JL, Floyd SK and Sakakibara K. Green genes-comparative genomics of the green branch of life. Cell. 2007;129 2:229-34.

5. Adams KL and Wendel JF. Polyploidy and genome evolution in plants. Curr Opin Plant Biol. 2005;8 2:135-41.

6. Segraves KA. The effects of genome duplications in a community context. New Phytol. 2017.

7. Bowers JE, Chapman BA, Rong J and Paterson AH. Unravelling angiosperm genome evolution by phylogenetic analysis of chromosomal duplication events. Nature. 2003;422 6930:433-8.

8. Otto SP. The evolutionary consequences of polyploidy. Cell. 2007;131 3:452-62.

9. Jiao Y, Wickett NJ, Ayyampalayam S, Chanderbali AS, Landherr L, Ralph PE, et al. Ancestral polyploidy in seed plants and angiosperms. Nature. 2011;473 7345:97-100.

10. Soltis PS and Soltis DE. The role of hybridization in plant speciation. Annu Rev Plant Biol. 2009;60:561-88.

11. Fawcett JA, Maere S and Van de Peer Y. Plants with double genomes might have had a better chance to survive the Cretaceous-Tertiary extinction event. Proc Natl Acad Sci U S A. 2009;106 14:5737-42.

12. Vanneste K, Baele G, Maere S and Van de Peer Y. Analysis of 41 plant genomes supports a wave of successful genome duplications in association with the Cretaceous-Paleogene boundary. Genome Res. 2014;24 8:1334-47.

13. Chen ZJ. Molecular mechanisms of polyploidy and hybrid vigor. Trends Plant Sci. 2010;15 2:57-71.

14. Chen ZJ. Genomic and epigenetic insights into the molecular bases of heterosis. Nat Rev Genet. 2013;14 7:471-82.

15. Chen ZJ and Birchler JA. Polyploid and hybrid genomics. Ames, Iowa: Wiley-Blackwell; 2013.

16. Solhaug EM, Ihinger J, Jost M, Gamboa V, Marchant B, Bradford D, et al. Environmental Regulation of Heterosis in the Allopolyploid Arabidopsis suecica. Plant Physiol. 2016;170 4:2251-63.

17. Maricle BR, Crosier JJ, Bussiere BC and Lee RW. Respiratory enzyme activities correlate with anoxia tolerance in salt marsh grasses. J Exp Mar Bio Ecol. 2006;337 1:30-7.

18. Ainouche ML, Fortune PM, Salmon A, Parisod C, Grandbastien MA, Fukunaga K, et al. Hybridization, polyploidy and invasion: lessons from Spartina (Poaceae). Biol Invasions. 2009;11 5:1159-73.

19. Dubcovsky J and Dvorak J. Genome plasticity a key factor in the success of polyploid wheat under domestication. Science. 2007;316 5833:1862-6.

20. Yang C, Zhao L, Zhang H, Yang Z, Wang H, Wen S, et al. Evolution of physiological responses to salt stress in hexaploid wheat. Proc Natl Acad Sci U S A. 2014;111 32:11882-7.

21. Stebbins GL. Variation and evolution in plants. New York,: Columbia University Press; 1950.

22. Stebbins GL. Chromosomal evolution in higher plants. Reading, Mass.: Addison-Wesley; 1971.

23. Grover CE, Gallagher JP, Szadkowski EP, Yoo MJ, Flagel LE and Wendel JF. Homoeolog expression

bias and expression level dominance in allopolyploids. New Phytol. 2012;196 4:966-71.

24. Yoo MJ, Liu X, Pires JC, Soltis PS and Soltis DE. Nonadditive gene expression in polyploids. Annu Rev Genet. 2014;48:485-517.

25. Chen ZJ and Ni Z. Mechanisms of genomic rearrangements and gene expression changes in plant polyploids. Bioessays. 2006;28 3:240-52.

26. Chen ZJ. Genetic and epigenetic mechanisms for gene expression and phenotypic variation in plant polyploids. Annu Rev Plant Biol. 2007;58:377-406.

27. Yoo MJ, Szadkowski E and Wendel JF. Homoeolog expression bias and expression level dominance in allopolyploid cotton. Heredity (Edinb). 2013;110 2:171-80.

28. Li A, Liu D, Wu J, Zhao X, Hao M, Geng S, et al. mRNA and Small RNA Transcriptomes Reveal Insights into Dynamic Homoeolog Regulation of Allopolyploid Heterosis in Nascent Hexaploid Wheat. Plant Cell. 2014;26 5:1878-900.

29. Zhang D, Pan Q, Tan C, Zhu B, Ge X, Shao Y, et al. Genome-Wide Gene Expressions Respond Differently to A-subgenome Origins in Brassica napus Synthetic Hybrids and Natural Allotetraploid. Front Plant Sci. 2016;7:1508.

30. Madlung A. Polyploidy and its effect on evolutionary success: old questions revisited with new tools. Heredity (Edinb). 2013;110 2:99-104.

31. Akama S, Shimizu-Inatsugi R, Shimizu KK and Sese J. Genome-wide quantification of homeolog

expression ratio revealed nonstochastic gene regulation in synthetic allopolyploid Arabidopsis. Nucleic Acids Res. 2014;42 6:e46.

32. Paape T, Hatakeyama M, Shimizu-Inatsugi R, Cereghetti T, Onda Y, Kenta T, et al. Conserved but Attenuated Parental Gene Expression in Allopolyploids: Constitutive Zinc Hyperaccumulation in the Allotetraploid Arabidopsis kamchatica. Mol Biol Evol. 2016;33 11:2781-800.

33. Lopez-Alvarez D, Lopez-Herranz ML, Betekhtin A and Catalan P. A DNA barcoding method to discriminate between the model plant Brachypodium distachyon and its close relatives B. stacei and B. hybridum (Poaceae). PLoS One. 2012;7 12:e51058.

34. Lopez-Alvarez D, Manzaneda AJ, Rey PJ, Giraldo P, Benavente E, Allainguillaume J, et al. Environmental niche variation and evolutionary diversification of the Brachypodium distachyon grass complex species in their native circum-Mediterranean range. Am J Bot. 2015;102 7:1073-88.

35. Catalan P, Muller J, Hasterok R, Jenkins G, Mur LA, Langdon T, et al. Evolution and taxonomic split of the model grass Brachypodium distachyon. Ann Bot. 2012;109 2:385-405.

36. Catalan P, Chalhoub B, Chochois V, Garvin DF, Hasterok R, Manzaneda AJ, et al. Update on the genomics and basic biology of Brachypodium International Brachypodium Initiative (IBI). Trends Plant Sci. 2014;19 7:414-8.

37. Catalan P, Lopez-Alvarez D, Bellosta C and Villar L. Updated taxonomic descriptions, iconography, and habitat preferences of Brachypodium distachyon, B-stacei, and B-hybridum (Poaceae). An

Jardin Bot Madrid. 2016;73 1.

38. Betekhtin A, Jenkins G and Hasterok R. Reconstructing the Evolution of Brachypodium Genomes Using Comparative Chromosome Painting. PLoS One. 2014;9 12:e115108.

39. Vogel J. Genetics and genomics of Brachypodium. New York, NY: Springer Science+Business Media; 2016.

40. Dinh Thi VH, Coriton O, Le Clainche I, Arnaud D, Gordon SP, Linc G, et al. Recreating Stable Brachypodium hybridum Allotetraploids by Uniting the Divergent Genomes of B. distachyon and B. stacei. PLoS One. 2016;11 12:e0167171.

41. Schramm F, Ganguli A, Kiehlmann E, Englich G, Walch D and von Koskull-Doring P. The heat stress transcription factor HsfA2 serves as a regulatory amplifier of a subset of genes in the heat stress response in Arabidopsis. Plant Mol Biol. 2006;60 5:759-72.

42. Charng YY, Liu HC, Liu NY, Chi WT, Wang CN, Chang SH, et al. A heat-inducible transcription factor, HsfA2, is required for extension of acquired thermotolerance in Arabidopsis. Plant Physiol. 2007;143 1:251-62.

43. Chauhan H, Khurana N, Agarwal P and Khurana P. Heat shock factors in rice (Oryza sativa L.): genome-wide expression analysis during reproductive development and abiotic stress. Mol Genet Genomics. 2011;286 2:171-87.

44. Kim ED and Chen ZJ. Unstable transcripts in Arabidopsis allotetraploids are associated with

nonadditive gene expression in response to abiotic and biotic stresses. PLoS One. 2011;6 8:e24251.

45. Chague V, Just J, Mestiri I, Balzergue S, Tanguy AM, Huneau C, et al. Genome-wide gene expression changes in genetically stable synthetic and natural wheat allohexaploids. New Phytol. 2010;187 4:1181-94.

46. Lowry E and Lester SE. The biogeography of plant reproduction: potential determinants of species' range sizes. J Biogeogr. 2006;33 11:1975-82.

47. te Beest M, Le Roux JJ, Richardson DM, Brysting AK, Suda J, Kubesova M, et al. The more the better? The role of polyploidy in facilitating plant invasions. Ann Bot. 2012;109 1:19-45.

48. Marchant DB, Soltis DE and Soltis PS. Patterns of abiotic niche shifts in allopolyploids relative to their progenitors. New Phytol. 2016;212 3:708-18.

49. Bakker EG, Montgomery B, Nguyen T, Eide K, Chang J, Mockler TC, et al. Strong population structure characterizes weediness gene evolution in the invasive grass species Brachypodium distachyon. Mol Ecol. 2009;18 12:2588-601.

50. Meimberg H, Rice KJ, Milan NF, Njoku CC and McKay JK. Multiple origins promote the ecological amplitude of allopolyploid Aegilops (Poaceae). Am J Bot. 2009;96 7:1262-73.

51. Song LL, Jiang YL, Zhao HQ and Hou MF. Acquired thermotolerance in plants. Plant Cell Tiss Org. 2012;111 3:265-76.

52. Driedonks N, Xu J, Peters JL, Park S and Rieu I. Multi-Level Interactions Between Heat Shock

Factors, Heat Shock Proteins, and the Redox System Regulate Acclimation to Heat. Front Plant Sci. 2015;6:999.

53. Jacob P, Hirt H and Bendahmane A. The heat-shock protein/chaperone network and multiple stress resistance. Plant Biotechnol J. 2017;15 4:405-14.

54. Ohama N, Sato H, Shinozaki K and Yamaguchi-Shinozaki K. Transcriptional Regulatory Network of Plant Heat Stress Response. Trends Plant Sci. 2017;22 1:53-65.

55. Bolger AM, Lohse M and Usadel B. Trimmomatic: a flexible trimmer for Illumina sequence data. Bioinformatics. 2014;30 15:2114-20.

56. Li H, Handsaker B, Wysoker A, Fennell T, Ruan J, Homer N, et al. The Sequence Alignment/Map format and SAMtools. Bioinformatics. 2009;25 16:2078-9.

57. Quinlan AR. BEDTools: The Swiss-Army Tool for Genome Feature Analysis. Curr Protoc Bioinformatics. 2014;47:11 2 1- 2 34.

58. Koboldt DC, Chen K, Wylie T, Larson DE, McLellan MD, Mardis ER, et al. VarScan: variant detection in massively parallel sequencing of individual and pooled samples. Bioinformatics. 2009;25 17:2283-5.

59. Liao Y, Smyth GK and Shi W. featureCounts: an efficient general purpose program for assigning sequence reads to genomic features. Bioinformatics. 2014;30 7:923-30.

60. Love MI, Huber W and Anders S. Moderated estimation of fold change and dispersion for RNA-seq

data with DESeq2. Genome Biol. 2014;15 12:550.

61. Conesa A, Gotz S, Garcia-Gomez JM, Terol J, Talon M and Robles M. Blast2GO: a universal tool for annotation, visualization and analysis in functional genomics research. Bioinformatics. 2005;21 18:3674-6.

Figure legends

Figure 1. Effect of heat stress on the growth of the three *Brachypodium* species.

Fresh weight of *B. distachyon*, *B. stacei*, and *B. hybridum* grown under normal (22°C) and heat stress (32°C) conditions at 3 (**A**) and 15 (**B**) days after stress exposure. Each bar represents the mean value ± standard deviation for twelve individuals. Statistical differences are indicated by an asterisk ($P < 0.01$, *t*-test). Bd, *B. distachyon*; Bs, *B. stacei*; Bh, *B. hybridum*.

Figure 2. Summary of genomic sequence data.

Mapping results and polymorphic features of the genomic reads from *B. hybridum* and *B. stacei* are superimposed to the reference genome sequence of Bd21 (100 kilobase sliding windows). (1) Number of homoeologous SNPs. (2) Number of heterogenic SNPs between *B. distachyon* and *B. hybridum*. (3) The average number of *B. hybridum* reads mapped to the reference genome. (4) Number of homogenic SNPs between *B. distachyon* and *B. stacei*. (5) The average number of *B. stacei* reads mapped to the reference

genome. (6) Density distribution of the Bd21 annotated genes.

Figure 3. Global gene expression patterns in *B. hybridum* compared with that in the ancestors. (**A** and **B**) A Venn diagram of genes expressed in the MAV (left) and in *B. hybridum* (right) in leaf (**A**) and root (**B**) tissues. (**C** and **D**) Additive and non-additive gene expression patterns in leaf (**C**) and root (**D**) tissues of *B. hybridum*. Genes expressed in both the MAV and *B. hybridum* (17,256 genes) were analysed. (**E** and **F**) Ancestral and non-ancestral gene expression patterns in leaf (**E**) and root (**F**) tissues in *B. hybridum*. Genes expressed in both the MAV and *B. hybridum* (18,021 genes) were analysed. The "=" indicates that the gene expression level is not significantly different between ancestors, MAV and *B. hybridum*, and homoeologs. The ">" and "<" indicates that the gene expression level is significantly different between ancestors, MAV and *B. hybridum*, and homoeologs. The significance threshold is set at FDR ≤ 0.001. Bd, *B. distachyon*; Bs, *B. stacei*; MAV, Mid-ancestral value; BhBd, Bd-homoeologs in *B. hybridum*; BhBs, Bs-homoeologs in *B. hybridum*; Bh, *B. hybridum*.

Figure 4. Heat stress response in *B. hybridum* as analysed using the Bs-homoeologs. (**A**) A heatmap of Pearson's correlation coefficient based on the gene expression profiles of each species at 3 days after heat stress exposure. The Pearson correlation coefficient values between different conditions are shown in yellow squares. (**B**) Gene expression profiles of the Bd- and Bs-homoeologs in gene groups showing

significantly higher expression in *B. hybridum* than in *B. distachyon* under heat stress condition at 3 days after stress exposure. Each dot represents the average expression value of three biological replicates. Blue dots represent genes showing significantly higher expression in Bd-homoeologs than in Bs-homoeologs and red dots represent genes showing significantly higher expression in Bs-homoeologs than in Bd-homoeologs (FDR ≤ 0.001). (**C**) Genes showing significantly higher expression in the *B. stacei* genome than in the *B. distachyon* genome in the three *Brachypodium* species under heat stress condition at 3 days after stress exposure. The left circle represents genes showing significantly higher expression in *B. stacei* than in *B. distachyon*. The right circle represents genes showing significantly higher expression in Bs-homoeologs than in Bd-homoeologs in gene groups showing significantly higher expression in *B. hybridum* than in *B. distachyon*. (**D**) Enriched GO terms in the biological process ontology of 1,791 intersectional genes in the Venn diagram shown in (**C**). Bd, *B. distachyon*; Bs, *B. stacei*; BhBd, Bd-homoeologs in *B. hybridum*; BhBs, Bs-homoeologs in *B. hybridum*; Bh, *B. hybridum*.

Figure 5. Maintenance of transcriptional states in *B. hybridum* and *B. stacei* under long-term heat stress. (**A**) A heatmap of Pearson's correlation coefficient based on the gene expression profiles of each species at 15 days after heat stress exposure. The Pearson correlation coefficient values between different conditions are marked by yellow squares. (**B** and **C**) Top 10 enriched GO terms in the biological process ontology of genes showing significantly higher expression in *B. stacei* than in *B. distachyon* (**B**) and gene groups showing

significantly higher expression in *B. hybridum* than in *B. distachyon* (**C**) at 15 days after heat stress exposure. All enriched GO terms for these genes are shown in additional file 2: Table S4 and S5. Bd, *B. distachyon*; Bs, *B. stacei*; BhBd, Bd-homoeologs in *B. hybridum*; BhBs, Bs-homoeologs in *B. hybridum*; Bh, *B. hybridum*.

Figure 6. A model of phenotype and transcriptome of the three *Brachypodium* species under long-term heat stress.

Bd, *B. distachyon*; Bs, *B. stacei*; BhBd, Bd-homoeologs in *B. hybridum*; BhBs, Bs-homoeologs in *B. hybridum*; Bh, *B. hybridum*.

Figure 1

Click here to download Figure Fig1.pdf

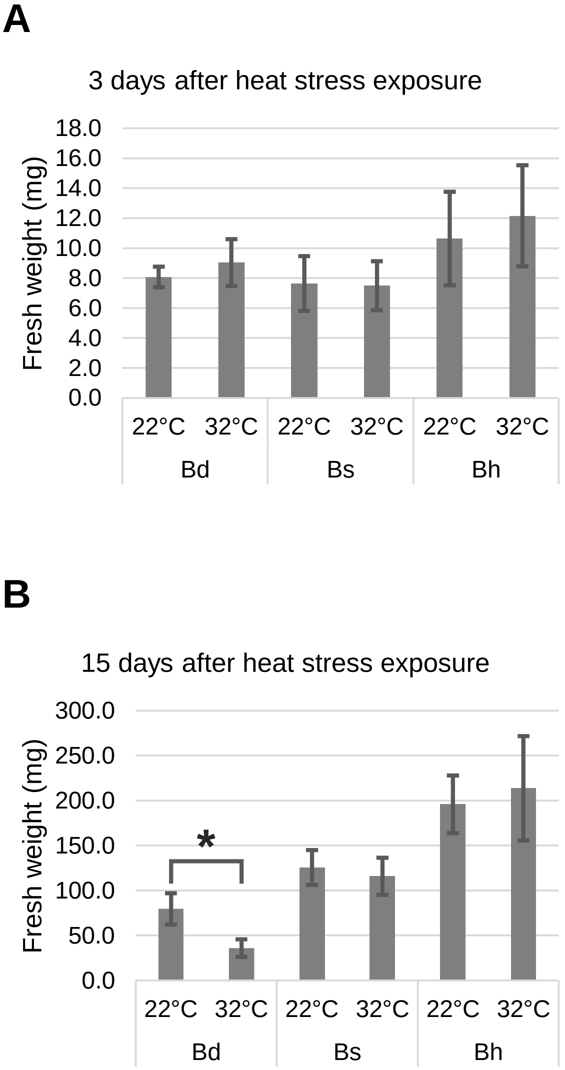

Figure 2

Click here to download Figure Fig2.pdf

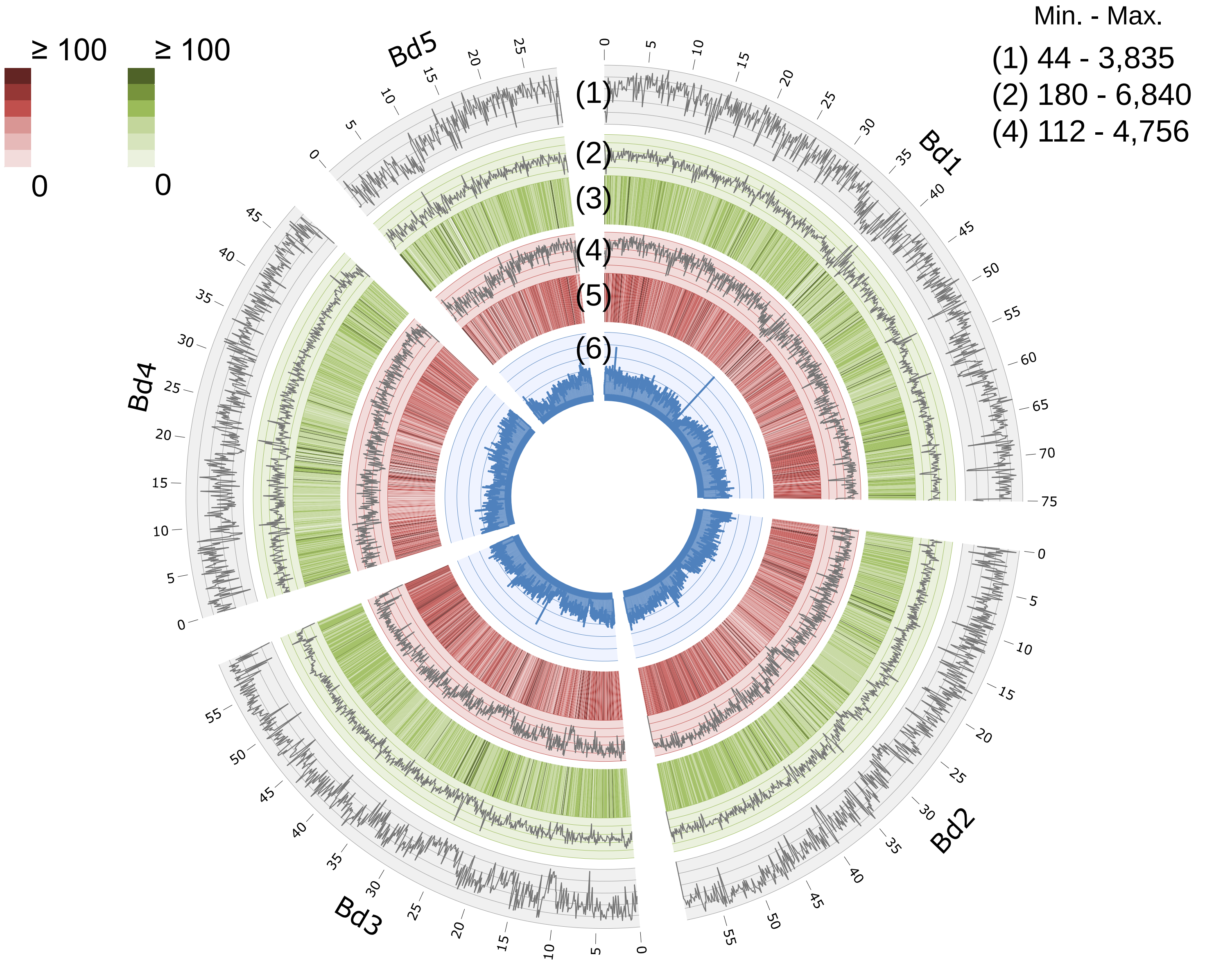

Figure 3

Click here to download Figure Fig3.pdf

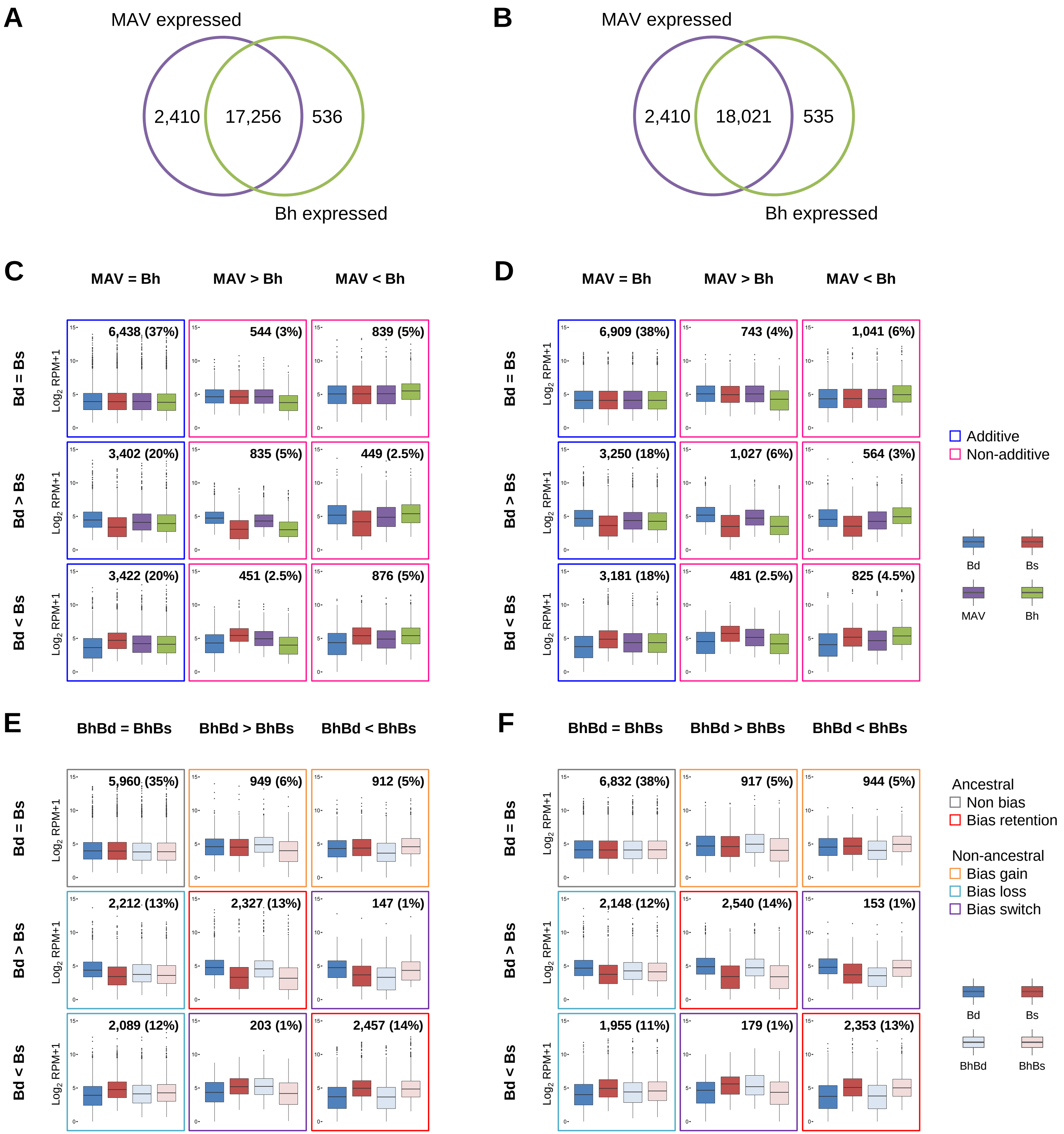

Figure 4 Click here to download Figure Fig4.pdf

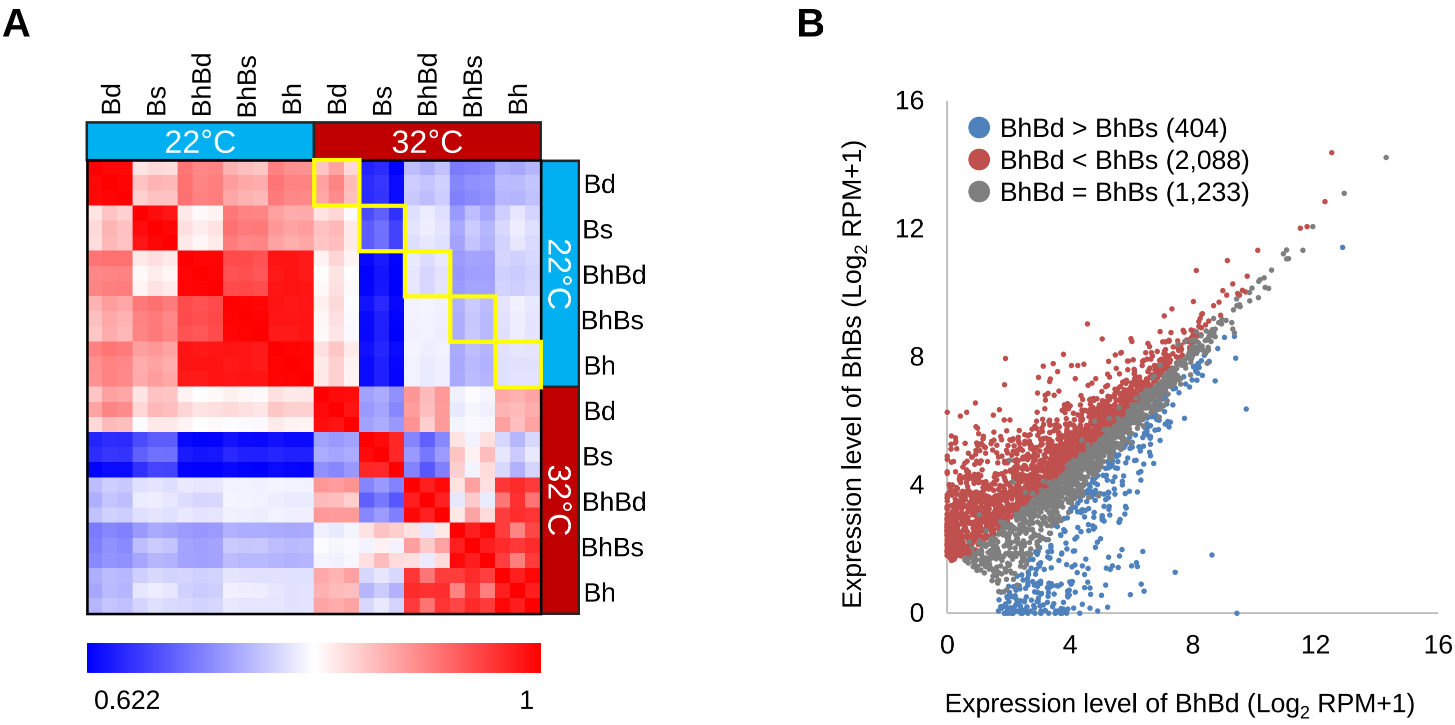

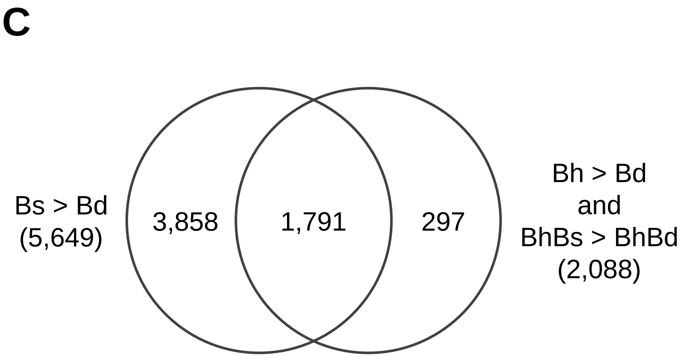

D

Genes showing higher expression in the Bs genome than in the Bd genome in the three *Brachypodium* species (1,791)

GO-ID	Term	FDR
GO:0006259	DNA metabolic process	8.26E-03
GO:0006974	cellular response to DNA damage stimulus	1.99E-02
GO:0006796	phosphate-containing compound metabolic process	2.22E-02
GO:0006793	phosphorus metabolic process	2.29E-02
GO:0033554	cellular response to stress	2.74E-02
GO:0006281	DNA repair	3.02E-02

Figure 5

Click here to download Figure Fig5.pdf

A

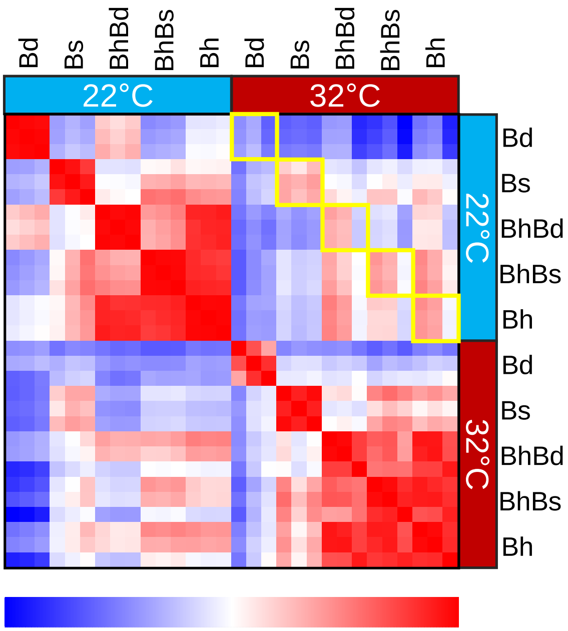

B

Genes showing higher expression in Bs than in Bd (5,517)

GO-ID	Term	FDR
GO:0015979	photosynthesis	1.03E-12
GO:0044710	single-organism metabolic process	3.46E-12
GO:0006091	generation of precursor metabolites and energy	6.73E-09
GO:0044281	small molecule metabolic process	5.69E-07
GO:0044711	single-organism biosynthetic process	6.37E-06
GO:0005975	carbohydrate metabolic process	4.21E-05
GO:0009765	photosynthesis, light harvesting	6.85E-05
GO:0044283	small molecule biosynthetic process	1.35E-04
GO:0006082	organic acid metabolic process	2.44E-04
GO:0051186	cofactor metabolic process	3.85E-04

C

Gene groups showing higher expression in Bh than in Bd (4,009)

GO-ID	Term	FDR
GO:0044710	single-organism metabolic process	4.06E-15
GO:0015979	photosynthesis	2.61E-14
GO:0006091	generation of precursor metabolites and energy	2.63E-14
GO:0044281	small molecule metabolic process	2.94E-08
GO:0044711	single-organism biosynthetic process	6.21E-07
GO:0009765	photosynthesis, light harvesting	1.02E-06
GO:0006629	lipid metabolic process	2.49E-06
GO:0055114	oxidation-reduction process	2.54E-06
GO:0051186	cofactor metabolic process	4.61E-05
GO:0006732	coenzyme metabolic process	8.67E-05

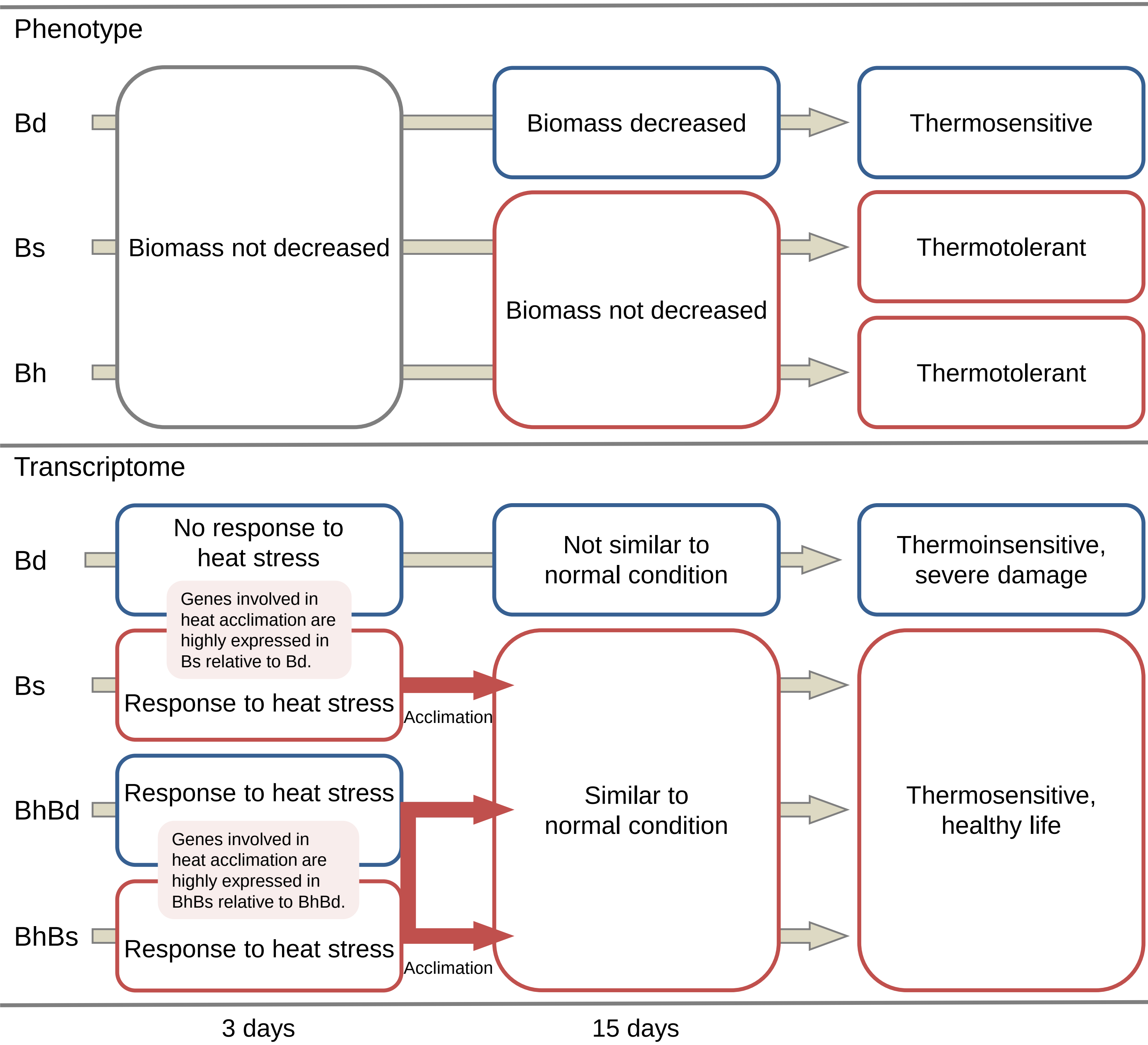
Phenotype
Bd
Bs
Bh
Biomass not decreased
Biomass decreased
Biomass not decreased
Thermosensitive
Thermotolerant
Thermotolerant
Transcriptome
Bd
No response to heat stress
Not similar to normal condition
Thermoinsensitive, severe damage
Genes involved in heat acclimation are highly expressed in Bs relative to Bd.
Bs
Response to heat stress
Acclimation
BhBd
Response to heat stress
Similar to normal condition
Thermosensitive, healthy life
Genes involved in heat acclimation are highly expressed in BhBs relative to BhBd.
BhBs
Response to heat stress
Acclimation
3 days
15 days

Additional file 1: Supplementary Figures

Click here to access/download
Supplementary Material
Additional file 1.pdf

Additional file 2: Supplementary Tables

Click here to access/download
Supplementary Material
Additional file 2.xlsx

Additional file 3: Homoeologous SNPs dataset used in this study

Click here to access/download
Supplementary Material
Additional file 3.txt

Additional file 4: Original perl script code used to construct the virtual <i>B. stacei</i> genome by replacing the nucleotides of the

Click here to access/download

Supplementary Material

Additional file 4.txt

Additional file 5: Original perl script code used to classify the RNA-Seq reads of <i>B. hybridum</i> into the <i>B. distachyon</i>

Click here to access/download
Supplementary Material
Additional file 5.txt

Additional file 6: GO annotations of the <i>B. distachyon</i> genes used in this study

Click here to access/download

Supplementary Material

Additional file 6.txt
