# Supplementary material for: Homoeolog-specific activation of genes for heat acclimation in the allopolyploid grass Brachypodium hybridum
Source: Gigascience. 2018 Mar 8;7(4):giy020. doi: 10.1093/gigascience/giy020 (PMC5915950; doi:10.1093/gigascience/giy020)
Supplement: GIGA-D-17-00181_Revision_1.pdf [file giy020_giga-d-17-00181_revision_1.pdf]

GigaScience

Homoeolog-specific activation for heat acclimation in the allopolyploid grass <i>Brachypodium hybridum</i>

--Manuscript Draft--

**Manuscript Number:**	GIGA-D-17-00181R1
**Full Title:**	Homoeolog-specific activation for heat acclimation in the allopolyploid grass <i>Brachypodium hybridum</i>
**Article Type:**	Research
**Funding Information:**	Japan Society for the Promotion of Science (26712003) — Dr. Keiichi Mochida<br>Japan Science and Technology Agency (J2013403) — Dr. Keiichi Mochida
**Abstract:**	Background: Allopolyploid plants often show wider environmental tolerances than their ancestors; this is expected to be due to the merger of multiple distinct genomes with a fixed heterozygosity. The complex homoeologous gene expression could have been evolutionarily advantageous for the adaptation of allopolyploid plants. Despite multiple previous studies reporting homoeolog-specific gene expression in allopolyploid species, there are no clear examples of homoeolog-specific function in acclimation to a long-term stress condition.<br>Results: We found that the allopolyploid grass <i>Brachypodium hybridum</i> and its ancestor <i>Brachypodium stacei</i> show long-term heat stress tolerance, unlike its other ancestor, <i>Brachypodium distachyon</i>. To understand the physiological traits of <i>B. hybridum</i>, we compared the transcriptome of the three <i>Brachypodium</i> species grown under normal and heat stress conditions. We found that the expression patterns of ~26% and ~38% of the homoeolog groups in <i>B. hybridum</i> changed towards non-additive expression and non-ancestral expression, respectively, under normal condition. Moreover, we found that <i>B. distachyon</i> showed similar expression patterns between normal and heat stress conditions, whereas <i>B. hybridum</i> and <i>B. stacei</i> significantly altered their transcriptome in response to heat after 3 days of stress exposure, and homoeologs that were inherited from <i>B. stacei</i> may have contributed to the transcriptional stress response to heat in <i>B. hybridum</i>. After 15 days of heat exposure, <i>B. hybridum</i> and <i>B. stacei</i> maintained transcriptional states similar to those under normal conditions. These results suggested that an earlier response to heat that was specific to homoeologs originating from <i>B. stacei</i> contributed to cellular homeostasis under long-term heat stress in <i>B. hybridum</i>.<br>Conclusions: Our results provide insights into different regulatory events of the homoeo-transcriptome that are associated with stress acclimation in allopolyploid plants.
**Corresponding Author:**	Keiichi Mochida<br><br>JAPAN
**Corresponding Author Secondary Information:**	
**Corresponding Author's Institution:**	
**Corresponding Author's Secondary Institution:**	
**First Author:**	Kotaro Takahagi
**First Author Secondary Information:**	
**Order of Authors:**	Kotaro Takahagi<br>Komaki Inoue<br>Minami Shimizu

Powered by Editorial Manager® and ProduXion Manager® from Aries Systems Corporation

	Yukiko Uehara-Yamaguchi
	Yoshihiko Onda
	Keiichi Mochida
**Order of Authors Secondary Information:**	

Response to Reviewers:

December 15, 2017

Hans Zauner
Assistant Editor
<i>GigaScience</i>

Dear Dr. Hans Zauner,
We wish to express our appreciation to the editor and reviewers for their insightful comments on our paper. Their suggestions helped us significantly to improve the quality of the manuscript. To address the major concerns from both reviewers, we validated our results through a genomic analysis with B. stacei genome sequence dataset available at Phytozome and verified our results on homoeolog specific gene expressions through a quantification of homoeologous SNPs in cDNA amplicons by using TaqMan assay with a digital PCR. In addition to these major points, we have addressed all the comments from Reviewers #1 and #2 as detailed in a point-by-point list below. We hope the revised manuscript is acceptable for publication in <i>GigaScience</i>.

Sincerely,
Keiichi Mochida
Cellulose Production Research Team, Biomass Engineering Research Division, RIKEN Center for Sustainable Resource Science, 1-7-22 Suehiro-cho, Tsurumi-ku, Yokohama, Kanagawa 230-0045, Japan
Tel: +81-45-503-9111
Fax: +81-45-503-9182
E-mail: keiichi.mochida@riken.jp

Reviewer #1
Major comments
1. The major concern I want to raise is the confused use throughout the manuscript of the different terms regarding the evolution of duplicated genes. As explained in Grover et al. 2012 (and cited in the manuscript), it is essential to distinguish global gene expression from homoeo-specific expression. I would advise the authors to revise the manuscript accordingly and explain in details in the introduction the possible outcomes of allopolyploidy on gene expression. Briefly, one can look at global gene expression and detect events of additivity, genome dominance or transgressivity. Secondly, as the authors put a lot of efforts in detecting the homoeologues, one can compare their expression to a standard; either to a theoretical 50:50 ratio or from the ancestral gene patterns (homologous gene expression levels in both parental species). Thus, comparison of homoeo-specific expression in the allotetraploid can be balanced or unbalanced (skewed towards one or the other parent). As such when the authors disclaim "non-additive and non-parental gene expression patterns in B. hybridum" they could be much more precise when describing gene and homoeo-specific expression patterns. Thereafter, it would be interesting to investigate if specific pathways are enriched in the set of transgressively/over- expressed genes, for instance. I expect that this will reinforce considerably the impact of the manuscript.

We appreciate your comment. As suggested, the possible outcomes of allopolyploidization on gene expression that were addressed in several previous reports and reviews, including Grover et al. (2012) and Yoo et al. (2014), were presented in the Introduction (Lines 80-104).
According to your suggestion, we identified the genes (homoeolog group) that show ELDs and homoeolog expression bias in the leaf and root tissues of B. hybridum. Specifically, we found that the number of homoeolog groups of ELD-Bs is almost twice that of ELD-Bd, suggesting that gene expression patterns of B. stacei are preferentially inherited by the B. hybridum transcriptome in its leaf and root tissues. These results

are presented in newly added Figure 3 and elaborated in the Results section (Lines 233-240 and 254-266).
We were also interested in the enriched functions found in the set of transgressively/over expressed homoeolog groups. To identify enriched function in such genes, we performed GO enrichment analyses and found that GO terms such as primary metabolic processes, primary cellular processes, stress response, transport, localization, and translation were enriched in the transgressively/over expressed homoeolog groups. These results are presented in newly added Tables S5 and S6 and discussed in the Results section (Lines 240-246).

2. The ecology of the three species as well as their evolutionary history is understated in the manuscript. This would help discuss results. For instance, what is the expected time of divergence between both ancestral species and are the mapping results and number of SNPs identified in accordance with expectations? To what extend can the physiology and ecology of the species explain the enrichment of specific pathways.

To examine whether the divergence time between the two ancestral species and the number of SNPs identified in our study is as expected, we calculated the non-synonymous substitution rate (Ka) and synonymous substitution rate (Ks) between the homoeologs, and inferred the divergence time of the diploid ancestors of B. distachyon and B. stacei based on the Ks values. The divergence time was estimated to approximately 6.4 Mya, which falls within the divergence time of B. stacei (5.8–16.4 Mya) previously estimated by Catalan et al. (2012). We have added these results in the Results section (Lines 206-208).
Moreover, we identified 1,741 genes whose Ka/Ks values between homoeologs were greater than 1, supporting their accelerated evolution. We pursed the enrichment functions of those genes and found that genes involved in DNA mismatch repair with GO terms such as proliferating cell nuclear antigen complex and DNA polymerase processivity factor activity were enriched in those genes, which suggested that DNA damage tolerance may reflect selection and adaptive divergence between B. distachyon and B. stacei. These results were discussed in the Results section (Lines 208-214).

3. As previously said, the in silico assembly of the B. stacei genome is quite elegant. Nevertheless, I would appreciate some more details on the set of genes detected using the >5M homoeo-SNPs identified. Also, in the results section, authors are using the terms 'genes' and 'gene groups' that are not defined nor used previously.

We assessed the distribution of the SNPs within various genomic regions and applied the data to gain insights into genetic diversity of the two ancestral genomes. We found that 26,986 genes (79% of the annotated genes in the Bd21 genome) contain nonsynonymous variations. Of those, 4,558 genes (13% of the annotated genes in the Bd21 genome) contained sever variations in the B. stacei genome: 683 genes contained no start codon, 2,980 genes had the stop codon, and 895 genes lacked the stop codon. These results were presented in Figure S4 and Table S4 and discussed in the Results (Lines 198-206).
Furthermore, we defined each of the homoeologs or homoeolog groups in B. hybridum as follows. Homoeolog groups: genes on Bd and Bs sub-genome in B. hybridum, Homoeologs: each gene in each of the subgenomes in B. hybridum.

Minor comments
Introduction
1. The introduction would take advantage of clear hypotheses regarding the evolution of global gene and homoeo-specific expression in B. hybridum.

We revised the introduction to explain the hypotheses regarding the evolution of transcriptome in B. hybridum as follows: “The broader growth area of B. hybridum compared to its ancestors led us to the hypothesis that its duplicated genes may have contributed to its adaptive and ecological fitness through gene expression changes following allotetraploidization. Specifically, in the B. hybridum transcriptome, each of the homoeologs may show particular expression patterns in response to abiotic stimuli, which are associated with the ancestral growth habitats and gene expression.” (Lines 112-116).

Powered by Editorial Manager® and ProduXion Manager® from Aries Systems Corporation

Methods

2. It is not clear how the MAV was calculated.

We revised the sentences describing the calculation of MAV as follows: “As the MAV, which is the average of the expression values in both ancestors, the total read count data from the B. distachyon and B. stacei reads were used” (Lines 427-428).

3. Could the authors provide more details on the trimming step. For both DNA and RNA-Seq, it is also unclear how the coverage was calculated and what were the parameters used to detect the SNPs (minimum read coverage and number of reads).

We added additional information about the trimming and the SNP calling steps (Lines 380-381, 394-396, and 416-417).

4. Line 307-308: What were the identity thresholds used to divide the three groups?

We described the identity thresholds used to divide the RNA-Seq reads from B. hybridum into the three groups as follows: “The expression levels of the homoeologs in B. hybridum were quantified by classifying the RNA-Seq reads into the following three groups based on the number of mismatches between read and both genomes using original Perl script (Additional file 5): Bd-subgenome origin reads, Bs-genome origin reads, and unclassified reads (Additional file 1; Figure S5B).” (Lines 421-424).

5. Line 318: Differentially and not deferentially

We revised the term.

6. DESeq2: Did the authors perform a filtration step of the least expressed data?

For the filtration step, we used genes that showed significant expression with the RPM values more than 1 (Lines 431-432 and 451).

7. GO enrichment: What was the reference set of genes used in the GO term enrichment analysis?

We added sentences that explain the reference set of genes used in the GO term enrichment analysis in the Methods section (Lines 465-466).

8. Line 308: Genome instead of gnome.

We revised the typographical error.

Results

9. Lines 172-188: Additionally to the proportions of genes (or homoeologues) differentially expressed, would be useful to add the number of genes altered.

We added the data to explain the proportions of homoeolog groups showing non-additive and non-ancestral expressions in B. hybridum in Lines 230, 234, 237, and 241. We also listed the numbers and proportions of genes and homoeologs differentially expressed in the revised Figure 3.

10. Lines 201 to 209: Sentences are not clear.

We revised the sentences for clarity as follows: “We then dissected the expression patterns of the 3,725 homoeolog groups from B. hybridum into Bd- and Bs-homoeologs, and found that 2,088 homoeolog groups (56% of the 3,725 homoeolog groups) were preferentially expressed by the Bs-homoeologs (Figure 4B). The distribution of fold-change expression values between Bd- and Bs-homoeologs in all expressed homoeolog groups in B. hybridum under heat stress for 3 days indicated that most of the homoeologs were evenly expressed by both subgenomes (Additional file 1: Figure S7A), whereas the 3,725 homoeolog groups showed abundant expression in the Bs-homoeologs (Additional file 1: Figure S7B). Additionally, of the 2,088 Bs-homoeologs, 1,791 genes were shared with the 5,649 genes that showed higher expression in B. stacei than in B. distachyon; these genes included those

Powered by Editorial Manager® and ProduXion Manager® from Aries Systems Corporation

specifically involved in metabolic processes as well as cellular response to stress and damage stimulus (Figures 4C and D).” (Lines 279-289).

11. Figure 6 is unclear, needs more explanations in the caption and the manuscript.

We revised the part in the manuscript related to Figure 6 (Lines 360-363).

Reviewer #2
Major comments
1. One of the main observations in the study is that "B. distachyon was transcriptionally insensitive to heat after 3 days of exposure to heat stress…" (pg.11, ln. 190), while B. stacei and B. hybridum are responsive. And, this lead to the conclusion that B. stacei and B. hybridium may be better acclimated to heat stress, through the early activation of heat stress responsive genes. This conclusion was primarily based on a Pearson correlation analyses of the global expression changes among the three species under stressed vs. unstressed conditions (Fig. 4A). I am skeptical about this conclusion---given that other published studies show that B. distachyon transcriptome is highly responsive to heat stress, as early as 10-24 h, and >1000 genes are differentially expressed (Priest et al., 2014, PLoS One, 9(1): e87499). It would be highly implausible that there would be no quantitative or qualitative differences in gene expression profiles in B. distachyon, even after 3 days of heat stress. And, if there are some changes in gene expression, it cannot be concluded that B. distachyon transcriptome is 'transcriptionally insensitive".

We appreciate the comments and agree with the suggestions. We revised the sentences as follows: “B. distachyon transcriptomes were not significantly different between normal (22°C) and heat stress (32°C) conditions after 3 days of exposure to heat stress, whereas the B. hybridum transcriptome was noticeably altered under the heat stress as compared to the normal condition; this might reflect physiological traits inherited from B. stacei. When gene expression patterns of B. distachyon, B. stacei, and B. hybridum, as well as the Bd- and Bs-homoeologs of B. hybridum after 3 days of exposure to heat stress were compared, we found that the transcriptomes of B. stacei and B. hybridum showed lower correlations between normal and heat stress conditions than those of B. distachyon (Pearson’s correlation coefficient (PCC) 0.68–0.75, 0.87, and 0.92–0.95 in B. stacei, B. hybridum, and B. distachyon, respectively; Figure 4A), suggesting that B. stacei and B. hybridum respond more strongly to the heat stress than B. distachyon.” (Lines 269-277).

2-1. B. stacei and B. distachyon genomes are publicly available at Phytozome (available since 2015). It would have been easier and appropriate to compare and identify homeologs of B. hybridium by mapping the reads to the parental genomes, rather re-sequencing and constructing a virtual B. stacei genome based on SNPs.

We are aware that B. stacei genome sequence is available at Phytozome. However, according to the policy issued by JGI, we are not allowed to publish any articles containing the analyses of genes or genomic data on a whole genome or chromosome scale using the B. stacei genome sequence prior to publication by JGI and/or its collaborators of a comprehensive genome analysis (https://phytozome.jgi.doe.gov/pz/portal.html#). Therefore, we identified genome-wide homoeologous SNPs using our own sequencing data of B. stacei and B. hybridum and constructed a virtual B. stacei genome. To verify the results of our homoeolog specific gene expression analysis, we calculated the Pearson’s correlation coefficient between the expression values of each homoeolog calculated by using our virtual B. stacei genome and the B. stacei genome available at Phytozome. The Pearson’s correlation coefficient was more than 0.97, suggesting that our homoeolog specific gene expression analysis based on our virtual B. stacei genome was comparable to that of the B. stacei genome.

2-2. Besides, for the SNP calling, the authors utilized SAMtools. However, for polyploid genomes, SAMtools is not appropriate, as it is designed for diploid species. The authors should have used tools like GATK, FreeBayes that are superior and better for polyploid SNP calling. Further, the methods and parameters used for SNP calling are

Powered by Editorial Manager® and ProduXion Manager® from Aries Systems Corporation

not provided in sufficient detail (i.e., coverage, depth etc.). To corroborate these analyses, suggest authors to compare and validate their SNPs/homeologous transcript models with the existing draft B. stacei genome.

We had considered using several tools for SNP calling, including GATK and FreeBayes. However, although B. hybridum is a polyploid species, it is also a self-fertilizing species (Catalán et al., 2012 and 2016) and its homoeologous genomes are homozygous. Therefore, we decided to use samtools and VarScan programs to call SNPs between mapped reads of B. stacei and B. hybridum and the reference genome sequence of the B. distachyon Bd21.

3. It's not clear why the authors did not provide details of interesting differentially regulated homeologous genes, and their respective expression fold-changes. They did mention four genes in the supplemental Table S8, but even those are described without any mention of the fold-change cutoffs. So, what is the fold-change cutoff used in the entire study for the various comparisons. Also, the authors use GO term descriptions to predict overall pathways enriched among the differentially expressed homeologs. The GO terms are good starting points, but can be broad and the enriched categories need to be filtered to remove redundant/overlapping terms.

Following your suggestions, we mentioned the genes in Table S8 (Table S13 in the revised manuscript) as follows “The comprehensive list of Bs-homoeolog and B. stacei genes with significantly higher expression than the B. distachyon genes after 3-day exposure to 32°C included genes involved in the regulation of acclimation to heat in plants, such as heat shock transcription factor (Hsf), heat shock protein (Hsp), and DNAJ [52-55] (1,791 genes in Figure 4C, Additional file 2: Table S13).”(Lines 355-358). Moreover, we also noted that we used FDR as a threshold to identify DEGs in the Methods section (Line 451). Finally, we summarized the enriched GO terms using REVIGO (http://revigo.irb.hr/) and added this information in the Methods section (Lines 466-467).

4. The narrative corresponding to the results presented in Fig. 3 need to be elaborated. In this section, the authors write that "the homoeologous transcripts were almost evenly expressed between the B. distachyon and B. stacei genomes in the B. hybridum transcriptome of the leaf and root tissues under normal conditions" (Pg.10-11, ln 177-179), but at the same time conclude that "~26% and ~38% of the homoeologous gene pairs in B. hybridum changed its expression pattern towards non-additive expression and non-parental expression" (Pg.11, ln 181). It's not clear to me what can be concluded from these statements, and how the specific analysis pertaining to Fig. 3 was performed.
Authors need to define and describe the methodology for estimation of mid-ancestral values (MAVs), non-additive, non-parental expression terms so that non-evolutionary biologists can also understand this section. Besides this analysis was performed only using unstressed tissues. It would have been more
informative to analyze how these different polyploid gene expression patterns (non-additive or non-parental) change under heat stress.

Following the comment pertaining to the proportion of genes that show non-ancestral expression Figure 3 and Figure S6, we rephrased the related text in the revised manuscript (Lines 249-254 and 263-266). To address the comments about the method used to estimate MAV, we revised the text that describes the method for MAV calculation as follows: “As the MAV, which is the average of the expression values in both ancestors, the total read count data from the B. distachyon and B. stacei reads were used” (Lines 427-428).
Moreover, to facilitate the understanding of gene expression patterns in allopolyploid species such as non-additive expression, we added the possible outcomes of transcriptome in allopolyploidy on gene expression in the Introduction section (Lines 80-104).
As per the comments on our identification of differentially expressed homoeologs in response to heat stress, we identified genes showing non-additive expression under heat stress conditions and listed enriched functions based on their GO annotations. We found that genes involved in biological functions such as response to stimulus and abiotic stimulus were enriched in those genes, which suggests that increased expression divergence of genes related to these functions enhanced their ability to

Powered by Editorial Manager® and ProduXion Manager® from Aries Systems Corporation

	respond to environmental change and adapt to ecological niches. These results were presented in newly added Tables S11 and S12 and discussed in the Discussion (Lines 333-337).<br><br>5. There was no validation of the RNA-seq data by alternate approaches such as RT-qPCR. This is very important QC for any transcriptome-level study. This is even more relevant for this study that relates to homeolog reconstruction and expression differences of homeologous genes. I would suggest validating the expression differences of the homeologs, that can corroborate both the genome-level and RNA-seq analysis.<br><br>As suggested, we validated the expression ratios of homoeologs found in B. hybridum by using the TaqMan SNP Genotyping Assay in combination with a digital PCR system. Specifically, we assessed the quantitative distribution of the decisive SNPs in the two homoeolog groups corresponding to the genes annotated in the Bd21 genome; Bradi1g16510 (APX2-like) and Bradi2g49660 (Hsp101). We found that the proportions of the SNPs in the cDNA amplicons from the B. hybridum RNAs sampled after 3 days of exposure to 32°C were comparable to their expression bias observed in the RNA-seq analysis. These results were presented in a newly added Figure S10 and S11and discussed in the Results (Lines 313-322).
**Additional Information:**	
**Question**	**Response**
Are you submitting this manuscript to a special series or article collection?	No
**Experimental design and statistics**<br><br>Full details of the experimental design and statistical methods used should be given in the Methods section, as detailed in our Minimum Standards Reporting Checklist. Information essential to interpreting the data presented should be made available in the figure legends.<br><br>Have you included all the information requested in your manuscript?	Yes
**Resources**<br><br>A description of all resources used, including antibodies, cell lines, animals and software tools, with enough information to allow them to be uniquely identified, should be included in the Methods section. Authors are strongly encouraged to cite Research Resource Identifiers (RRIDs) for antibodies, model organisms and tools, where possible.<br><br>Have you included the information requested as detailed in our Minimum Standards Reporting Checklist?	Yes
**Availability of data and materials**	Yes

Powered by Editorial Manager® and ProduXion Manager® from Aries Systems Corporation

All datasets and code on which the conclusions of the paper rely must be either included in your submission or deposited in publicly available repositories (where available and ethically appropriate), referencing such data using a unique identifier in the references and in the "Availability of Data and Materials" section of your manuscript.<br><br>Have you have met the above requirement as detailed in our Minimum Standards Reporting Checklist?	

Powered by Editorial Manager® and ProduXion Manager® from Aries Systems Corporation

Manuscript
Click here to download Manuscript Revised_Manuscript.docx

Homoeolog-specific activation for heat acclimation in the allopolyploid grass *Brachypodium hybridum*

Kotaro Takahagi[1,2,3], Komaki Inoue[3], Minami Shimizu[2,3], Yukiko Uehara-Yamaguchi[3], Yoshihiko Onda[2,3] and Keiichi Mochida[1,2,3,4]

[1]Graduate School of Nanobioscience, Yokohama City University, 22-2 Seto, Kanazawa-ku, Yokohama, Kanagawa 236-0027, Japan.

[2]Kihara Institute for Biological Research, Yokohama City University, 641-12 Maioka-cho, Totsuka-ku, Yokohama, Kanagawa 244-0813, Japan.

[3]Cellulose Production Research Team, Biomass Engineering Research Division, RIKEN Center for Sustainable Resource Science, 1-7-22 Suehiro-cho, Tsurumi-ku, Yokohama, Kanagawa 230-0045, Japan.

[4]Institute of Plant Science and Resources, Okayama University, 2-20-1 Chuo, Kurashiki, Okayama 710-0046, Japan.

E-mail addresses

Kotaro Takahagi n155267a@yokohama-cu.ac.jp

Komaki Inoue komaki.inoue@riken.jp

Minami Shimizu minami.shimizu@riken.jp

Yukiko Uehara-Yamaguchi yukiko.uehara@riken.jp

Yoshihiko Onda yoshihiko.onda@riken.jp

Keiichi Mochida keiichi.mochida@riken.jp

Corresponding author

Keiichi Mochida, Cellulose Production Research Team, Biomass Engineering Research Division, RIKEN Center for Sustainable Resource Science, 1-7-22 Suehiro-cho, Tsurumi-ku, Yokohama, Kanagawa 230-0045, Japan. Tel: +81-45-503-9111, E-mail: keiichi.mochida@riken.jp

Abstract

Background: Allopolyploid plants often show wider environmental tolerances than their ancestors; this is expected to be due to the merger of multiple distinct genomes with a fixed heterozygosity. The complex homoeologous gene expression could have been evolutionarily advantageous for the adaptation of allopolyploid plants. Despite multiple previous studies reporting homoeolog-specific gene expression in allopolyploid species, there are no clear examples of homoeolog-specific function in acclimation to a long-term stress condition.

Results: We found that the allopolyploid grass *Brachypodium hybridum* and its ancestor *Brachypodium stacei* show long-term heat stress tolerance, unlike its other ancestor, *Brachypodium distachyon*. To understand the physiological traits of *B. hybridum*, we compared the transcriptome of the three *Brachypodium* species grown under normal and heat stress conditions. We found that the expression patterns of ~26% and ~38% of the homoeolog groups in *B. hybridum* changed towards non-additive expression and non-ancestral expression, respectively, under normal condition. Moreover, we found that *B. distachyon* showed similar expression patterns between normal and heat stress conditions, whereas *B. hybridum* and *B. stacei* significantly altered their transcriptome in response to heat after 3 days of stress exposure, and homoeologs that were inherited from *B. stacei* may have contributed to the transcriptional stress response to heat in *B. hybridum*. After 15 days of heat exposure, *B. hybridum* and *B. stacei* maintained transcriptional states similar to those under normal conditions. These results suggested that an earlier response to heat that was specific to

homoeologs originating from *B. stacei* contributed to cellular homeostasis under long-term heat stress in *B. hybridum*.

Conclusions: Our results provide insights into different regulatory events of the homoeo-transcriptome that are associated with stress acclimation in allopolyploid plants.

Keywords

Abiotic stress response; Allopolyploidy; *Brachypodium hybridum*; Heat acclimation; Homoeolog; Hybrid species; Transcriptome

Background

Polyploidy is a common phenomenon in eukaryotes, especially in plants [1-4], and is recognized as a fundamental mechanism in plant evolution and diversification [5, 6]. It has been suggested that all angiosperms have experienced one or more polyploidization events during their evolutionary history [7-10]. Recent evolutionary genomic studies have suggested that genome duplication events occurred widely in plants at the Cretaceous–Paleogene boundary, which is a major extinction event in the earth's history. Evolutionary views represent a hypothesis that plants with duplicated genomes might have a better chance for survival under global adverse conditions [11, 12].

Interspecific hybridization and subsequent genome duplication led to evolutional changes in hybrid

species that represented fixed heterozygosity. Allopolyploid plants generally show better growth vigor and stress tolerance than their ancestors [13-15]. For example, relative to their ancestors, allopolyploid *Arabidopsis* (*Arabidopsis suecica*) shows more vigorous growth, and allopolyploid *Spartina* (*Spartina anglica*) shows better tolerance to reducing conditions and sulfite-rich sediments [16-18]. Both natural and synthetic wheat (*Triticum aestivum*) have higher fitness under salt stress than their diploid and tetraploid ancestors [19, 20]. It has been suggested that allopolyploidization may have contributed to the adaptation to a wide range of environmental conditions [21, 22].

The expression levels of homoeologs in allopolyploid species often show non-additive expression that are deviated from parental additivity comparing to its ancestors [23]. The non-additive expression has been widely observed in various allopolyploid species [24-26] , and can be explained by at least three possible scenarios; total gene expression of a homoeolog group in an allopolyploid species is similar to that of one of its parental species (expression level dominance (ELD)), total gene expression is lower or higher than in both parents (transgressive expression), and uneven contribution of homoeologs to gene expression (homoeolog expression bias) [23, 27]. Through comparative gene expression analysis of total expression levels of homoeolog groups in an allopolyploid species and “mid-parental values (MPVs)” as the average expression levels of genes in its parents, parental additivity and non-additivity of homoeologs can be examined. By discrimination of homoeologous gene expression, homoeolog expression bias can be identified across tissues, developmental stages, and environmental conditions [28-30].

The contribution of expression plasticity of duplicated genes observed in diverse allopolyploid species to adaptive and ecological fitness has been debated. Comparative transcriptome analysis of the natural allopolyploid *Coffea arabica* and its ancestral species (*Coffea canephora* and *Coffea eugenioides*) revealed evidence of a genomic ELD that depends on growth temperature [31] . Comparative transcriptome analysis of hexaploid *T. aestivum* (AABBDD) and its ancestors (*Triticum turgidum* (AABB) and *Aegilops tauschii* (DD)) demonstrated differential enrichment of over-represented gene functions between the ELD genes of both parents, which suggests they may have differentially contributed to particular biological functions in hexaploid wheat [32]. Comparative gene expression analysis of a synthetic tetraploid wheat and its parental species showed transgressive expression of genes that are involved in particular biological functions such as transport, modification, and uroporphyrinogen decarboxylase activity, suggesting that transgressive gene expression may have rapidly occurred following allopolyploidization [33] . Studies on the allotetraploid *Arabidopsis kamchatica* and its ancestral species demonstrated homoeolog expression bias in genes related to adaptation to cold and heavy metal environments, which were inherited from the respective ancestors that are tolerant against cold and heavy metal stresses, respectively [34, 35].

Brachypodium hybridum (2n=30) is a natural allopolyploid that is derived from a cross between *Brachypodium distachyon* (2n=10) and *Brachypodium stacei* (2n=20) that occurred approximately 1 million years ago (MYA; Additional file 1: Figure S1) [36-43]. Although these species inhabit a circum-Mediterranean region, their environmental niches are clearly different. *B. distachyon* grows in higher-altitude,

cooler, and wetter areas, whereas *B. stacei* is found in lower-altitude, warmer, and drier areas. The hybrid species *B. hybridum* grows in areas that are overlapping as well as specific to its ancestors; this growth pattern suggests that its speciation is associated with particular environmental conditions and adaptations to diverse environmental conditions [37]. The broader growth area of *B. hybridum* compared to its ancestors led us to the hypothesis that its duplicated genes may have contributed to its adaptive and ecological fitness through gene expression changes following allotetraploidization. Specifically, in the *B. hybridum* transcriptome, each of the homoeologs may show particular expression patterns in response to abiotic stimuli, which are associated with the ancestral growth habitats and gene expression. Therefore, this trio of species has been recently proposed as a model for grass speciation via adaptation and polyploidization [39, 40, 43].

In the current study, we conducted a comparative global transcriptome analysis of *B. hybridum* and its ancestors. The analysis revealed non-additive transcriptome changes in the leaf and root tissues of *B. hybridum*. Additionally, we performed a homoeolog-specific transcriptome analysis by discriminating RNA-sequencing (RNA-Seq) reads of each homoeolog of *B. hybridum*, and determined the non-ancestral gene expression patterns. Finally, we assessed homoeolog-specific transcriptome changes in response to heat stress in *B. hybridum* and we discuss the differential regulation of the homoeo-transcriptome that is associated with heat stress tolerance in *B. hybridum* inherited from *B. stacei*.

Data Description

Plant materials

Three *Brachypodium* species were used in this study: the allotetraploid *B. hybridum* Bd14-1 and the diploid ancestors *B. distachyon* Bd21 and *B. stacei* ABR114. The accessions of these species were provided by the National Plant Germplasm System of USDA-ARS, David F. Garvin (USDA-ARS Plant Science Research Unit, University of Minnesota, USA), and Pilar Catalán (Department of Agriculture and Environment Science, High Polytechnic School of Huesca, University of Zaragoza, Spain). Dry seeds were incubated on wet filter paper in a Petri dish at 4°C in the dark for 6–7 days to synchronize germination. The germinated seeds were grown in a growth chamber at 25°C under a 16-h day photoperiod (60 $\mu mol \cdot m^{-2} \cdot s^{-1}$) for 4 days. The plants were transplanted to pots filled with autoclaved PRO-MIX BX MYCORRHIZAE (Premier Tech, Quebec, Canada). The potted plants were grown in a growth chamber at 22°C (normal condition) or 32°C (heat stress condition) under a 20-h day photoperiod (100 $\mu mol \cdot m^{-2} \cdot s^{-1}$), and watered with 5,000-fold diluted Professional Hyponex 10-30-20 (Hyponex Japan, Osaka, Japan) every three or four days.

Whole genome sequence data

Genomic DNA from the leaf tissues of *B. hybridum* and *B. stacei* were extracted using the DNeasy Plant Mini Kit (Qiagen, Tokyo, JAPAN). Libraries for single-end DNA sequencing were obtained using the Ion Xpress™ Plus Fragment Library Kits (Life Technologies Japan, Tokyo, Japan), and semiconductor chips that

were used for sequencing were prepared using Ion OneTouch 2 System (Life Technologies Japan) and Ion P1™ Chip v2 (Life Technologies Japan). The sequencing analyses were performed using an Ion Proton sequencer (Life Technologies Japan). The genome sequence data of *B. hybridum* and *B. stacei* were archived at DDBJ under the accession number DRA005717 (Additional file 2: Table S1).

RNA sequence data

Shoots and roots from each species grown in a growth chamber for 4 days after synchronized germination were sampled to elucidate the global homoeolog expression patterns in *B. hybridum*. Shoots and most young leaf blades from each species grown in a growth chamber under different temperature conditions (22°, normal condition, and 32°C, heat stress condition) for 3 and 15 days, respectively, after being transplanted were sampled to elucidate the homoeolog-specific transcriptional response to heat stress in *B. hybridum*. Total RNA was extracted from each sample using the RNeasy plant mini kit (Qiagen). Poly(A) RNAs were purified using the NEBNext® Poly(A) mRNA Magnetic Isolation Module (New England Biolabs, MA, USA). The libraries for single-end strand-specific RNA sequencing were obtained using the Ion Total RNA-Seq Kit v.2 (Life Technologies Japan). Size-selected libraries were purified using Agencourt AMPure XP (Beckman Coulter, CA, USA). Semiconductor chips that were used for sequencing were prepared using the Ion PI™ Hi-Q™ OT2 200 Kit (Life Technologies Japan), Ion PI™ Hi-Q™ Sequencing 200 Kit (Life Technologies Japan), and Ion P1™ Chip v3 (Life Technologies). The sequencing analyses were performed using an Ion

Proton sequencer (Life Technologies Japan) with three biological replicates. The RNA-sequencing data were archived at DDBJ under the accession number DRA005699 (Additional file 2: Table S1).

Results

B. hybridum and _B. stacei_ show significant tolerance to long-term heat stress

Unlike *B. distachyon*, *B. hybridum* and *B. stacei* showed significant tolerance to long-term heat stress. The allopolyploid *B. hybridum* and its ancestor *B. stacei* grow in warmer regions, in contrast to the other ancestor *B. distachyon*, which suggests that *B. hybridum* and *B. stacei*, but not *B. distachyon*, might have adapted to high-temperature conditions. To test this hypothesis, we compared the plant biomasses of *B. hybridum*, *B. stacei*, and *B. distachyon* grown under normal (22°C) and heat stress (32°C) conditions at two time points (after 3 and 15 days of heat stress exposure). After 3 days of exposure to heat stress, the three species showed no differences in growth (Figure 1A). Conversely, after 15 days of heat exposure, *B. distachyon* showed a significant decrease in fresh weight ($P < 0.01$, *t*-test), whereas *B. hybridum* and *B. stacei* maintained their growth (Figure 1B). This result indicated that *B. hybridum* and *B. stacei* are thermotolerant species and that *B. hybridum* might have inherited this trait from *B. stacei* via allopolyploidization.

A virtual _B. stacei_ genome generated from the comparative analysis of homoeologous genomes

By comparing the genomes of *B. hybridum*, *B. stacei*, and *B. distachyon*, we obtained a comprehensive map

of the homoeologous single nucleotide polymorphisms (SNPs) in these *Brachypodium* species. This map enabled the distinction of transcripts expressed from each of the homoeologs in *B. hybridum*. To determine polymorphisms between the homoeologous genomes in *B. hybridum*, we sequenced the genomes of *B. stacei* ABR114 and *B. hybridum* Bd14-1 and mapped the reads to the reference genome sequence of *B. distachyon* Bd21. We found that 85% and 91% of the genomic reads of *B. stacei* and *B. hybridum* mapped to the reference genome and covered 89% and 98% of the genic region of the reference genome, respectively, which suggests high similarity among the homoeologous genomes (Figure 2 and Additional file 2: Table S2). As for genomic polymorphisms, 11,948,285 SNPs were identified between *B. distachyon* and *B. stacei* and 10,216,010 SNPs between *B. distachyon* and *B. hybridum* (Additional file 2: Table S3). We selected 5,720,539 SNPs to discriminate the homoeologs that were homogenic to the *B. stacei* reads from those that were heterogenic to the *B. hybridum* reads (Figure 2, Additional file 1: Figure S2 and Additional file 3). By replacing the nucleotides of the homoeologous SNPs in the *B. distachyon* genome with those in *B. stacei*, we generated virtual homoeolog sequences of *B. stacei* that corresponded to *B. distachyon* counterparts (Additional file 1: Figure S3).

Genome-wide polymorphism between *B. distachyon* and *B. stacei* genomes

Through a comprehensive identification of homoeologous SNPs between *B. distachyon* and *B. stacei* genomes, we assessed the distribution of the SNPs within various genomic regions to gain insights into the

genetic diversity of the two genomes. We found that 22.8% of homoeologous SNPs are distributed within the intergenic regions, and the remaining 77.2% of the SNPs were identified in the genic regions (Additional file 1: Figure S4A). Specifically, 45.4% and 17.6% of SNPs are distributed in intronic and exonic regions, respectively, and 12.5% and 1.6% of SNPs are distributed in UTRs and splice sites, respectively. We assessed the types of polymorphism found in genic regions, and found that 26,986 genes (79% of the annotated genes in the Bd21 genome) contain nonsynonymous variations. Of these, 4,558 genes (13% of the annotated genes in the Bd21 genome) contain several variations in the *B. stacei* genome; 683, 2,980, and 895 genes show start codon loss, stop codon gain, and stop codon loss, respectively (Additional File 1: Figure S4B and Additional File 2: Table S4). Based on the synonymous substitution rate (*Ks*) between the homoeologs, we estimated a divergence time of approximately 6.4 MYA between *B. distachyon* and *B. stacei*, which is accordance with the divergence time of *B. stacei* of 5.8–16.4 MYA previously estimated by Catalan et al. 2012 [38]. Moreover, we computed the non-synonymous substitution rate (*Ka*) and *Ka*/*Ks* ratio, and identified 1,714 homoeologs whose *Ka*/*Ks* values were greater than 1, supporting their accelerated evolution. Interestingly, we found that genes involved in DNA mismatch repair with Gene Ontology (GO) terms such as proliferating cell nuclear antigen complex and DNA polymerase processivity factor activity (GO:0043626 and GO:0030337, respectively, *P*-value 4,8e-04) are significantly enriched in those genes, which suggested that DNA damage tolerance may reflect selection and adaptive divergence between *B. distachyon* and *B. stacei*.

Non-additive gene expression in *B. hybridum*

To identify gene expression changes in *B. hybridum* as a result of the tetraploidization, we examined expression levels of its homoeolog groups in comparison to mid-ancestral values (MAVs) of those estimated from each of the counterpart homoeologs in its ancestors, *B. distachyon* and *B. stacei*, and identified the homoeolog groups that showed non-additive expression in the leaf and root transcriptomes of *B. hybridum*. To this end, we performed RNA-Seq-based transcriptome analysis of leaf and root mRNA samples of *B. distachyon*, *B. stacei*, and *B. hybridum*, and quantified gene expression of the homoeologs and their counterparts in the ancestors by mapping the RNA-seq reads to the Bd21 genome (*B. distachyon* reads), the virtual *B. stacei* genome (*B. stacei* reads), or these both genomes (*B. hybridum* reads) (Additional file 1: Figure S5A). Through comparisons of the MAV estimated from the ancestors and expression level of the homoeolog groups in *B. hybridum*, we identified 17,256 and 18,021 (50% and 53% of the annotated genes in the Bd21 genome) of genes and homoeolog groups are expressed (supported by MAV $\geq$ 1 and RPM $\geq$ 1 in *B. hybridum*) in the root and leaf transcriptomes, respectively (Figures 3A and B). Then, we classified these expressed homoeolog groups according to additive or non-additive expression, and found that 3,994 and 4,681 homoeolog groups (23% and 26% of the analyzed homoeolog groups in leaf and root tissues, respectively) were non-additively expressed in the *B. hybridum* leaf and root transcriptomes, respectively (the remaining 13,262 and 13,340 homoeolog groups in leaf and root tissues, respectively, were additively expressed; Figures 3C and D). Moreover, we assessed ELD in the non-additively expressed homoeolog

groups. We found that 611 and 668 homoeolog groups in leaf and root tissues, respectively (3.5% and 3.7% of the analyzed homoeolog groups) in *B. hybridum* showed ELD, with expression levels similar to those in *B. distachyon* (ELD-Bd) (Figures 3C and D). On the other hand, 1,263 and 1,283 homoeolog groups in leaf and root tissues, respectively (7.3% and 7.1% of analyzed homoeolog groups) in *B. hybridum* showed ELD of *B. stacei* (ELD-Bs) (Figures 3C and D). These findings indicated that the number of homoeolog groups of ELD-Bs is nearly twice that of ELD-Bd, suggesting that gene expression patterns of *B. stacei* are preferentially inherited to the *B. hybridum* transcriptome in leaf and root tissues. We also identified 627 and 850 homoeolog groups in *B. hybridum* leaf and root tissues, respectively (3.6% and 4.7% of the analyzed homoeolog groups), that showed transgressive expression, i.e., higher expression than in both ancestors. Various GO terms, such as primary metabolic processes, primary cellular processes, stress response, and protein transport, localization, and translation were enriched in the set of homoeolog groups that showed transgressive expression (Additional file 2: Tables S5 and S6), suggesting that the overproduction of transcripts may affect various biological functions through the tetraploidization.

Homoeolog expression bias in *B. hybridum*

To identify homoeolog expression bias in *B. hybridum*, we sorted the *B. hybridum* RNA-Seq reads to the two homoeologous genomes of *B. distachyon* (Bd-subgenome) and *B. stacei* (Bs-subgenome) based on their sequence identities after alignments with both genomes, and quantified the expression levels of each of

homoeologs; Bd- and Bs-homoeologs in the *B. hybridum* transcriptome. Through the homoeolog-specific RNA-Seq read sorting, we discriminated 75–79% of the RNA-seq reads between the Bd-subgenome origin reads and Bs-subgenome origin reads (Additional file 1: Figures S5 and S6). We found that 6,512 and 6,296 homoeolog groups show non-ancestral expression patterns in the *B. hybridum* leaf and root transcriptomes, respectively (38% and 35% of the analyzed homoeolog groups; orange, light blue, and purple boxes in Figures 3E and 3F), and the remaining 10,744 and 11,725 retained the ancestral expression patterns (grey and red boxes in Figures 3E and 3F). In the homoeolog groups showing ancestral expression patterns, we found that similar proportions of the homoeolog groups retained the expression bias of each of the ancestral transcriptome (27% of the analyzed homoeolog groups in leaf and root tissues; red boxes in Figures 3E and 3F). In the homoeolog groups showing non-ancestral expression patterns, we observed bias loss in most cases in the *B. hybridum* transcriptomes (25% and 23% of the analyzed homoeolog groups in leaf and root tissues, respectively; light blue boxes in Figures 3E and 3F). These findings suggested that more than 30% of the homoeologous gene pairs in *B. hybridum* had changed their expression pattern towards non-ancestral expression during speciation over more than 1 MYA after the genome-scale gene duplication via allopolyploidization.

Homoeolog-specific transcriptional response to heat stress in *B. hybridum*

B. distachyon transcriptomes were not significantly different between normal (22°C) and heat stress (32°C)

conditions after 3 days of exposure to heat stress, whereas the *B. hybridum* transcriptome was noticeably altered under the heat stress as compared to the normal condition; this might reflect physiological traits inherited from *B. stacei*. When gene expression patterns of *B. distachyon*, *B. stacei*, and *B. hybridum*, as well as the Bd- and Bs-homoeologs of *B. hybridum* after 3 days of exposure to heat stress were compared, we found that the transcriptomes of *B. stacei* and *B. hybridum* showed lower correlations between normal and heat stress conditions than those of *B. distachyon* (Pearson's correlation coefficient (PCC) 0.68–0.75, 0.87, and 0.92–0.95 in *B. stacei*, *B. hybridum*, and *B. distachyon*, respectively; Figure 4A), suggesting that *B. stacei* and *B. hybridum* respond more strongly to the heat stress than *B. distachyon.* We also found that, under after 3 days of stress exposure, 5,649 genes and 3,725 homoeolog groups showed significantly higher expression in *B. stacei* and *B. hybridum*, respectively, than in *B. distachyon* (false discovery rate (FDR) ≤ 0.001). We then dissected the expression patterns of the 3,725 homoeolog groups from *B. hybridum* into Bd- and Bs-homoeologs, and found that 2,088 homoeolog groups (56% of the 3,725 homoeolog groups) were preferentially expressed by the Bs-homoeologs (Figure 4B). The distribution of fold-change expression values between Bd- and Bs-homoeologs in all expressed homoeolog groups in *B. hybridum* under heat stress for 3 days indicated that most of the homoeologs were evenly expressed by both subgenomes (Additional file 1: Figure S7A), whereas the 3,725 homoeolog groups showed abundant expression in the Bs-homoeologs (Additional file 1: Figure S7B). Additionally, of the 2,088 Bs-homoeologs, 1,791 genes were shared with the 5,649 genes that showed higher expression in *B. stacei* than in *B. distachyon*; these genes included those

specifically involved in metabolic processes as well as cellular response to stress and damage stimulus (Figures 4C and D). These results suggested that the functions of *B. hybridum* genes that were inherited from *B. stacei* may have contributed to the transcriptional stress response and associated metabolic changes in the *B. hybridum* transcriptome during the earlier response to heat stress.

Early transcriptional responses of Bs-homoeologs contribute to the maintenance of cellular functions in *B. hybridum* under long-term heat stress

After 15 days of exposure to heat stress, *B. hybridum* and *B. stacei* maintained their transcriptional states similar to those under normal condition, in contrast to the severely damaged cellular system of *B. distachyon*. When we compared the expression patterns between *B. distachyon*, *B. stacei*, and *B. hybridum* after 15 days of exposure to heat stress, we found that *B. stacei* and *B. hybridum* showed similar expression patterns between stress and normal conditions (PCC 0.94–0.96 and 0.92–0.96, respectively), whereas *B. distachyon* showed remarkable changes in its transcriptome (PCC 0.84–0.89) reflecting the severe decrease in its biomass (Figures 1B and 5A). Specifically, genes involved in primary metabolism, such as photosynthesis and metabolite and energy generation, were significantly less represented in *B. distachyon* than in *B. stacei* and in *B. hybridum* under long-term heat stress (Figures 5B and C and Additional file 2: Tables S7 and S8), which likely indicates the physiological sensitivity of its cellular system to heat stress. The number of Bs-homoeologs that were expressed more abundantly than Bd-homoeologs was reduced in homoeolog groups

that were highly expressed in *B. hybridum* than in *B. distachyon* at 15 days after exposure to heat stress compared to that found after 3 days of heat stress exposure (Figure 4B and Additional file 1: Figure S8), which suggests that Bs-homoeologs are significantly activated at the earlier phase of transcriptional response to heat stress. We also found significantly higher expression of Bs-homoeologs and genes in *B. stacei* encoding A2-type heat shock transcription factor (*HsfA2*) as well as putative *HsfA2*-targeted genes such as heat shock protein 101 (*Hsp101*) and ascorbate peroxidase 2-like (*APX2*-like; homologs of *Arabidopsis APX1* and rice *APX2*), which are known as key factors in response to heat [44-46], comparing to expression of their counterparts in *B. distachyon* at 3 days after exposure to 32°C (Additional file 1: Figure S9). To validate the homoeolog-specific gene expression in *B. hybridum* observed in our RNA-Seq data, we quantified the polymorphic nucleotides in the cDNA amplicons of *B. hybridum* by using a TaqMan SNP Genotyping Assay in combination with a digital PCR system. The assay discriminates between two homoeo-alleles of a specific SNP that are labeled with two fluorescent dyes (FAM and VIC) and quantifies the expression of each allele by detecting the fluorescence. Specifically, we assessed the quantitative distribution of the decisive SNPs in the two homoeolog groups corresponding to the genes annotated in the Bd21 genome; Bradi1g16510 (*APX2*-like) and Bradi2g49660 (*Hsp101*). We found that the proportions of the SNPs in the cDNA amplicons from the *B. hybridum* RNAs sampled after 3 days of exposure to 32°C were comparable to their expression bias observed in the RNA-seq analysis (Additional file 1: Figures S10 and S11). These results suggested that the earlier response to heat stress that was specific to the Bs-homoeologs likely contributed to the maintenance

of their cellular homeostasis under the long heat exposure.

Discussion

Evolutionary non-additive gene expression in the hybrid grass *B. hybridum* likely underlies its enhanced invasive behavior

Our transcriptome analysis provided comprehensive evidence for non-additive gene expression in the hybrid species *B. hybridum*, suggesting the global transcriptional changes in its leaves and roots evolved through allopolyploidization. The GO analysis of non-additively expressed homoeolog groups in leaf and root tissues of *B. hybridum* relative to both ancestral species showed an overrepresentation of genes that were involved in the response to stimulus and abiotic stimulus (Additional file 2: Tables S9 and S10). Such functions were also enriched in the homoeolog groups in leaf tissues of *B. hybridum* that showed non-additive expression under heat stress condition (Additional file 2: Tables S11 and S12), which suggests that increased expression divergence of genes related to such functions enhanced this species' ability to respond to environmental change and to adapt to ecological niches. Increased expression divergence of these classes of genes has also been reported in allotetraploid *Arabidopsis* and wheat [24-26]. Although such examples have been reported in few species, future progress in transcriptome datasets of hybrid species and their ancestors might enable the application of universal rules for determining transcriptional changes when new hybrid species are generated. Allopolyploid species have long been hypothesized to possess greater environmental adaptability

to wider niches than their ancestors. Specifically, enhanced heterozygosity and genetic diversity resulting from the hybridization of multiple diverged genomes have been thought to upgrade stress tolerances and contribute to the expansion of niches in hybrid species [47-49]. Although *B. hybridum* shows the largest niche overlap compared with its diploid ancestral species, it shows a niche breadth that is smaller than that of *B. distachyon* and slightly greater than that of *B. stacei* [37, 38]. However, *B. hybridum* also successfully colonized non-native regions of the world [37], which suggests it has greater ecological tolerance than other diploids. With the non-additive transcriptional changes in *B. hybridum*, the expanded diversity of gene expression might contribute to the colonization of non-native areas while avoiding inbreeding depression, and might boost its diversifying selection [50, 51].

Homoeolog-specific gene expression underlies acclimation to heat stress

Although no differences in visible traits were found between the three *Brachypodium* species after 3 days of exposure to heat stress, significant differences in physiological traits in the early stage of heat exposure were revealed from their transcriptomes. The comprehensive list of Bs-homoeolog and *B. stacei* genes with significantly higher expression than the *B. distachyon* genes after 3-day exposure to 32°C included genes involved in the regulation of acclimation to heat in plants, such as heat shock transcription factor (Hsf), heat shock protein (Hsp), and DNAJ [52-55] (1,791 genes in Figure 4C, Additional file 2: Table S13), Thus, the functions of *B. hybridum* in acclimating to heat are likely specifically inherited from *B. stacei* via

allopolyploidization. Based on our transcriptome analysis using the *Brachypodium* trio, we hypothesize that the transcriptional response of Bs-homoeologs in *B. hybridum* and their counterparts in *B. stacei* at the early phase during heat exposure contribute to the maintenance of their physiological equilibrium under long-term heat exposure, and might presumably be associated with their heat stress tolerance (Figure 6). Previous evolutional and ecological studies suggested that *B. distachyon* could have adapted to different environments by diverging from *B. stacei* [37, 38]. The distribution areas of *B. distachyon* and *B. stacei* suggested that the two *Brachypodium* species have adapted to cooler and wetter areas and to warmer and drier areas, respectively, via diversification [37]. Therefore, the heat acclimation function in *B. distachyon* might have been lost during its adaptation process after branching from the common ancestor of these diploids. The heat-adaptive trait in the *B. stacei* genome could influence the survival of both individual plants and hybrid progeny under heat stress conditions.

Methods

Fresh weight measurement

The aboveground parts of plants of each species grown under normal and heat stress conditions for 3 or 15 days were used to measure fresh weight. Twelve individuals were used for the measurement. A *t*-test was used for statistical comparisons between the plants grown under the different conditions. The significance threshold was set at $P < 0.01$.

Coverage calculation and SNP calling

To obtain high-quality reads, the genome sequence reads were trimmed by cutting bases off from the start and end of reads if quality ≤20, and removing the reads with final read length <50 using Trimmomatic (v.0.32) [56] with the -thread 2 LEADING: 20 TRAILING: 20 MINLEN: 50 commands. The trimmed reads were mapped to the reference genome sequence of Bd21 downloaded from Phytozome (Bdistachyon_314_v3.0.fa.gz, http://genome.jgi.doe.gov/pages/dynamicOrganismDownload.jsf?organism=Bdistachyon) using TMAP (v.3.1.4; Life Technologies Japan, Tokyo, Japan) with mapall -n 4 -v -Y -u -o 2 stage1 map4 commands. The coverage of genome sequence reads on the reference genome was calculated by removing the non-mapped reads and merging multiple mapping data of the same species from the raw mapping data using SAMTools (v.0.1.19) [57] with the view -F 4 and merge commands. The merged data were subjected to coverage calculation using the genomeCoverageBed in BEDTools (v.2.20.1) [58] with the default setting. SNPs between the genome sequence reads and reference genome were called by removing the non-mapped reads and possible duplicate reads and merging the multiple mapping data of the same species from the raw mapping data using SAMTools with the view -F 4, rmdup, and merge commands. The merged data were subjected to SNP calling using VarScan pileup2snp (v.2.3.7) [59] with the default settings (minimum read depth at a position to make a call is 8 and minimum supporting reads at a position to call variants is 2), except

–p-value 0.01 commands.

Homoeologous SNP identification and virtual *B. stacei* genome construction

Common homogenic SNPs between *B. distachyon* and *B. stacei* and heterogenic SNPs between *B. distachyon* and *B. hybridum* were identified as homoeologous SNPs (Additional file 3); these were used to identify homoeologous genomes in *B. hybridum*. The homoeolog-specific SNPs and their impact to the transcripts and deduced protein sequences were predicted using SNPEff (v.4.2) [60] with the gene structural annotation of Bd21 retrieved from Phytozome (Bdistachyon_314_v3.1.gene_exons.gff3.gz, http://genome.jgi.doe.gov/pages/dynamicOrganismDownload.jsf?organism=Bdistachyon) and -ud 0 command. A virtual *B. stacei* genome was constructed by replacing the nucleotides of the homoeologous SNPs in the *B. distachyon* genome with those in the *B. stacei* genome using original Perl script (Additional file 4).

Ka* and *Ks* calculation and estimation of the speciation time of *B. distachyon* and *B. stacei

The *Ka* and *Ks* values between genes of homoeolog groups were calculated with Ka_Ks Calculator (v.2.0) [62] using a modified version that implements the Yang-Nielsen algorithm (MYN) method [63]. The speciation time (T) based on the *Ks* value was estimated by using the equation $T = Ks/2\lambda$ where $\lambda = 6.1 \times 10^{-9}$ [64, 65], based on the average *Ks* value of each homoeolog group.

Read count and reads per million calculations

To obtain high-quality reads, the RNA-Seq reads were trimmed by cutting bases off from the start and end of reads if quality ≤20, and removing the reads with final read length <50 using Trimmomatic with -thread 4 LEADING: 20 TRAILING: 20 MINLEN: 50 commands. The trimmed reads were mapped to Bd21 and the virtual *B. stacei* genome using TMAP with mapall -n 4 -v -Y -u -o 2 stage1 map4 commands; *B. distachyon* reads were mapped to the Bd21 genome, *B. stacei* reads were mapped to the virtual *B. stacei* genome, and *B. hybridum* reads were mapped to both these genomes. The expression levels of the homoeologs in *B. hybridum* were quantified by classifying the RNA-Seq reads into the following three groups based on the number of mismatches between read and both genomes using original Perl script (Additional file 5): Bd-subgenome origin reads, Bs-genome origin reads, and unclassified reads (Additional file 1; Figure S5B). The mapping data from the *B. distachyon* reads, *B. stacei* reads, Bd-subgenome origin reads, and Bs-genome origin reads were subjected to read count using featureCounts (v.1.4.6) [66] with the gene structural annotation of Bd21. As the MAV, which is the average of the expression values in both ancestors, the total read count data from the *B. distachyon* and *B. stacei* reads were used, and as the entire expression value of the homoeolog group in *B. hybridum*, the total read count data for the Bd- and the Bs-subgenome origin reads were used (Additional file 1: Figure S5A). The reads per million mapped reads (RPM) values were calculated for all annotated genes based on the read count data. Genes, MAV, homoeologs, and homoeolog groups with RPM ≥ 1 in all

three biological replicates were defined as expressed.

Validation of homoeolog-specific gene expression in *B. hybridum*

The ratios of expression between homoeologs were verified using a digital PCR system in combination with the TaqMan® SNP Genotyping Assay method (Applied Biosystems, CA, USA) [67]. PCR primers were designed at conserved regions between homeologs, and targeted homoelogous SNP was included in the amplified region. Two dye-labeled (FAM and VIC) TaqMan probes were designed for quantifying homoeologous SNPs using the TaqMan MGB SNP Kit (Applied Biosystems, CA, USA). cDNA was prepared from *B. hybridum* RNA sampled after 3-day exposure to heat stress (with three biological replicates) using the SuperScript® IV Reverse Transcriptase (Invitrogen, CA, USA), and was used for quantitative PCR. The thermocycling conditions were initiated at 96°C for 10 min, followed by 39 cycles of annealing and extension at 56 °C for 2 min and denaturation at 98°C for 30 sec, followed by a final extension at 56°C for 2 min. Homoeologous SNPs in the cDNA amplicons were detected with QuantStudio™ 3D (Life Technologies Japan). The PCR primers and TaqMan probes used in this assay are listed in Additional file 2: Table S14.

Differentially expressed genes analysis

Differentially expressed genes between the three *Brachypodium* species, homoeolog groups, and growth conditions were identified using the DESeq2 package (v.1.10.1) [68] in R (v.3.2.4) with the Wald test based

on the read count data. FDR for each comparison was calculated by adjusting the *p*-value using the Benjamini-Hochberg procedure. Genes with an FDR ≤ 0.001 were defined as differentially expressed.

GO enrichment analysis

GO terms for the *B. distachyon* genes were used from the gene annotation information downloaded from Phytozome (Bdistachyon_314_v3.1.annotation_info.txt, http://genome.jgi.doe.gov/pages/dynamicOrganismDownload.jsf?organism=Bdistachyon). Additional GO terms were associated with the *B. distachyon* genes using GO terms that related to transcripts for *A. thaliana* and rice in the “Best-hit-arabi-name” and “Best-hit-rice-name” row in the annotation file. The GO terms for *A. thaliana* and rice were used from the gene annotation information downloaded from Phytozome (Athaliana_167_TAIR10.annotation_info.txt, http://genome.jgi.doe.gov/pages/dynamicOrganismDownload.jsf?organism=Athaliana, and Osativa_204_v7.0.annotation_info.txt, http://genome.jgi.doe.gov/pages/dynamicOrganismDownload.jsf?organism=Osativa). To reduce bias, GO terms that were assigned to more than 3000 *Brachypodium* genes were excluded. Enriched GO terms for selected genes were identified using BLAST2GO (v.3.3.5) [69] with the Fisher’s exact test. All of the annotated genes in the Bd21 genome were used as a reference set. The enriched GO terms were summarized using the web-based tool REVIGO (http://revigo.irb.hr/) if more than 10 enriched GO terms were found. GO

annotations of the *B. distachyon* genes are provided in Additional file 6.

Availability of supporting data

All sequencing data were archived at DDBJ under the accession numbers DRA005717 and DRA005699. The other datasets supporting the results of this article are included as additional files. Additional files 1 and 2 provide Supplementary Figures and Supplementary Tables, respectively. Additional file 3 provides the homoeologous SNP dataset used in this study. Additional files 4 and 5 provide the original Perl script codes used in this study. Additional file 6 provides the GO annotations of the *B. distachyon* genes used in this study.

Additional files

Additional file 1: Supplementary Figures

Figure S1. Phylogenetic relationships among the three *Brachypodium* species.

Figure S2. Results of homoeologous SNP identification.

Figure S3. Overview of genomic sequence data analysis.

Figure S4. Location and effect of homoeologous SNPs.

Figure S5. Overview of RNA-Seq data analysis.

Figure S6. Classification of the read origin of the *B. hybridum* RNA reads from leaf and root tissues.

Figure S7. Log_2 fold-change distribution of homoeolog expression in *B. hybridum* under heat stress condition for 3 days.

Figure S8. Gene expression profiles of the Bd- and Bs-homoeologs in homoeolog groups showing significantly higher expression in *B. hybridum* than in *B. distachyon* under heat stress condition for 15 days.

Figure S9. Gene expression profiles of the *Brachypodium HsfA2* and putative *HsfA2*-targeted genes at 3 days after heat stress exposure.

Figure S10. Quantitative detection of fluorescence of homoeologs in *B. hybridum*.

Figure S11. Expression ratios of Bd- and Bs-homoeologs based on RNA-Seq analysis and TaqMan SNP Genotyping Assay.

Additional file 2: Supplementary Tables

Table S1. Summary of the sequencing analysis and accession numbers

Table S2. Summary of the whole genome sequencing analysis and mapping results

Table S3. Results of SNP calling

Table S4. Numbers of variant effects in genes

Table S5. Enriched GO terms in the biological process ontology of homoeolog groups showing transgressive expression in *B. hybridum* leaf under normal condition

Table S6. Enriched GO terms in the biological process ontology of homoeolog groups showing transgressive expression in *B. hybridum* root under normal condition

Table S7. Enriched GO terms in the biological process ontology of genes showing significantly higher expression in *B. stacei* than in *B. distachyon* at 15 days after heat stress exposure

Table S8. Enriched GO terms in the biological process ontology of homoeolog groups showing significantly higher expression in *B. hybridum* than in *B. distachyon* at 15 days after heat stress exposure

Table S9. Enriched GO terms in the biological process ontology of homoeolog groups showing non-additive expression in *B. hybridum* leaf under normal condition

Table S10. Enriched GO terms in the biological process ontology of homoeolog groups showing non-additive expression in *B. hybridum* root under normal condition

Table S11. Enriched GO terms in the biological process ontology of homoeolog groups showing non-additive expression in *B. hybridum* leaf at 3 days after heat stress exposure

Table S12. Enriched GO terms in the biological process ontology of homoeolog groups showing non-additive expression in *B. hybridum* leaf at 15 days after heat stress exposure

Table S13. Genes showing higher expression in the *B. stacei* genome than in the *B. distachyon* genome in the three *Brachypodium* species at 3 days after heat stress exposure

Table S14. Amplicon primer and TaqMan probes used in this study

Additional file 3: Homoeologous SNP dataset used in this study

Additional file 4: Original Perl script code used to construct the virtual *B. stacei* genome by replacing the nucleotides of the homoeologous SNPs in the *B. distachyon* genome with those in the *B. stacei*

genome

Additional file 5: Original Perl script code used to classify the RNA-Seq reads of *B. hybridum* into the Bd-subgenome origin reads, Bs-subgenome origin reads, and unclassified reads

Additional file 6: GO annotations of the *B. distachyon* genes used in this study

Abbreviations

DNA, Deoxyribonucleic acid; FDR, False discovery rate; GO, Gene ontology; MAV, Mid-ancestral value; MAY, Million years ago; PCC, Pearson's correlation coefficient; RNA, Ribonucleic acid; RPM, Reads per million mapped reads; SNP, Single nucleotide polymorphism; UTR, Untranslated region

Competing interests

The authors declare that they have no competing interests.

Author contributions

KT and KM designed the work. KT and YO grew and sampled the plants. MS, YU-Y and YO generated the genome sequencing data. KT and KI generated the RNA sequencing data. KT performed in silico analyses using the genome and RNA sequencing data. KM directed this study. KT and KM wrote the manuscript with all authors.

Acknowledgments

The authors thank the National Plant Germplasm System of USDA-ARS, David F. Garvin and Pilar Catalán for providing *Brachypodium* seeds. This work was partially supported by grants-in-aid for Young Scientists (A) (grant no. 26712003 to KM) from the Japan Society for the Promotion of Science (JSPS) and by funds to KM from the Advanced Low Carbon Technology Research and Development Program (ALCA, J2013403) of the Japan Science and Technology Agency (JST). This work was also supported by RIKEN Junior Research Associate Program.

References

1. Soltis PS and Soltis DE. Polyploidy and genome evolution. Berlin ; New York: Springer Verlag; 2012.
2. Comai L. The advantages and disadvantages of being polyploid. Nat Rev Genet. 2005;6 11:836-46.
3. Leitch AR and Leitch IJ. Perspective - Genomic plasticity and the diversity of polyploid plants. Science. 2008;320 5875:481-3.
4. Bowman JL, Floyd SK and Sakakibara K. Green genes-comparative genomics of the green branch of life. Cell. 2007;129 2:229-34.
5. Adams KL and Wendel JF. Polyploidy and genome evolution in plants. Curr Opin Plant Biol. 2005;8

2:135-41.

6. Segraves KA. The effects of genome duplications in a community context. New Phytol. 2017.

7. Bowers JE, Chapman BA, Rong J and Paterson AH. Unravelling angiosperm genome evolution by phylogenetic analysis of chromosomal duplication events. Nature. 2003;422 6930:433-8.

8. Otto SP. The evolutionary consequences of polyploidy. Cell. 2007;131 3:452-62.

9. Jiao Y, Wickett NJ, Ayyampalayam S, Chanderbali AS, Landherr L, Ralph PE, et al. Ancestral polyploidy in seed plants and angiosperms. Nature. 2011;473 7345:97-100.

10. Soltis PS and Soltis DE. The role of hybridization in plant speciation. Annu Rev Plant Biol. 2009;60:561-88.

11. Fawcett JA, Maere S and Van de Peer Y. Plants with double genomes might have had a better chance to survive the Cretaceous-Tertiary extinction event. Proc Natl Acad Sci U S A. 2009;106 14:5737-42.

12. Vanneste K, Baele G, Maere S and Van de Peer Y. Analysis of 41 plant genomes supports a wave of successful genome duplications in association with the Cretaceous-Paleogene boundary. Genome Res. 2014;24 8:1334-47.

13. Chen ZJ. Molecular mechanisms of polyploidy and hybrid vigor. Trends Plant Sci. 2010;15 2:57-71.

14. Chen ZJ. Genomic and epigenetic insights into the molecular bases of heterosis. Nat Rev Genet. 2013;14 7:471-82.

15. Chen ZJ and Birchler JA. Polyploid and hybrid genomics. Ames, Iowa: Wiley-Blackwell; 2013.

16. Solhaug EM, Ihinger J, Jost M, Gamboa V, Marchant B, Bradford D, et al. Environmental Regulation of Heterosis in the Allopolyploid Arabidopsis suecica. Plant Physiol. 2016;170 4:2251-63.

17. Maricle BR, Crosier JJ, Bussiere BC and Lee RW. Respiratory enzyme activities correlate with anoxia tolerance in salt marsh grasses. J Exp Mar Bio Ecol. 2006;337 1:30-7.

18. Ainouche ML, Fortune PM, Salmon A, Parisod C, Grandbastien MA, Fukunaga K, et al. Hybridization, polyploidy and invasion: lessons from Spartina (Poaceae). Biol Invasions. 2009;11 5:1159-73.

19. Dubcovsky J and Dvorak J. Genome plasticity a key factor in the success of polyploid wheat under domestication. Science. 2007;316 5833:1862-6.

20. Yang C, Zhao L, Zhang H, Yang Z, Wang H, Wen S, et al. Evolution of physiological responses to salt stress in hexaploid wheat. Proc Natl Acad Sci U S A. 2014;111 32:11882-7.

21. Stebbins GL. Variation and evolution in plants. New York,: Columbia University Press; 1950.

22. Stebbins GL. Chromosomal evolution in higher plants. Reading, Mass.: Addison-Wesley; 1971.

23. Yoo MJ, Liu X, Pires JC, Soltis PS and Soltis DE. Nonadditive gene expression in polyploids. Annu Rev Genet. 2014;48:485-517.

24. Wang J, Tian L, Lee HS, Wei NE, Jiang H, Watson B, et al. Genomewide nonadditive gene regulation in Arabidopsis allotetraploids. Genetics. 2006;172 1:507-17.

25. Chague V, Just J, Mestiri I, Balzergue S, Tanguy AM, Huneau C, et al. Genome-wide gene expression changes in genetically stable synthetic and natural wheat allohexaploids. New Phytol. 2010;187 4:1181-94.

26. Kim ED and Chen ZJ. Unstable transcripts in Arabidopsis allotetraploids are associated with nonadditive gene expression in response to abiotic and biotic stresses. PLoS One. 2011;6 8:e24251.

27. Grover CE, Gallagher JP, Szadkowski EP, Yoo MJ, Flagel LE and Wendel JF. Homoeolog expression bias and expression level dominance in allopolyploids. New Phytol. 2012;196 4:966-71.

28. Madlung A. Polyploidy and its effect on evolutionary success: old questions revisited with new tools. Heredity (Edinb). 2013;110 2:99-104.

29. Yoo MJ, Szadkowski E and Wendel JF. Homoeolog expression bias and expression level dominance in allopolyploid cotton. Heredity (Edinb). 2013;110 2:171-80.

30. Zhang D, Pan Q, Tan C, Zhu B, Ge X, Shao Y, et al. Genome-Wide Gene Expressions Respond Differently to A-subgenome Origins in Brassica napus Synthetic Hybrids and Natural Allotetraploid. Front Plant Sci. 2016;7:1508.

31. Bardil A, de Almeida JD, Combes MC, Lashermes P and Bertrand B. Genomic expression dominance in the natural allopolyploid Coffea arabica is massively affected by growth temperature. New Phytol. 2011;192 3:760-74.

32. Li A, Liu D, Wu J, Zhao X, Hao M, Geng S, et al. mRNA and Small RNA Transcriptomes Reveal

Insights into Dynamic Homoeolog Regulation of Allopolyploid Heterosis in Nascent Hexaploid Wheat. Plant Cell. 2014;26 5:1878-900.

33. Zhang H, Gou X, Zhang A, Wang X, Zhao N, Dong Y, et al. Transcriptome shock invokes disruption of parental expression-conserved genes in tetraploid wheat. Sci Rep. 2016;6:26363.

34. Akama S, Shimizu-Inatsugi R, Shimizu KK and Sese J. Genome-wide quantification of homeolog expression ratio revealed nonstochastic gene regulation in synthetic allopolyploid Arabidopsis. Nucleic Acids Res. 2014;42 6:e46.

35. Paape T, Hatakeyama M, Shimizu-Inatsugi R, Cereghetti T, Onda Y, Kenta T, et al. Conserved but Attenuated Parental Gene Expression in Allopolyploids: Constitutive Zinc Hyperaccumulation in the Allotetraploid Arabidopsis kamchatica. Mol Biol Evol. 2016;33 11:2781-800.

36. Lopez-Alvarez D, Lopez-Herranz ML, Betekhtin A and Catalan P. A DNA barcoding method to discriminate between the model plant Brachypodium distachyon and its close relatives B. stacei and B. hybridum (Poaceae). PLoS One. 2012;7 12:e51058.

37. Lopez-Alvarez D, Manzaneda AJ, Rey PJ, Giraldo P, Benavente E, Allainguillaume J, et al. Environmental niche variation and evolutionary diversification of the Brachypodium distachyon grass complex species in their native circum-Mediterranean range. Am J Bot. 2015;102 7:1073-88.

38. Catalan P, Muller J, Hasterok R, Jenkins G, Mur LA, Langdon T, et al. Evolution and taxonomic split of the model grass Brachypodium distachyon. Ann Bot. 2012;109 2:385-405.

39. Catalan P, Chalhoub B, Chochois V, Garvin DF, Hasterok R, Manzaneda AJ, et al. Update on the genomics and basic biology of Brachypodium International Brachypodium Initiative (IBI). Trends Plant Sci. 2014;19 7:414-8.

40. Catalan P, Lopez-Alvarez D, Bellosta C and Villar L. Updated taxonomic descriptions, iconography, and habitat preferences of Brachypodium distachyon, B-stacei, and B-hybridum (Poaceae). An Jardin Bot Madrid. 2016;73 1.

41. Betekhtin A, Jenkins G and Hasterok R. Reconstructing the Evolution of Brachypodium Genomes Using Comparative Chromosome Painting. PLoS One. 2014;9 12:e115108.

42. Vogel J. Genetics and genomics of Brachypodium. New York, NY: Springer Science+Business Media; 2016.

43. Dinh Thi VH, Coriton O, Le Clainche I, Arnaud D, Gordon SP, Linc G, et al. Recreating Stable Brachypodium hybridum Allotetraploids by Uniting the Divergent Genomes of B. distachyon and B. stacei. PLoS One. 2016;11 12:e0167171.

44. Schramm F, Ganguli A, Kiehlmann E, Englich G, Walch D and von Koskull-Doring P. The heat stress transcription factor HsfA2 serves as a regulatory amplifier of a subset of genes in the heat stress response in Arabidopsis. Plant Mol Biol. 2006;60 5:759-72.

45. Charng YY, Liu HC, Liu NY, Chi WT, Wang CN, Chang SH, et al. A heat-inducible transcription factor, HsfA2, is required for extension of acquired thermotolerance in Arabidopsis. Plant Physiol.

2007;143 1:251-62.

46. Chauhan H, Khurana N, Agarwal P and Khurana P. Heat shock factors in rice (Oryza sativa L.): genome-wide expression analysis during reproductive development and abiotic stress. Mol Genet Genomics. 2011;286 2:171-87.

47. Lowry E and Lester SE. The biogeography of plant reproduction: potential determinants of species' range sizes. J Biogeogr. 2006;33 11:1975-82.

48. te Beest M, Le Roux JJ, Richardson DM, Brysting AK, Suda J, Kubesova M, et al. The more the better? The role of polyploidy in facilitating plant invasions. Ann Bot. 2012;109 1:19-45.

49. Marchant DB, Soltis DE and Soltis PS. Patterns of abiotic niche shifts in allopolyploids relative to their progenitors. New Phytol. 2016;212 3:708-18.

50. Bakker EG, Montgomery B, Nguyen T, Eide K, Chang J, Mockler TC, et al. Strong population structure characterizes weediness gene evolution in the invasive grass species Brachypodium distachyon. Mol Ecol. 2009;18 12:2588-601.

51. Meimberg H, Rice KJ, Milan NF, Njoku CC and McKay JK. Multiple origins promote the ecological amplitude of allopolyploid Aegilops (Poaceae). Am J Bot. 2009;96 7:1262-73.

52. Song LL, Jiang YL, Zhao HQ and Hou MF. Acquired thermotolerance in plants. Plant Cell Tiss Org. 2012;111 3:265-76.

53. Driedonks N, Xu J, Peters JL, Park S and Rieu I. Multi-Level Interactions Between Heat Shock

Factors, Heat Shock Proteins, and the Redox System Regulate Acclimation to Heat. Front Plant Sci. 2015;6:999.

54. Jacob P, Hirt H and Bendahmane A. The heat-shock protein/chaperone network and multiple stress resistance. Plant Biotechnol J. 2017;15 4:405-14.

55. Ohama N, Sato H, Shinozaki K and Yamaguchi-Shinozaki K. Transcriptional Regulatory Network of Plant Heat Stress Response. Trends Plant Sci. 2017;22 1:53-65.

56. Bolger AM, Lohse M and Usadel B. Trimmomatic: a flexible trimmer for Illumina sequence data. Bioinformatics. 2014;30 15:2114-20.

57. Li H, Handsaker B, Wysoker A, Fennell T, Ruan J, Homer N, et al. The Sequence Alignment/Map format and SAMtools. Bioinformatics. 2009;25 16:2078-9.

58. Quinlan AR. BEDTools: The Swiss-Army Tool for Genome Feature Analysis. Curr Protoc Bioinformatics. 2014;47:11 2 1- 2 34.

59. Koboldt DC, Chen K, Wylie T, Larson DE, McLellan MD, Mardis ER, et al. VarScan: variant detection in massively parallel sequencing of individual and pooled samples. Bioinformatics. 2009;25 17:2283-5.

60. Cingolani P, Platts A, Wang le L, Coon M, Nguyen T, Wang L, et al. A program for annotating and predicting the effects of single nucleotide polymorphisms, SnpEff: SNPs in the genome of Drosophila melanogaster strain w1118; iso-2; iso-3. Fly (Austin). 2012;6 2:80-92.

61. Trapnell C, Williams BA, Pertea G, Mortazavi A, Kwan G, van Baren MJ, et al. Transcript assembly and quantification by RNA-Seq reveals unannotated transcripts and isoform switching during cell differentiation. Nat Biotechnol. 2010;28 5:511-5.

62. Wang D, Zhang Y, Zhang Z, Zhu J and Yu J. KaKs_Calculator 2.0: a toolkit incorporating gamma-series methods and sliding window strategies. Genomics Proteomics Bioinformatics. 2010;8 1:77-80.

63. Zhang Z, Li J and Yu J. Computing Ka and Ks with a consideration of unequal transitional substitutions. BMC Evol Biol. 2006;6:44.

64. Lynch M and Conery JS. The evolutionary fate and consequences of duplicate genes. Science. 2000;290 5494:1151-5.

65. He N, Zhang C, Qi X, Zhao S, Tao Y, Yang G, et al. Draft genome sequence of the mulberry tree Morus notabilis. Nat Commun. 2013;4:2445.

66. Liao Y, Smyth GK and Shi W. featureCounts: an efficient general purpose program for assigning sequence reads to genomic features. Bioinformatics. 2014;30 7:923-30.

67. Schleinitz D, Distefano JK and Kovacs P. Targeted SNP genotyping using the TaqMan(R) assay. Methods Mol Biol. 2011;700:77-87.

68. Love MI, Huber W and Anders S. Moderated estimation of fold change and dispersion for RNA-seq data with DESeq2. Genome Biol. 2014;15 12:550.

69. Conesa A, Gotz S, Garcia-Gomez JM, Terol J, Talon M and Robles M. Blast2GO: a universal tool for annotation, visualization and analysis in functional genomics research. Bioinformatics. 2005;21 18:3674-6.

Figure legends

Figure 1. Effect of heat stress on the growth of the three *Brachypodium* species.

Fresh weight of *B. distachyon*, *B. stacei*, and *B. hybridum* grown under normal (22°C) and heat stress (32°C) conditions at 3 (**A**) and 15 (**B**) days after stress exposure. Data are the mean value ± standard deviation for twelve individuals. Statistical differences are indicated by an asterisk ($P < 0.01$, *t*-test). Bd, *B. distachyon*; Bs, *B. stacei*; Bh, *B. hybridum*.

Figure 2. Summary of genome sequence data.

Mapping results and polymorphic features of the genomic reads from *B. hybridum* and *B. stacei* superimposed to the reference genome sequence of Bd21 (100-kb sliding windows). (1) Number of homoeologous SNPs. (2) Number of heterogenic SNPs between *B. distachyon* and *B. hybridum*. (3) Average number of *B. hybridum* reads mapped to the reference genome. (4) Number of homogenic SNPs between *B. distachyon* and *B. stacei*. (5) Average number of *B. stacei* reads mapped to the reference genome. (6) Density distribution of the Bd21 annotated genes.

Figure 3. Global gene expression patterns in *B. hybridum* compared with its ancestors.

(**A** and **B**) Venn diagram of genes expressed in the MAV (left) and homoeolog groups expressed in *B. hybridum* (right) in leaf (**A**) and root (**B**) tissues. (**C** and **D**) Additive and non-additive gene expression patterns in leaf (**C**) and root (**D**) tissues of *B. hybridum*. Genes (homoeolog groups) expressed in both the MAV and *B. hybridum* (17,256 genes (homoeolog groups)) were analyzed. (**E** and **F**) Ancestral and non-ancestral gene expression patterns in leaf (**E**) and root (**F**) tissues in *B. hybridum*. Genes (homoeolog groups) expressed in both the MAV and *B. hybridum* (18,021 genes (homoeolog groups)) were analyzed. “=” indicates that the gene expression level is not significantly different between the compared groups. “>” and “<” indicate that the gene expression level is significantly different between the compared groups. The significance threshold is set at FDR ≤ 0.001. Bd, *B. distachyon*; Bs, *B. stacei*; MAV, Mid-ancestral value; BhBd, Bd-homoeologs in *B. hybridum*; BhBs, Bs-homoeologs in *B. hybridum*; Bh, *B. hybridum*.

Figure 4. Heat stress response in *B. hybridum* as analyzed using the Bs-homoeologs.

(**A**) Heatmap of Pearson’s correlation coefficients based on the gene expression profiles of each species at 3 days after heat stress exposure. Pearson correlation coefficient values between different conditions are shown in yellow squares. (**B**) Gene expression profiles of the Bd- and Bs-homoeologs in homoeolog groups showing significantly higher expression in *B. hybridum* than in *B. distachyon* under heat stress condition at 3 days

after stress exposure. Each dot represents the average expression value of three biological replicates. Blue dots represent genes showing significantly higher expression in Bd-homoeologs than in Bs-homoeologs and red dots represent genes showing significantly higher expression in Bs-homoeologs than in Bd-homoeologs (FDR ≤ 0.001). (**C**) Genes showing significantly higher expression in the *B. stacei* genome than in the *B. distachyon* genome in the three *Brachypodium* species under heat stress condition for 3 days. The left circle represents genes showing significantly higher expression in *B. stacei* than in *B. distachyon*. The right circle represents genes showing significantly higher expression in Bs-homoeologs than in Bd-homoeologs in homoeolog groups showing significantly higher expression in *B. hybridum* than in *B. distachyon*. (**D**) Enriched GO terms in the biological process ontology of 1,791 intersectional genes in the Venn diagram shown in (**C**). Bd, *B. distachyon*; Bs, *B. stacei*; BhBd, Bd-homoeologs in *B. hybridum*; BhBs, Bs-homoeologs in *B. hybridum*; Bh, *B. hybridum*.

Figure 5. Maintenance of transcriptional states in *B. hybridum* and *B. stacei* under long-term heat stress. (**A**) Heatmap of Pearson's correlation coefficients based on the gene expression profiles of each species at 15 days after heat stress exposure. Pearson correlation coefficient values between different conditions are marked by yellow squares. (**B** and **C**) Top 10 enriched GO terms in the biological process ontology of genes showing significantly higher expression in *B. stacei* than in *B. distachyon* (**B**) and homoeolog groups showing significantly higher expression in *B. hybridum* than in *B. distachyon* (**C**) at 15 days after heat stress exposure.

All enriched GO terms for these genes and homoeolog groups are shown in additional file 2: Table S7 and S8. Bd, *B. distachyon*; Bs, *B. stacei*; BhBd, Bd-homoeologs in *B. hybridum*; BhBs, Bs-homoeologs in *B. hybridum*; Bh, *B. hybridum*.

Figure 6. Model of phenotype and transcriptome of the three *Brachypodium* species under long-term heat stress.

Bd, *B. distachyon*; Bs, *B. stacei*; BhBd, Bd-homoeologs in *B. hybridum*; BhBs, Bs-homoeologs in *B. hybridum*; Bh, *B. hybridum*.

Figure 1

Click here to download Figure Fig1.pdf

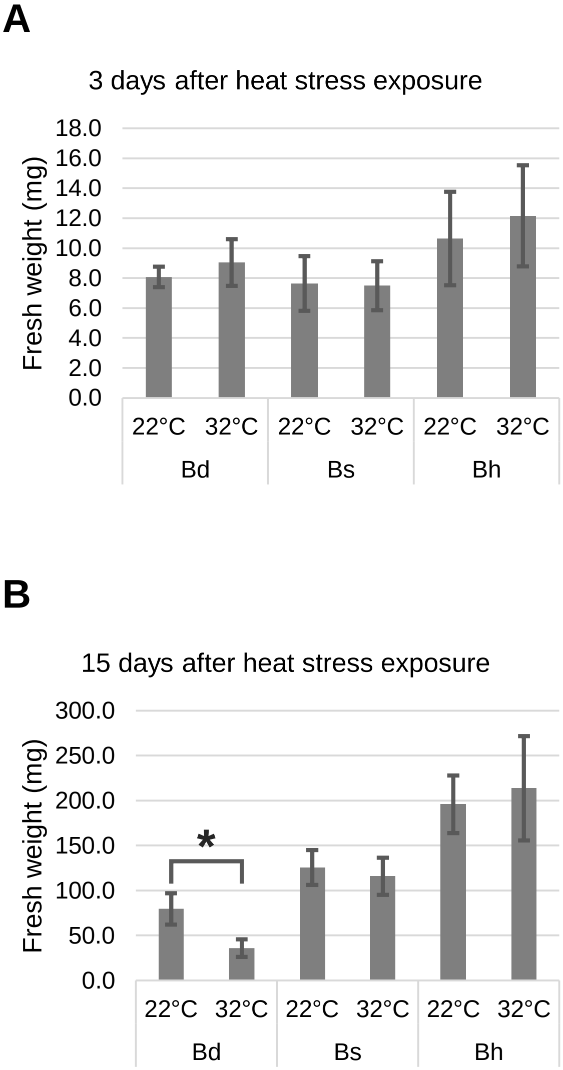

Figure 2

Click here to download Figure Fig2.pdf

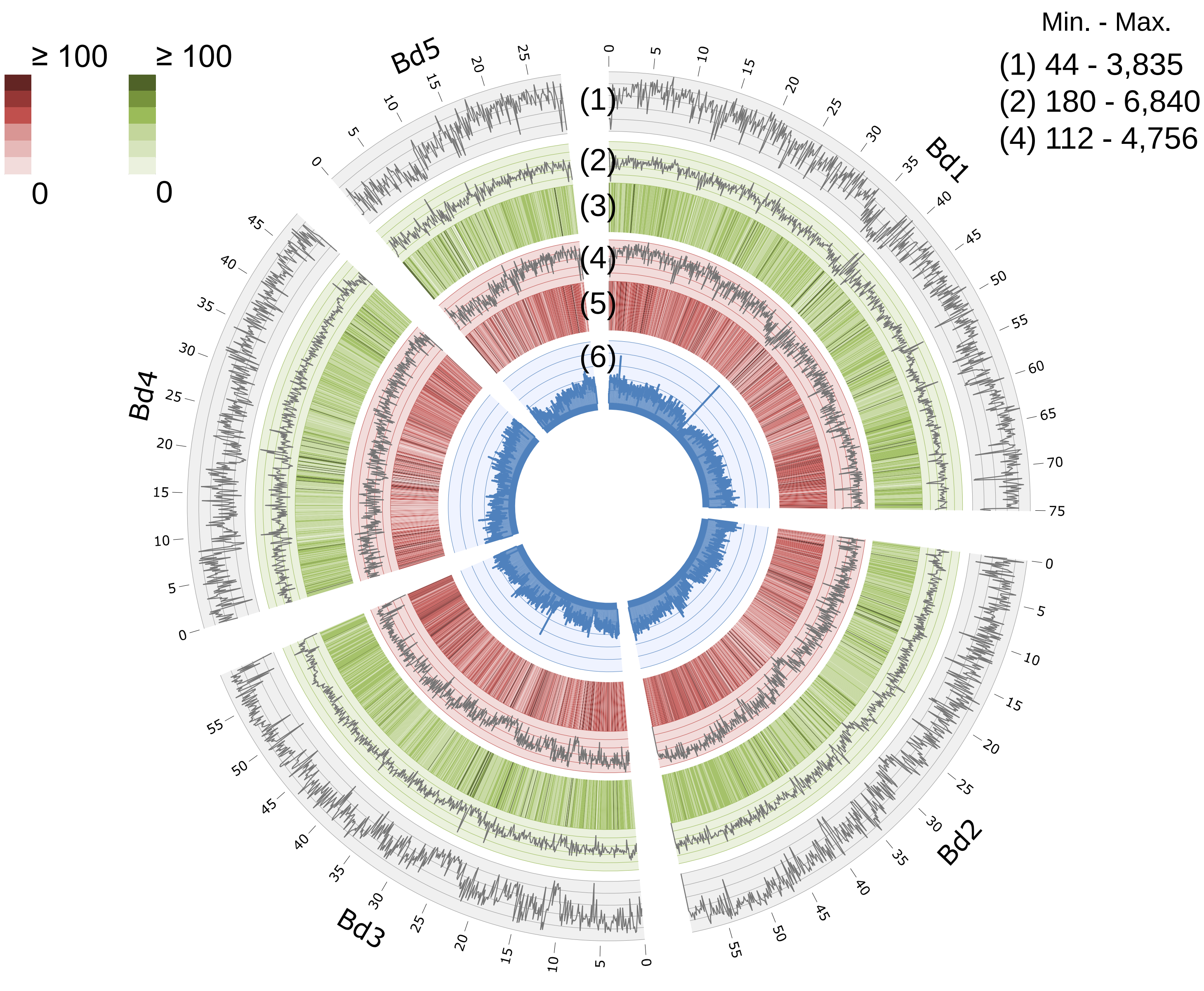

Figure 3

Click here to download Figure Fig3.pdf

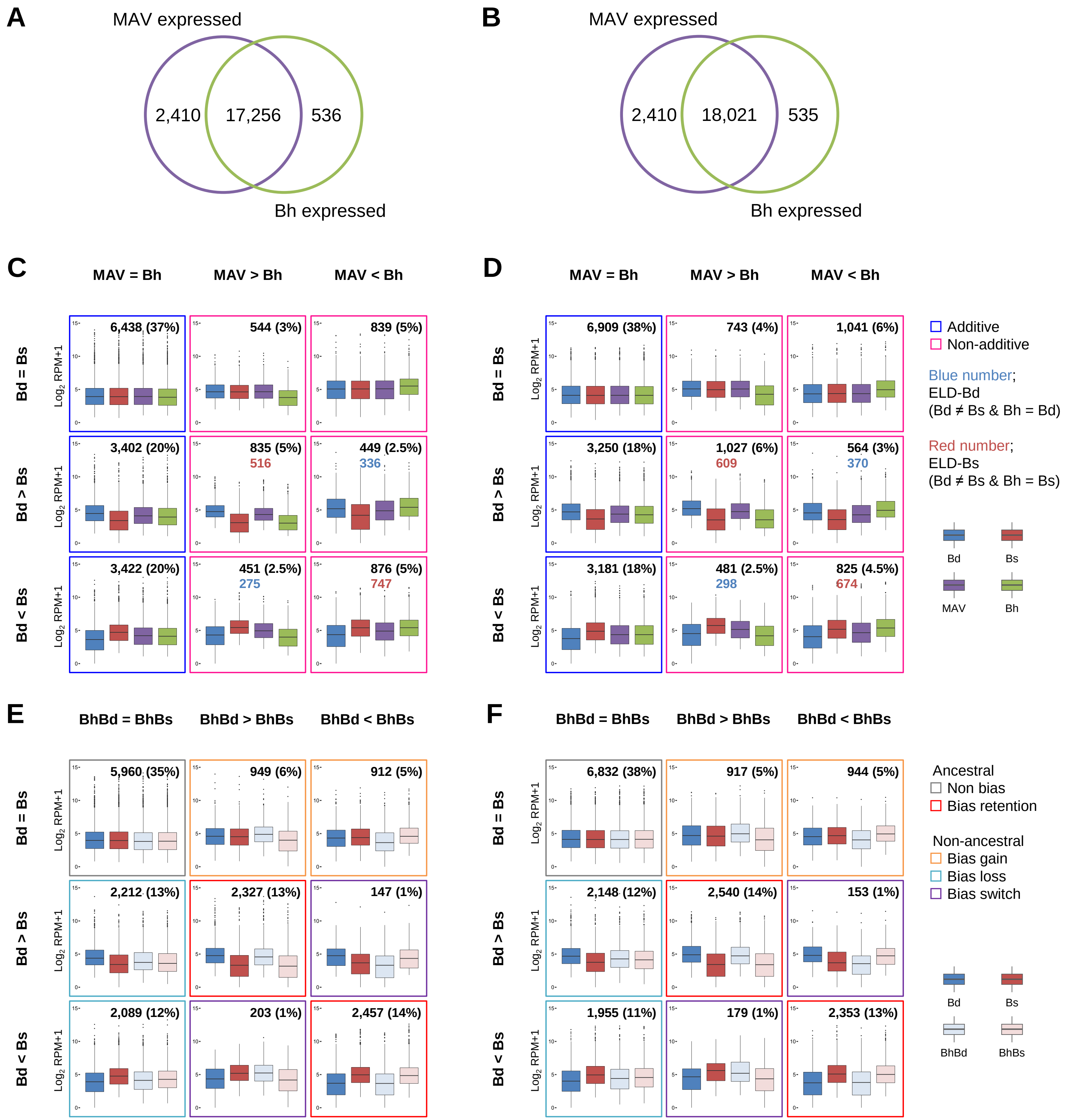

Figure 4

Click here to download Figure Fig4.pdf

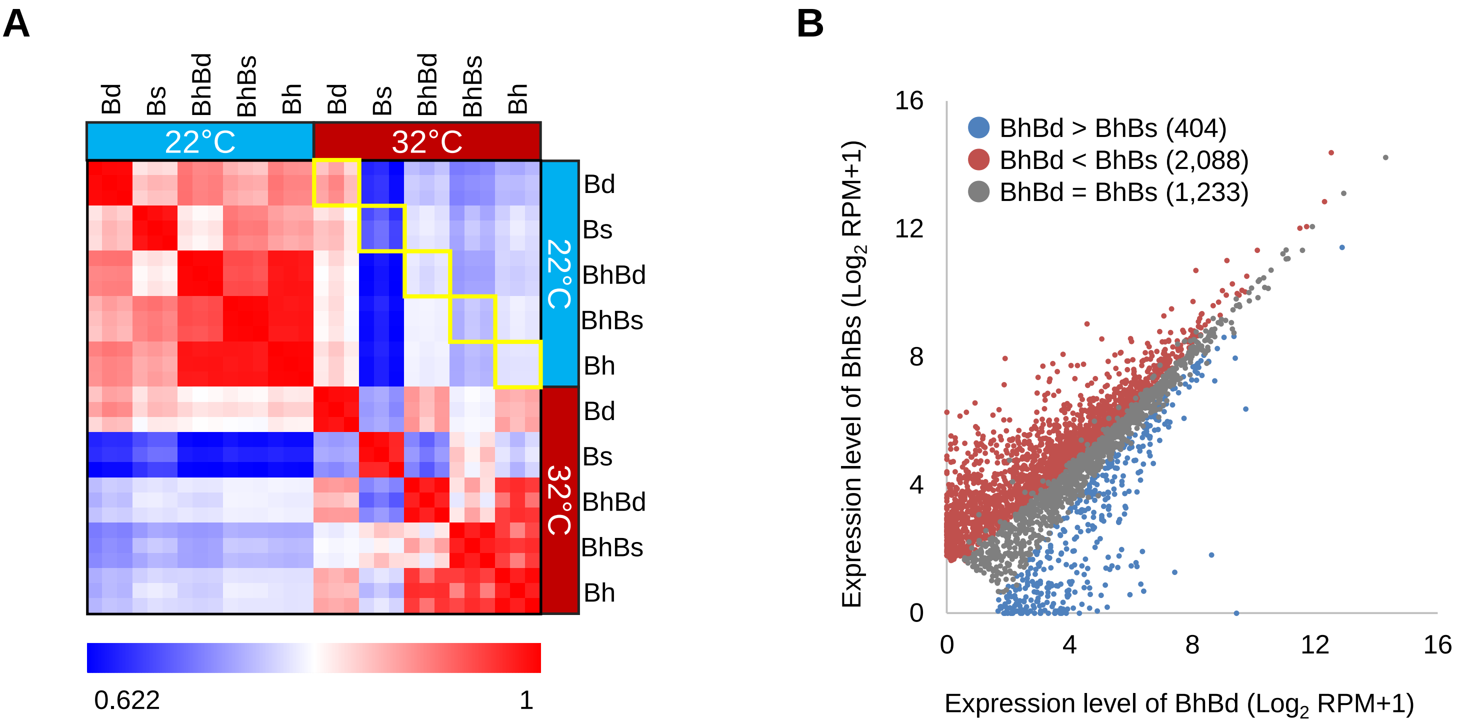

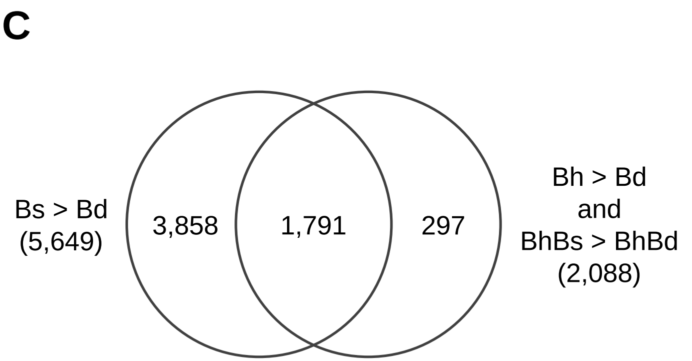

D

Genes showing higher expression in the Bs genome than in the Bd genome in the three *Brachypodium* species (1,791)

GO-ID	Term	FDR
GO:0006259	DNA metabolic process	8.26E-03
GO:0006974	cellular response to DNA damage stimulus	1.99E-02
GO:0006796	phosphate-containing compound metabolic process	2.22E-02
GO:0006793	phosphorus metabolic process	2.29E-02
GO:0033554	cellular response to stress	2.74E-02
GO:0006281	DNA repair	3.02E-02

Figure 5

Click here to download Figure Fig5.pdf

A

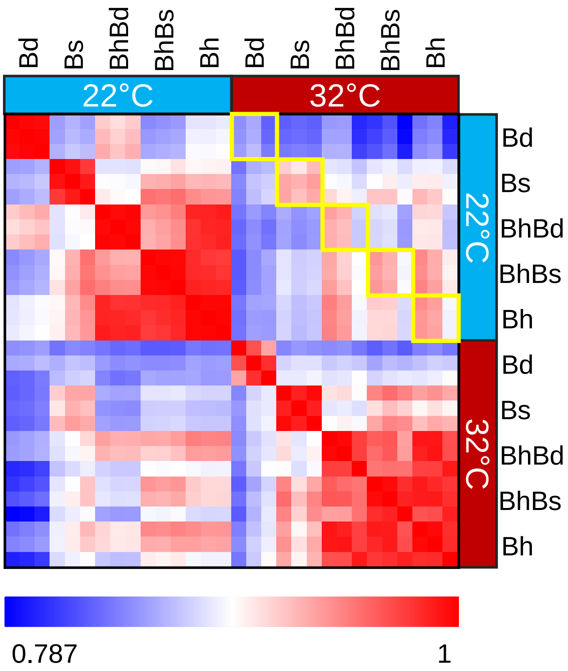

B

Genes showing higher expression in Bs than in Bd (5,517)

GO-ID	Term	FDR
GO:0015979	photosynthesis	1.03E-12
GO:0044710	single-organism metabolic process	3.46E-12
GO:0006091	generation of precursor metabolites and energy	6.73E-09
GO:0044281	small molecule metabolic process	5.69E-07
GO:0044711	single-organism biosynthetic process	6.37E-06
GO:0005975	carbohydrate metabolic process	4.21E-05
GO:0009765	photosynthesis, light harvesting	6.85E-05
GO:0044283	small molecule biosynthetic process	1.35E-04
GO:0006082	organic acid metabolic process	2.44E-04
GO:0051186	cofactor metabolic process	3.85E-04

C

Homoeolog groups showing higher expression in Bh than in Bd (4,009)

GO-ID	Term	FDR
GO:0044710	single-organism metabolic process	4.06E-15
GO:0015979	photosynthesis	2.61E-14
GO:0006091	generation of precursor metabolites and energy	2.63E-14
GO:0044281	small molecule metabolic process	2.94E-08
GO:0044711	single-organism biosynthetic process	6.21E-07
GO:0009765	photosynthesis, light harvesting	1.02E-06
GO:0006629	lipid metabolic process	2.49E-06
GO:0055114	oxidation-reduction process	2.54E-06
GO:0051186	cofactor metabolic process	4.61E-05
GO:0006732	coenzyme metabolic process	8.67E-05

Figure 6

Click here to download Figure Fig6.pdf

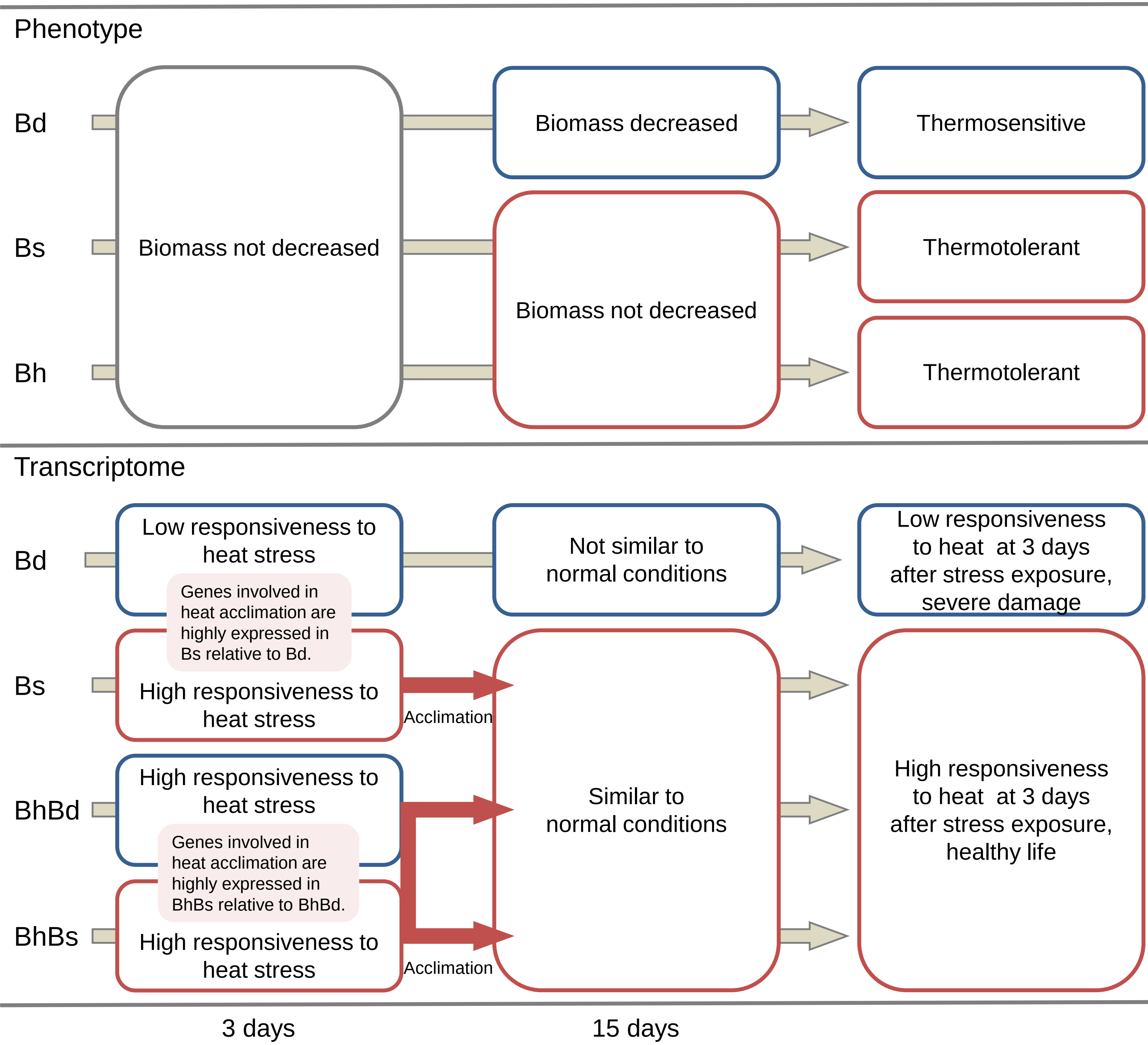

Additional file 1: Supplementary Figures

Click here to access/download
Supplementary Material
Additional file 1.pdf

Additional file 2: Supplementary Tables

Click here to access/download
Supplementary Material
Additional file 2.xlsx

Additional file 3: Homoeologous SNP dataset used in this study

Click here to access/download

Supplementary Material

Additional file 3.txt

Additional file 4: Original Perl script code used to construct the virtual <i>B. stacei</i> genome by replacing the nucleotides of the

Click here to access/download
Supplementary Material
Additional file 4.txt

Additional file 5: Original Perl script code used to classify the RNA-Seq reads of <i>B. hybridum</i> into the Bd-subgenome origin

Click here to access/download
Supplementary Material
Additional file 5.txt

Additional file 6: GO annotations of the <i>B. distachyon</i> genes used in this study

Click here to access/download

Supplementary Material

Additional file 6.txt
