# Supplementary material for: Homoeolog-specific activation of genes for heat acclimation in the allopolyploid grass Brachypodium hybridum
Source: Gigascience. 2018 Mar 8;7(4):giy020. doi: 10.1093/gigascience/giy020 (PMC5915950; doi:10.1093/gigascience/giy020)
Supplement: GIGA-D-17-00181_Revision_2.pdf [file giy020_giga-d-17-00181_revision_2.pdf]

GigaScience

Homoeolog-specific activation of genes for heat acclimation in the allopolyploid grass <i>Brachypodium hybridum</i>

--Manuscript Draft--

**Manuscript Number:**	GIGA-D-17-00181R2
**Full Title:**	Homoeolog-specific activation of genes for heat acclimation in the allopolyploid grass <i>Brachypodium hybridum</i>
**Article Type:**	Research
**Funding Information:**	Japan Society for the Promotion of Science (26712003) — Dr. Keiichi Mochida<br>Japan Science and Technology Agency (J2013403) — Dr. Keiichi Mochida
**Abstract:**	Background: Allopolyploid plants often show wider environmental tolerances than their ancestors; this is expected to be due to the merger of multiple distinct genomes with a fixed heterozygosity. The complex homoeologous gene expression could have been evolutionarily advantageous for the adaptation of allopolyploid plants. Despite multiple previous studies reporting homoeolog-specific gene expression in allopolyploid species, there are no clear examples of homoeolog-specific function in acclimation to a long-term stress condition.<br>Results: We found that the allopolyploid grass <i>Brachypodium hybridum</i> and its ancestor <i>Brachypodium stacei</i> show long-term heat stress tolerance, unlike its other ancestor, <i>Brachypodium distachyon</i>. To understand the physiological traits of <i>B. hybridum</i>, we compared the transcriptome of the three <i>Brachypodium</i> species grown under normal and heat stress conditions. We found that the expression patterns of ~26% and ~38% of the homoeolog groups in <i>B. hybridum</i> changed towards non-additive expression and non-ancestral expression, respectively, under normal condition. Moreover, we found that <i>B. distachyon</i> showed similar expression patterns between normal and heat stress conditions, whereas <i>B. hybridum</i> and <i>B. stacei</i> significantly altered their transcriptome in response to heat after 3 days of stress exposure, and homoeologs that were inherited from <i>B. stacei</i> may have contributed to the transcriptional stress response to heat in <i>B. hybridum</i>. After 15 days of heat exposure, <i>B. hybridum</i> and <i>B. stacei</i> maintained transcriptional states similar to those under normal conditions. These results suggested that an earlier response to heat that was specific to homoeologs originating from <i>B. stacei</i> contributed to cellular homeostasis under long-term heat stress in <i>B. hybridum</i>.<br>Conclusions: Our results provide insights into different regulatory events of the homoeo-transcriptome that are associated with stress acclimation in allopolyploid plants.
**Corresponding Author:**	Keiichi Mochida<br><br>JAPAN
**Corresponding Author Secondary Information:**	
**Corresponding Author's Institution:**	
**Corresponding Author's Secondary Institution:**	
**First Author:**	Kotaro Takahagi
**First Author Secondary Information:**	
**Order of Authors:**	Kotaro Takahagi<br>Komaki Inoue<br>Minami Shimizu

Powered by Editorial Manager® and ProduXion Manager® from Aries Systems Corporation

	Yukiko Uehara-Yamaguchi
	Yoshihiko Onda
	Keiichi Mochida
**Order of Authors Secondary Information:**	
**Response to Reviewers:**	February 9, 2018<br><br>Dear Dr. Hans Zauner<br><br>We thank you so much for handling and careful reading our manuscript as well as for your valuable comments and suggestions.<br><br>We addressed the points that you suggested.<br><br>1) GigaDB citation:<br>We included a citation to our upcoming GigaDB dataset (including the dummy DOI link) to the reference list, and cited this in the data availability section (Lines 464 and 707-709).<br><br>2) Please read over your manuscript carefully again to correct typos and minor grammatical issues (a few here and there).<br>We read our manuscript again and made a few corrections.<br><br>3) I wonder whether the title needs a minor clarification?<br>According to your suggestion, we changed our title to "Homoeolog-specific activation of heat acclimation mechanisms in the allopolyploid grass <i>Brachypodium hybridum</i>" to clarify the meaning of "Activation".<br><br>4) Protocols.io:<br>We are able to provide our methods used in this study. I would appreciate it if you could send us the spreadsheet. We'll fill out and send the spreadsheet.<br><br>5) URLs:<br>We moved all URLs within the text of the manuscript to the reference list and provided their access dates.<br><br>We hope that the revised manuscript meets the quality of <i>GigaSciense</i>.<br><br>Sincerely yours,<br><br>Keiichi Mochida
**Additional Information:**	
**Question**	**Response**
Are you submitting this manuscript to a special series or article collection?	No
**Experimental design and statistics**<br><br>Full details of the experimental design and statistical methods used should be given in the Methods section, as detailed in our Minimum Standards Reporting Checklist. Information essential to interpreting the data presented should be made available in the figure legends.<br><br>Have you included all the information requested in your manuscript?	Yes

Powered by Editorial Manager® and ProduXion Manager® from Aries Systems Corporation

**Resources**<br><br>A description of all resources used, including antibodies, cell lines, animals and software tools, with enough information to allow them to be uniquely identified, should be included in the Methods section. Authors are strongly encouraged to cite Research Resource Identifiers (RRIDs) for antibodies, model organisms and tools, where possible.<br><br>Have you included the information requested as detailed in our Minimum Standards Reporting Checklist?	Yes
**Availability of data and materials**<br><br>All datasets and code on which the conclusions of the paper rely must be either included in your submission or deposited in publicly available repositories (where available and ethically appropriate), referencing such data using a unique identifier in the references and in the “Availability of Data and Materials” section of your manuscript.<br><br>Have you have met the above requirement as detailed in our Minimum Standards Reporting Checklist?	Yes

Powered by Editorial Manager® and ProduXion Manager® from Aries Systems Corporation

Manuscript Click here to download Manuscript Brachypodium_28-2.docx

Homoeolog-specific activation of genes for heat acclimation in the allopolyploid grass *Brachypodium hybridum*

Kotaro Takahagi[1,2,3], Komaki Inoue[3], Minami Shimizu[2,3], Yukiko Uehara-Yamaguchi[3], Yoshihiko Onda[2,3] and Keiichi Mochida[1,2,3,4]

[1]Graduate School of Nanobioscience, Yokohama City University, 22-2 Seto, Kanazawa-ku, Yokohama, Kanagawa 236-0027, Japan.

[2]Kihara Institute for Biological Research, Yokohama City University, 641-12 Maioka-cho, Totsuka-ku, Yokohama, Kanagawa 244-0813, Japan.

[3]Cellulose Production Research Team, Biomass Engineering Research Division, RIKEN Center for Sustainable Resource Science, 1-7-22 Suehiro-cho, Tsurumi-ku, Yokohama, Kanagawa 230-0045, Japan.

[4]Institute of Plant Science and Resources, Okayama University, 2-20-1 Chuo, Kurashiki, Okayama 710-0046, Japan.

E-mail addresses

Kotaro Takahagi n155267a@yokohama-cu.ac.jp

Komaki Inoue komaki.inoue@riken.jp

Minami Shimizu minami.shimizu@riken.jp

Yukiko Uehara-Yamaguchi yukiko.uehara@riken.jp

Yoshihiko Onda yoshihiko.onda@riken.jp

Keiichi Mochida keiichi.mochida@riken.jp

Corresponding author

Keiichi Mochida, Cellulose Production Research Team, Biomass Engineering Research Division, RIKEN Center for Sustainable Resource Science, 1-7-22 Suehiro-cho, Tsurumi-ku, Yokohama, Kanagawa 230-0045, Japan. Tel: +81-45-503-9111, E-mail: keiichi.mochida@riken.jp

Abstract

Background: Allopolyploid plants often show wider environmental tolerances than their ancestors; this is expected to be due to the merger of multiple distinct genomes with a fixed heterozygosity. The complex homoeologous gene expression could have been evolutionarily advantageous for the adaptation of allopolyploid plants. Despite multiple previous studies reporting homoeolog-specific gene expression in allopolyploid species, there are no clear examples of homoeolog-specific function in acclimation to a long-term stress condition.

Results: We found that the allopolyploid grass *Brachypodium hybridum* and its ancestor *Brachypodium stacei* show long-term heat stress tolerance, unlike its other ancestor, *Brachypodium distachyon*. To understand the physiological traits of *B. hybridum*, we compared the transcriptome of the three *Brachypodium* species grown under normal and heat stress conditions. We found that the expression patterns of ~26% and ~38% of the homoeolog groups in *B. hybridum* changed towards non-additive expression and non-ancestral expression, respectively, under normal condition. Moreover, we found that *B. distachyon* showed similar expression patterns between normal and heat stress conditions, whereas *B. hybridum* and *B. stacei* significantly altered their transcriptome in response to heat after 3 days of stress exposure, and homoeologs that were inherited from *B. stacei* may have contributed to the transcriptional stress response to heat in *B. hybridum*. After 15 days of heat exposure, *B. hybridum* and *B. stacei* maintained transcriptional states similar to those under normal conditions. These results suggested that an earlier response to heat that was specific to

homoeologs originating from *B. stacei* contributed to cellular homeostasis under long-term heat stress in *B. hybridum*.

Conclusions: Our results provide insights into different regulatory events of the homoeo-transcriptome that are associated with stress acclimation in allopolyploid plants.

Keywords

Abiotic stress response; Allopolyploidy; *Brachypodium hybridum*; Heat acclimation; Homoeolog; Hybrid species; Transcriptome

Background

Polyploidy is a common phenomenon in eukaryotes, especially in plants [1-4], and is recognized as a fundamental mechanism in plant evolution and diversification [5, 6]. It has been suggested that all angiosperms have experienced one or more polyploidization events during their evolutionary history [7-10]. Recent evolutionary genomic studies have suggested that genome duplication events occurred widely in plants at the Cretaceous–Paleogene boundary, which is a major extinction event in the earth's history. Evolutionary views represent a hypothesis that plants with duplicated genomes might have a better chance for survival under global adverse conditions [11, 12].

Interspecific hybridization and subsequent genome duplication led to evolutional changes in hybrid

species that represented fixed heterozygosity. Allopolyploid plants generally show better growth vigor and stress tolerance than their ancestors [13-15]. For example, relative to their ancestors, allopolyploid *Arabidopsis* (*Arabidopsis suecica*) shows more vigorous growth, and allopolyploid *Spartina* (*Spartina anglica*) shows better tolerance to reducing conditions and sulfite-rich sediments [16-18]. Both natural and synthetic wheat (*Triticum aestivum*) have higher fitness under salt stress than their diploid and tetraploid ancestors [19, 20]. It has been suggested that allopolyploidization may have contributed to the adaptation to a wide range of environmental conditions [21, 22].

The expression levels of homoeologs in allopolyploid species often show non-additive expression that are deviated from parental additivity comparing to its ancestors [23]. The non-additive expression has been widely observed in various allopolyploid species [24-26] , and can be explained by at least three possible scenarios; total gene expression of a homoeolog group in an allopolyploid species is similar to that of one of its parental species (expression level dominance (ELD)), total gene expression is lower or higher than in both parents (transgressive expression), and uneven contribution of homoeologs to gene expression (homoeolog expression bias) [23, 27]. Through comparative gene expression analysis of total expression levels of homoeolog groups in an allopolyploid species and “mid-parental values (MPVs)” as the average expression levels of genes in its parents, parental additivity and non-additivity of homoeologs can be examined. By discrimination of homoeologous gene expression, homoeolog expression bias can be identified across tissues, developmental stages, and environmental conditions [28-30].

The contribution of expression plasticity of duplicated genes observed in diverse allopolyploid species to adaptive and ecological fitness has been debated. Comparative transcriptome analysis of the natural allopolyploid *Coffea arabica* and its ancestral species (*Coffea canephora* and *Coffea eugenioides*) revealed evidence of a genomic ELD that depends on growth temperature [31] . Comparative transcriptome analysis of hexaploid *T. aestivum* (AABBDD) and its ancestors (*Triticum turgidum* (AABB) and *Aegilops tauschii* (DD)) demonstrated differential enrichment of over-represented gene functions between the ELD genes of both parents, which suggests they may have differentially contributed to particular biological functions in hexaploid wheat [32]. Comparative gene expression analysis of a synthetic tetraploid wheat and its parental species showed transgressive expression of genes that are involved in particular biological functions such as transport, modification, and uroporphyrinogen decarboxylase activity, suggesting that transgressive gene expression may have rapidly occurred following allopolyploidization [33] . Studies on the allotetraploid *Arabidopsis kamchatica* and its ancestral species demonstrated homoeolog expression bias in genes related to adaptation to cold and heavy metal environments, which were inherited from the respective ancestors that are tolerant against cold and heavy metal stresses, respectively [34, 35].

Brachypodium hybridum (2n=30) is a natural allopolyploid that is derived from a cross between *Brachypodium distachyon* (2n=10) and *Brachypodium stacei* (2n=20) that occurred approximately 1 million years ago (MYA; Additional file 1: Figure S1) [36-43]. Although these species inhabit a circum-Mediterranean region, their environmental niches are clearly different. *B. distachyon* grows in higher-altitude,

cooler, and wetter areas, whereas *B. stacei* is found in lower-altitude, warmer, and drier areas. The hybrid species *B. hybridum* grows in areas that are overlapping as well as specific to its ancestors; this growth pattern suggests that its speciation is associated with particular environmental conditions and adaptations to diverse environmental conditions [37]. The broader growth area of *B. hybridum* compared to its ancestors led us to the hypothesis that its duplicated genes may have contributed to its adaptive and ecological fitness through gene expression changes following allotetraploidization. Specifically, in the *B. hybridum* transcriptome, each of the homoeologs may show particular expression patterns in response to abiotic stimuli, which are associated with the ancestral growth habitats and gene expression. Therefore, this trio of species has been recently proposed as a model for grass speciation via adaptation and polyploidization [39, 40, 43].

In the current study, we conducted a comparative global transcriptome analysis of *B. hybridum* and its ancestors. The analysis revealed non-additive transcriptome changes in the leaf and root tissues of *B. hybridum*. Additionally, we performed a homoeolog-specific transcriptome analysis by discriminating RNA-sequencing (RNA-Seq) reads of each homoeolog of *B. hybridum*, and determined the non-ancestral gene expression patterns. Finally, we assessed homoeolog-specific transcriptome changes in response to heat stress in *B. hybridum* and we discuss the differential regulation of the homoeo-transcriptome that is associated with heat stress tolerance in *B. hybridum* inherited from *B. stacei*.

Data Description

Plant materials

Three *Brachypodium* species were used in this study: the allotetraploid *B. hybridum* Bd14-1 and the diploid ancestors *B. distachyon* Bd21 and *B. stacei* ABR114. The accessions of these species were provided by the National Plant Germplasm System of USDA-ARS, David F. Garvin (USDA-ARS Plant Science Research Unit, University of Minnesota, USA), and Pilar Catalán (Department of Agriculture and Environment Science, High Polytechnic School of Huesca, University of Zaragoza, Spain). Dry seeds were incubated on wet filter paper in a Petri dish at 4°C in the dark for 6–7 days to synchronize germination. The germinated seeds were grown in a growth chamber at 25°C under a 16-h day photoperiod (60 $\mu mol \cdot m^{-2} \cdot s^{-1}$) for 4 days. The plants were transplanted to pots filled with autoclaved PRO-MIX BX MYCORRHIZAE (Premier Tech, Quebec, Canada). The potted plants were grown in a growth chamber at 22°C (normal condition) or 32°C (heat stress condition) under a 20-h day photoperiod (100 $\mu mol \cdot m^{-2} \cdot s^{-1}$), and watered with 5,000-fold diluted Professional Hyponex 10-30-20 (Hyponex Japan, Osaka, Japan) every three or four days.

Whole genome sequence data

Genomic DNA from the leaf tissues of *B. hybridum* and *B. stacei* were extracted using the DNeasy Plant Mini Kit (QIAGEN, Tokyo, JAPAN). Libraries for single-end DNA sequencing were obtained using the Ion Xpress™ Plus Fragment Library Kits (Life Technologies Japan, Tokyo, Japan), and semiconductor chips that

were used for sequencing were prepared using Ion OneTouch 2 System (Life Technologies Japan) and Ion P1™ Chip v2 (Life Technologies Japan). The sequencing analyses were performed using an Ion Proton sequencer (Life Technologies Japan). The genome sequence data of *B. hybridum* and *B. stacei* were archived at DDBJ under the accession number DRA005717 (Additional file 2: Table S1).

RNA-Seq data

Shoots and roots from each species grown in a growth chamber for 4 days after synchronized germination were sampled to elucidate the global homoeolog expression patterns in *B. hybridum*. Shoots and most young leaf blades from each species grown in a growth chamber under different temperature conditions (22°, normal condition, and 32°C, heat stress condition) for 3 and 15 days, respectively, after being transplanted were sampled to elucidate the homoeolog-specific transcriptional response to heat stress in *B. hybridum*. Total RNA was extracted from each sample using the RNeasy plant mini kit (QIAGEN). Poly(A) RNAs were purified using the NEBNext® Poly(A) mRNA Magnetic Isolation Module (New England Biolabs, MA, USA). The libraries for single-end strand-specific RNA-Seq were obtained using the Ion Total RNA-Seq Kit v.2 (Life Technologies Japan). Size-selected libraries were purified using the Agencourt AMPure XP (Beckman Coulter, CA, USA). Semiconductor chips that were used for sequencing were prepared using the Ion PI™ Hi-Q™ OT2 200 Kit (Life Technologies Japan), Ion PI™ Hi-Q™ Sequencing 200 Kit (Life Technologies Japan), and Ion P1™ Chip v3 (Life Technologies Japan). The sequencing analyses were performed using an

Ion Proton sequencer (Life Technologies Japan) with three biological replicates. The RNA-Seq data were archived at DDBJ under the accession number DRA005699 (Additional file 2: Table S1).

Results

B. hybridum and *B. stacei* show significant tolerance to long-term heat stress

Unlike *B. distachyon*, *B. hybridum* and *B. stacei* showed significant tolerance to long-term heat stress. The allopolyploid *B. hybridum* and its ancestor *B. stacei* grow in warmer regions, in contrast to the other ancestor *B. distachyon*, which suggests that *B. hybridum* and *B. stacei*, but not *B. distachyon*, might have adapted to high-temperature conditions. To test this hypothesis, we compared the plant biomasses of *B. hybridum*, *B. stacei*, and *B. distachyon* grown under normal (22°C) and heat stress (32°C) conditions at two time points (after 3 and 15 days of heat stress exposure). After 3 days of exposure to heat stress, the three species showed no differences in growth (Figure 1A). Conversely, after 15 days of heat exposure, *B. distachyon* showed a significant decrease in fresh weight ($P < 0.01$, *t*-test), whereas *B. hybridum* and *B. stacei* maintained their growth (Figure 1B). This result indicated that *B. hybridum* and *B. stacei* are thermotolerant species and that *B. hybridum* might have inherited this trait from *B. stacei* via allopolyploidization.

A virtual *B. stacei* genome generated from the comparative analysis of homoeologous genomes

By comparing the genomes of *B. hybridum*, *B. stacei*, and *B. distachyon*, we obtained a comprehensive map

of the homoeologous single nucleotide polymorphisms (SNPs) in these *Brachypodium* species. This map enabled the distinction of transcripts expressed from each of the homoeologs in *B. hybridum*. To determine polymorphisms between the homoeologous genomes in *B. hybridum*, we sequenced the genomes of *B. stacei* ABR114 and *B. hybridum* Bd14-1 and mapped the reads to the reference genome sequence of *B. distachyon* Bd21. We found that 85% and 91% of the genomic reads of *B. stacei* and *B. hybridum* mapped to the reference genome and covered 89% and 98% of the genic region of the reference genome, respectively, which suggests high similarity among the homoeologous genomes (Figure 2 and Additional file 2: Table S2). As for genomic polymorphisms, 11,948,285 SNPs were identified between *B. distachyon* and *B. stacei* and 10,216,010 SNPs between *B. distachyon* and *B. hybridum* (Additional file 2: Table S3). We selected 5,720,539 SNPs to discriminate the homoeologs that were homogenic to the *B. stacei* reads from those that were heterogenic to the *B. hybridum* reads (Figure 2, Additional file 1: Figure S2 and Additional file 3). By replacing the nucleotides of the homoeologous SNPs in the *B. distachyon* genome with those in *B. stacei*, we generated virtual homoeolog sequences of *B. stacei* that corresponded to *B. distachyon* counterparts (Additional file 1: Figure S3).

Genome-wide polymorphism between *B. distachyon* and *B. stacei* genomes

Through a comprehensive identification of homoeologous SNPs between *B. distachyon* and *B. stacei* genomes, we assessed the distribution of the SNPs within various genomic regions to gain insights into the

genetic diversity of the two genomes. We found that 22.8% of homoeologous SNPs are distributed within the intergenic regions, and the remaining 77.2% of the SNPs were identified in the genic regions (Additional file 1: Figure S4A). Specifically, 45.4% and 17.6% of SNPs are distributed in intronic and exonic regions, respectively, and 12.5% and 1.6% of SNPs are distributed in UTRs and splice sites, respectively. We assessed the types of polymorphism found in genic regions, and found that 26,986 genes (79% of the annotated genes in the Bd21 genome) contain nonsynonymous variations. Of these, 4,558 genes (13% of the annotated genes in the Bd21 genome) contain several variations in the *B. stacei* genome; 683, 2,980, and 895 genes show start codon loss, stop codon gain, and stop codon loss, respectively (Additional File 1: Figure S4B and Additional File 2: Table S4). Based on the synonymous substitution rate (*Ks*) between the homoeologs, we estimated a divergence time of approximately 6.4 MYA between *B. distachyon* and *B. stacei*, which is accordance with the divergence time of *B. stacei* of 5.8–16.4 MYA previously estimated by Catalan et al. 2012 [38]. Moreover, we computed the non-synonymous substitution rate (*Ka*) and *Ka*/*Ks* ratio, and identified 1,714 homoeologs whose *Ka*/*Ks* values were greater than 1, supporting their accelerated evolution. Interestingly, we found that genes involved in DNA mismatch repair with Gene Ontology (GO) terms such as proliferating cell nuclear antigen complex and DNA polymerase processivity factor activity (GO:0043626 and GO:0030337, respectively, *P*-value 4,8e-04) are significantly enriched in those genes, which suggested that DNA damage tolerance may reflect selection and adaptive divergence between *B. distachyon* and *B. stacei*.

Non-additive gene expression in *B. hybridum*

To identify gene expression changes in *B. hybridum* as a result of the tetraploidization, we examined expression levels of its homoeolog groups in comparison to mid-ancestral values (MAVs) of those estimated from each of the counterpart homoeologs in its ancestors, *B. distachyon* and *B. stacei*, and identified the homoeolog groups that showed non-additive expression in the leaf and root transcriptomes of *B. hybridum*. To this end, we performed RNA-Seq-based transcriptome analysis of leaf and root mRNA samples of *B. distachyon*, *B. stacei*, and *B. hybridum*, and quantified gene expression of the homoeologs and their counterparts in the ancestors by mapping the RNA-Seq reads to the Bd21 genome (*B. distachyon* reads), the virtual *B. stacei* genome (*B. stacei* reads), or these both genomes (*B. hybridum* reads) (Additional file 1: Figure S5A). Through comparisons of the MAV estimated from the ancestors and expression level of the homoeolog groups in *B. hybridum*, we identified 17,256 and 18,021 (50% and 53% of the annotated genes in the Bd21 genome) of genes and homoeolog groups are expressed (supported by MAV ≥ 1 and reads per million mapped reads (RPM) ≥ 1 in *B. hybridum*) in the root and leaf transcriptomes, respectively (Figures 3A and B). Then, we classified these expressed homoeolog groups according to additive or non-additive expression, and found that 3,994 and 4,681 homoeolog groups (23% and 26% of the analyzed homoeolog groups in leaf and root tissues, respectively) were non-additively expressed in the *B. hybridum* leaf and root transcriptomes, respectively (the remaining 13,262 and 13,340 homoeolog groups in leaf and root tissues, respectively, were additively expressed; Figures 3C and D). Moreover, we assessed ELD in the non-

additively expressed homoeolog groups. We found that 611 and 668 homoeolog groups in leaf and root tissues, respectively (3.5% and 3.7% of the analyzed homoeolog groups) in *B. hybridum* showed ELD, with expression levels similar to those in *B. distachyon* (ELD-Bd) (Figures 3C and D). On the other hand, 1,263 and 1,283 homoeolog groups in leaf and root tissues, respectively (7.3% and 7.1% of analyzed homoeolog groups) in *B. hybridum* showed ELD of *B. stacei* (ELD-Bs) (Figures 3C and D). These findings indicated that the number of homoeolog groups of ELD-Bs is nearly twice that of ELD-Bd, suggesting that gene expression patterns of *B. stacei* are preferentially inherited to the *B. hybridum* transcriptome in leaf and root tissues. We also identified 627 and 850 homoeolog groups in *B. hybridum* leaf and root tissues, respectively (3.6% and 4.7% of the analyzed homoeolog groups), that showed transgressive expression, i.e., higher expression than in both ancestors. Various GO terms, such as primary metabolic processes, primary cellular processes, stress response, and protein transport, localization, and translation were enriched in the set of homoeolog groups that showed transgressive expression (Additional file 2: Tables S5 and S6), suggesting that the overproduction of transcripts may affect various biological functions through the tetraploidization.

Homoeolog expression bias in *B. hybridum*

To identify homoeolog expression bias in *B. hybridum*, we sorted the *B. hybridum* RNA-Seq reads to the two homoeologous genomes of *B. distachyon* (Bd-subgenome) and *B. stacei* (Bs-subgenome) based on their sequence identities after alignments with both genomes, and quantified the expression levels of each of

homoeologs; Bd- and Bs-homoeologs in the *B. hybridum* transcriptome. Through the homoeolog-specific RNA-Seq read sorting, we discriminated 75–79% of the RNA-seq reads between the Bd-subgenome origin reads and Bs-subgenome origin reads (Additional file 1: Figures S5 and S6). We found that 6,512 and 6,296 homoeolog groups show non-ancestral expression patterns in the *B. hybridum* leaf and root transcriptomes, respectively (38% and 35% of the analyzed homoeolog groups; orange, light blue, and purple boxes in Figures 3E and F), and the remaining 10,744 and 11,725 retained the ancestral expression patterns (grey and red boxes in Figures 3E and F). In the homoeolog groups showing ancestral expression patterns, we found that similar proportions of the homoeolog groups retained the expression bias of each of the ancestral transcriptome (27% of the analyzed homoeolog groups in leaf and root tissues; red boxes in Figures 3E and F). In the homoeolog groups showing non-ancestral expression patterns, we observed bias loss in most cases in the *B. hybridum* transcriptomes (25% and 23% of the analyzed homoeolog groups in leaf and root tissues, respectively; light blue boxes in Figures 3E and F). These findings suggested that more than 30% of the homoeologous gene pairs in *B. hybridum* had changed their expression pattern towards non-ancestral expression during speciation over more than 1 MYA after the genome-scale gene duplication via allopolyploidization.

Homoeolog-specific transcriptional response to heat stress in *B. hybridum*

B. distachyon transcriptomes were not significantly different between normal (22°C) and heat stress (32°C)

conditions after 3 days of exposure to heat stress, whereas the *B. hybridum* transcriptome was noticeably altered under the heat stress as compared to the normal condition; this might reflect physiological traits inherited from *B. stacei*. When gene expression patterns of *B. distachyon*, *B. stacei*, and *B. hybridum*, as well as the Bd- and Bs-homoeologs of *B. hybridum* after 3 days of exposure to heat stress were compared, we found that the transcriptomes of *B. stacei* and *B. hybridum* showed lower correlations between normal and heat stress conditions than those of *B. distachyon* (Pearson's correlation coefficient (PCC) 0.68–0.75, 0.87, and 0.92–0.95 in *B. stacei*, *B. hybridum*, and *B. distachyon*, respectively; Figure 4A), suggesting that *B. stacei* and *B. hybridum* respond more strongly to the heat stress than *B. distachyon*. We also found that, under after 3 days of stress exposure, 5,649 genes and 3,725 homoeolog groups showed significantly higher expression in *B. stacei* and *B. hybridum,* respectively, than in *B. distachyon* (false discovery rate (FDR) ≤ 0.001). We then dissected the expression patterns of the 3,725 homoeolog groups from *B. hybridum* into Bd- and Bs-homoeologs, and found that 2,088 homoeolog groups (56% of the 3,725 homoeolog groups) were preferentially expressed by the Bs-homoeologs (Figure 4B). The distribution of fold-change expression values between Bd- and Bs-homoeologs in all expressed homoeolog groups in *B. hybridum* under heat stress for 3 days indicated that most of the homoeologs were evenly expressed by both subgenomes (Additional file 1: Figure S7A), whereas the 3,725 homoeolog groups showed abundant expression in the Bs-homoeologs (Additional file 1: Figure S7B). Additionally, of the 2,088 Bs-homoeologs, 1,791 genes were shared with the 5,649 genes that showed higher expression in *B. stacei* than in *B. distachyon*; these genes included those

specifically involved in metabolic processes as well as cellular response to stress and damage stimulus (Figures 4C and D). These results suggested that the functions of *B. hybridum* genes that were inherited from *B. stacei* may have contributed to the transcriptional stress response and associated metabolic changes in the *B. hybridum* transcriptome during the earlier response to heat stress.

Early transcriptional responses of Bs-homoeologs contribute to the maintenance of cellular functions in *B. hybridum* under long-term heat stress

After 15 days of exposure to heat stress, *B. hybridum* and *B. stacei* maintained their transcriptional states similar to those under normal condition, in contrast to the severely damaged cellular system of *B. distachyon*. When we compared the expression patterns between *B. distachyon*, *B. stacei*, and *B. hybridum* after 15 days of exposure to heat stress, we found that *B. stacei* and *B. hybridum* showed similar expression patterns between stress and normal conditions (PCC 0.94–0.96 and 0.92–0.96, respectively), whereas *B. distachyon* showed remarkable changes in its transcriptome (PCC 0.84–0.89) reflecting the severe decrease in its biomass (Figures 1B and 5A). Specifically, genes involved in primary metabolism, such as photosynthesis and metabolite and energy generation, were significantly less represented in *B. distachyon* than in *B. stacei* and in *B. hybridum* under long-term heat stress (Figures 5B and C and Additional file 2: Tables S7 and S8), which likely indicates the physiological sensitivity of its cellular system to heat stress. The number of Bs-homoeologs that were expressed more abundantly than Bd-homoeologs was reduced in homoeolog groups

that were highly expressed in *B. hybridum* than in *B. distachyon* at 15 days after exposure to heat stress compared to that found after 3 days of heat stress exposure (Figure 4B and Additional file 1: Figure S8), which suggests that Bs-homoeologs are significantly activated at the earlier phase of transcriptional response to heat stress. We also found significantly higher expression of Bs-homoeologs and genes in *B. stacei* encoding A2-type heat shock transcription factor (*HsfA2*) as well as putative *HsfA2*-targeted genes such as heat shock protein 101 (*Hsp101*) and ascorbate peroxidase 2-like (*APX2*-like; homologs of *Arabidopsis APX1* and rice *APX2*), which are known as key factors in response to heat [44-46], comparing to expression of their counterparts in *B. distachyon* at 3 days after exposure to 32°C (Additional file 1: Figure S9). To validate the homoeolog-specific gene expression in *B. hybridum* observed in our RNA-Seq data, we quantified the polymorphic nucleotides in the cDNA amplicons of *B. hybridum* by using a TaqMan SNP Genotyping Assay in combination with a digital PCR system. The assay discriminates between two homoeo-alleles of a specific SNP that are labeled with two fluorescent dyes (FAM and VIC) and quantifies the expression of each allele by detecting the fluorescence. Specifically, we assessed the quantitative distribution of the decisive SNPs in the two homoeolog groups corresponding to the genes annotated in the Bd21 genome; Bradi1g16510 (*APX2*-like) and Bradi2g49660 (*Hsp101*). We found that the proportions of the SNPs in the cDNA amplicons from the *B. hybridum* RNAs sampled after 3 days of exposure to 32°C were comparable to their expression bias observed in the RNA-Seq analysis (Additional file 1: Figures S10 and S11). These results suggested that the earlier response to heat stress that was specific to the Bs-homoeologs likely contributed to the maintenance

of their cellular homeostasis under the long heat exposure.

Discussion

Evolutionary non-additive gene expression in the hybrid grass *B. hybridum* likely underlies its enhanced invasive behavior

Our transcriptome analysis provided comprehensive evidence for non-additive gene expression in the hybrid species *B. hybridum*, suggesting the global transcriptional changes in its leaves and roots evolved through allopolyploidization. The GO analysis of non-additively expressed homoeolog groups in leaf and root tissues of *B. hybridum* relative to both ancestral species showed an overrepresentation of genes that were involved in the response to stimulus and abiotic stimulus (Additional file 2: Tables S9 and S10). Such functions were also enriched in the homoeolog groups in leaf tissues of *B. hybridum* that showed non-additive expression under heat stress condition (Additional file 2: Tables S11 and S12), which suggests that increased expression divergence of genes related to such functions enhanced this species' ability to respond to environmental change and to adapt to ecological niches. Increased expression divergence of these classes of genes has also been reported in allotetraploid *Arabidopsis* and wheat [24-26]. Although such examples have been reported in few species, future progress in transcriptome datasets of hybrid species and their ancestors might enable the application of universal rules for determining transcriptional changes when new hybrid species are generated. Allopolyploid species have long been hypothesized to possess greater environmental adaptability

to wider niches than their ancestors. Specifically, enhanced heterozygosity and genetic diversity resulting from the hybridization of multiple diverged genomes have been thought to upgrade stress tolerances and contribute to the expansion of niches in hybrid species [47-49]. Although *B. hybridum* shows the largest niche overlap compared with its diploid ancestral species, it shows a niche breadth that is smaller than that of *B. distachyon* and slightly greater than that of *B. stacei* [37, 38]. However, *B. hybridum* also successfully colonized non-native regions of the world [37], which suggests it has greater ecological tolerance than other diploids. With the non-additive transcriptional changes in *B. hybridum*, the expanded diversity of gene expression might contribute to the colonization of non-native areas while avoiding inbreeding depression, and might boost its diversifying selection [50, 51].

Homoeolog-specific gene expression underlies acclimation to heat stress

Although no differences in visible traits were found between the three *Brachypodium* species after 3 days of exposure to heat stress, significant differences in physiological traits in the early stage of heat exposure were revealed from their transcriptomes. The comprehensive list of Bs-homoeolog and *B. stacei* genes with significantly higher expression than the *B. distachyon* genes after 3-day exposure to 32°C included genes involved in the regulation of acclimation to heat in plants, such as heat shock transcription factor, heat shock protein, and DNAJ [52-55] (1,791 genes in Figure 4C, Additional file 2: Table S13), Thus, the functions of *B. hybridum* in acclimating to heat are likely specifically inherited from *B. stacei* via allopolyploidization.

Based on our transcriptome analysis using the *Brachypodium* trio, we hypothesize that the transcriptional response of Bs-homoeologs in *B. hybridum* and their counterparts in *B. stacei* at the early phase during heat exposure contribute to the maintenance of their physiological equilibrium under long-term heat exposure, and might presumably be associated with their heat stress tolerance (Figure 6). Previous evolutional and ecological studies suggested that *B. distachyon* could have adapted to different environments by diverging from *B. stacei* [37, 38]. The distribution areas of *B. distachyon* and *B. stacei* suggested that the two *Brachypodium* species have adapted to cooler and wetter areas and to warmer and drier areas, respectively, via diversification [37]. Therefore, the heat acclimation function in *B. distachyon* might have been lost during its adaptation process after branching from the common ancestor of these diploids. The heat-adaptive trait in the *B. stacei* genome could influence the survival of both individual plants and hybrid progeny under heat stress conditions.

Methods

Fresh weight measurement

The aboveground parts of plants of each species grown under normal and heat stress conditions for 3 or 15 days were used to measure fresh weight. Twelve individuals were used for the measurement. A *t*-test was used for statistical comparisons between the plants grown under the different conditions. The significance threshold was set at $P < 0.01$.

Coverage calculation and SNP calling

To obtain high-quality reads, the genome sequence reads were trimmed by cutting bases off from the start and end of reads if quality ≤20, and removing the reads with final read length <50 using Trimmomatic v.0.32 (Trimmomatic, RRID:SCR_011848) [56] with the -thread 2 LEADING: 20 TRAILING: 20 MINLEN: 50 commands. The trimmed reads were mapped to the reference genome sequence of Bd21 downloaded from Phytozome [57] using TMAP (v.3.1.4; Life Technologies Japan, Tokyo, Japan) with mapall -n 4 -v -Y -u -o 2 stage1 map4 commands. The coverage of genome sequence reads on the reference genome was calculated by removing the non-mapped reads and merging multiple mapping data of the same species from the raw mapping data using SAMTools v.0.1.19 (SAMTools, RRID:SCR_002105) [58] with the view -F 4 and merge commands. The merged data were subjected to coverage calculation using the genomeCoverageBed in BEDTools v.2.20.1 (BEDTools, RRID:SCR_006646) [59] with the default setting. SNPs between the genome sequence reads and reference genome were called by removing the non-mapped reads and possible duplicate reads and merging the multiple mapping data of the same species from the raw mapping data using SAMTools with the view -F 4, rmdup, and merge commands. The merged data were subjected to SNP calling using VarScan pileup2snp (v.2.3.7) [60] with the default settings (minimum read depth to make a call is 8 and minimum supporting reads to call variants is 2), except –p-value 0.01 commands.

Homoeologous SNP identification and virtual *B. stacei* genome construction

Common homogenic SNPs between *B. distachyon* and *B. stacei* and heterogenic SNPs between *B. distachyon* and *B. hybridum* were identified as homoeologous SNPs (Additional file 3); these were used to identify homoeologous genomes in *B. hybridum*. The homoeolog-specific SNPs and their impact to the transcripts and deduced protein sequences were predicted using SnpEff (v.4.2) [61] with the gene structural annotation of Bd21 retrieved from Phytozome (Bdistachyon_314_v3.1.gene_exons.gff3.gz) [57] and -ud 0 command. A virtual *B. stacei* genome was constructed by replacing the nucleotides of the homoeologous SNPs in the *B. distachyon* genome with those in the *B. stacei* genome using an original Perl script (Additional file 4).

Ka* and *Ks* calculation and estimation of the speciation time of *B. distachyon* and *B. stacei

The *Ka* and *Ks* values between genes of homoeolog groups were calculated with Ka_Ks Calculator (v.2.0) [62] using a modified version that implements the Yang-Nielsen algorithm (MYN) method [63]. The speciation time (T) based on the *Ks* value was estimated by using the equation $T = Ks/2\lambda$ where λ = 6.1 × 10-9 [64, 65], based on the average *Ks* value of each homoeolog group.

Read count and reads per million calculations

To obtain high-quality reads, the RNA-Seq reads were trimmed by cutting bases off from the start and end of reads if quality ≤20, and removing the reads with final read length <50 using Trimmomatic with -thread 4

LEADING: 20 TRAILING: 20 MINLEN: 50 commands. The trimmed reads were mapped to Bd21 and the virtual *B. stacei* genome using TMAP with mapall -n 4 -v -Y -u -o 2 stage1 map4 commands; *B. distachyon* reads were mapped to the Bd21 genome, *B. stacei* reads were mapped to the virtual *B. stacei* genome, and *B. hybridum* reads were mapped to both these genomes. The expression levels of the homoeologs in *B. hybridum* were quantified by classifying the RNA-Seq reads into the following three groups based on the number of mismatches between read and both genomes using original Perl script (Additional file 5): Bd-subgenome origin reads, Bs-genome origin reads, and unclassified reads (Additional file 1; Figure S5B). The mapping data from the *B. distachyon* reads, *B. stacei* reads, Bd-subgenome origin reads, and Bs-genome origin reads were subjected to read count using featureCounts (v.1.4.6) [66] with the gene structural annotation of Bd21. As the MAV, which is the average of the expression values in both ancestors, the total read count data from the *B. distachyon* and *B. stacei* reads were used, and as the entire expression value of the homoeolog group in *B. hybridum*, the total read count data for the Bd- and the Bs-subgenome origin reads were used (Additional file 1: Figure S5A). The RPM values were calculated for all annotated genes based on the read count data. Genes, MAVs, homoeologs, and homoeolog groups with RPM $\geq$ 1 in all three biological replicates were defined as expressed.

Validation of homoeolog-specific gene expression in *B. hybridum*

The ratios of expression between homoeologs were verified using a digital PCR system in combination with

the TaqMan® SNP Genotyping Assay method (Applied Biosystems, CA, USA) [67]. PCR primers were designed at conserved regions between homeologs, and targeted homoelogous SNP was included in the amplified region. Two dye-labeled (FAM and VIC) TaqMan probes were designed for quantifying homoeologous SNPs using the TaqMan MGB SNP Kit (Applied Biosystems). cDNA was prepared from *B. hybridum* RNA sampled after 3-day exposure to heat stress (with three biological replicates) using the SuperScript® IV Reverse Transcriptase (Invitrogen, CA, USA), and was used for quantitative PCR. The thermocycling conditions were initiated at 96°C for 10 min, followed by 39 cycles of annealing and extension at 56 °C for 2 min and denaturation at 98°C for 30 sec, followed by a final extension at 56°C for 2 min. Homoeologous SNPs in the cDNA amplicons were detected with QuantStudio™ 3D (Life Technologies Japan). The PCR primers and TaqMan probes used in this assay are listed in Additional file 2: Table S14.

Differentially expressed genes analysis

Differentially expressed genes between the three *Brachypodium* species, homoeolog groups, and growth conditions were identified using the DESeq2 package (v.1.10.1) [68] in R (v.3.2.4) with the Wald test based on the read count data. FDR for each comparison was calculated by adjusting the *p*-value using the Benjamini-Hochberg procedure. Genes with an FDR ≤ 0.001 were defined as differentially expressed.

GO enrichment analysis

GO annotations of the *B. distachyon* genes were prepared by the method used in Koda et al. (2017) [69]. GO terms for the *B. distachyon* genes were used from the annotation information retrieved from Phytozome (Bdistachyon_314_v3.1.annotation_info.txt) [57]. Additional GO terms were associated with the *B. distachyon* genes based on GO terms of transcripts for *A. thaliana* and rice in the "Best-hit-arabi-name" and "Best-hit-rice-name" row in the annotation file. The GO terms for genes annotated in *A. thaliana* and rice were used from the annotation information downloaded from Phytozome (Athaliana_167_TAIR10.annotation_info.txt, [70], and Osativa_204_v7.0.annotation_info.txt, [71]). To reduce bias, GO terms assigned to more than 3000 genes in *B. distachyon* were excluded. Enriched GO terms for selected genes were identified using BLAST2GO (v.3.3.5) [72] with the Fisher's exact test. All of the annotated genes in the Bd21 genome were used as a reference set. The enriched GO terms were summarized using the web-based tool REVIGO [73] if more than 10 enriched GO terms were found. GO annotations of the *B. distachyon* genes used in this study are provided in Additional file 6.

Availability of supporting data

All sequencing data were archived at DDBJ under the accession numbers DRA005717 and DRA005699 (BioProject accessions: PRJDB5654 and PRJDB5657). Supporting data are available in the *GigaScience* repository, GigaDB [74] and via additional files. Additional files 1 and 2 provide Supplementary Figures and

Supplementary Tables, respectively. Additional file 3 provides the homoeologous SNP dataset used in this study. Additional files 4 and 5 provide the original Perl script codes used in this study. Additional file 6 provides the GO annotations of the *B. distachyon* genes used in this study. The protocol for detection of allele frequencies in the cDNA sample is available via protocols.io [75].

Additional files

Additional file 1: Supplementary Figures

Figure S1. Phylogenetic relationships among the three *Brachypodium* species.

Figure S2. Results of homoeologous SNP identification.

Figure S3. Overview of genomic sequence data analysis.

Figure S4. Location and effect of homoeologous SNPs.

Figure S5. Overview of RNA-Seq data analysis.

Figure S6. Classification of the read origin of the *B. hybridum* RNA reads from leaf and root tissues.

Figure S7. Log_2 fold-change distribution of homoeolog expression in *B. hybridum* under heat stress condition for 3 days.

Figure S8. Gene expression profiles of the Bd- and Bs-homoeologs in homoeolog groups showing significantly higher expression in *B. hybridum* than in *B. distachyon* under heat stress condition for 15 days.

Figure S9. Gene expression profiles of the *Brachypodium HsfA2* and putative *HsfA2*-targeted genes at 3 days after heat stress exposure.

Figure S10. Quantitative detection of fluorescence of homoeologs in *B. hybridum*.

Figure S11. Expression ratios of Bd- and Bs-homoeologs based on RNA-Seq analysis and TaqMan SNP Genotyping Assay.

Additional file 2: Supplementary Tables

Table S1. Summary of the sequencing analysis and accession numbers

Table S2. Summary of the whole genome sequencing analysis and mapping results

Table S3. Results of SNP calling

Table S4. Numbers of variant effects in genes

Table S5. Enriched GO terms in the biological process ontology of homoeolog groups showing transgressive expression in *B. hybridum* leaf under normal condition

Table S6. Enriched GO terms in the biological process ontology of homoeolog groups showing transgressive expression in *B. hybridum* root under normal condition

Table S7. Enriched GO terms in the biological process ontology of genes showing significantly higher expression in *B. stacei* than in *B. distachyon* at 15 days after heat stress exposure

Table S8. Enriched GO terms in the biological process ontology of homoeolog groups showing significantly higher expression in *B. hybridum* than in *B. distachyon* at 15 days after heat stress exposure

Table S9. Enriched GO terms in the biological process ontology of homoeolog groups showing non-additive expression in *B. hybridum* leaf under normal condition

Table S10. Enriched GO terms in the biological process ontology of homoeolog groups showing non-additive expression in *B. hybridum* root under normal condition

Table S11. Enriched GO terms in the biological process ontology of homoeolog groups showing non-additive expression in *B. hybridum* leaf at 3 days after heat stress exposure

Table S12. Enriched GO terms in the biological process ontology of homoeolog groups showing non-additive expression in *B. hybridum* leaf at 15 days after heat stress exposure

Table S13. Genes showing higher expression in the *B. stacei* genome than in the *B. distachyon* genome in the three *Brachypodium* species at 3 days after heat stress exposure

Table S14. Amplicon primer and TaqMan probes used in this study

Additional file 3: Homoeologous SNP dataset used in this study

Additional file 4: Original Perl script code used to construct the virtual *B. stacei* genome by replacing the nucleotides of the homoeologous SNPs in the *B. distachyon* genome with those in the *B. stacei* genome

Additional file 5: Original Perl script code used to classify the RNA-Seq reads of *B. hybridum* into the

Bd-subgenome origin reads, Bs-subgenome origin reads, and unclassified reads

Additional file 6: GO annotations of the *B. distachyon* genes used in this study

Abbreviations

DNA, Deoxyribonucleic acid; FDR, False discovery rate; GO, Gene ontology; MAV, Mid-ancestral value; MAY, Million years ago; PCC, Pearson's correlation coefficient; RNA, Ribonucleic acid; RPM, Reads per million mapped reads; SNP, Single nucleotide polymorphism; UTR, Untranslated region

Competing interests

The authors declare that they have no competing interests.

Author contributions

KT and KM designed the work. KT and YO grew and sampled the plants. MS, YU-Y and YO generated the genome sequencing data. KT and KI generated the RNA-Seq data. KT performed in silico analyses using the genome and RNA-Seq data. KM directed this study. KT and KM wrote the manuscript with all authors.

Acknowledgments

The authors thank the National Plant Germplasm System of USDA-ARS, David F. Garvin and Pilar Catalán

for providing *Brachypodium* seeds. This work was partially supported by grants-in-aid for Young Scientists (A) (grant no. 26712003 to KM) from the Japan Society for the Promotion of Science (JSPS) and by funds to KM from the Advanced Low Carbon Technology Research and Development Program (ALCA, J2013403) of the Japan Science and Technology Agency (JST). This work was also supported by RIKEN Junior Research Associate Program.

References

1. Soltis PS and Soltis DE. Polyploidy and genome evolution. Berlin, New York: Springer Verlag; 2012.
2. Comai L. The advantages and disadvantages of being polyploid. Nat Rev Genet. 2005;6 11:836-46.
3. Leitch AR and Leitch IJ. Perspective - Genomic plasticity and the diversity of polyploid plants. Science. 2008;320 5875:481-3.
4. Bowman JL, Floyd SK and Sakakibara K. Green genes-comparative genomics of the green branch of life. Cell. 2007;129 2:229-34.
5. Adams KL and Wendel JF. Polyploidy and genome evolution in plants. Curr Opin Plant Biol. 2005;8 2:135-41.
6. Segraves KA. The effects of genome duplications in a community context. New Phytol. 2017.
7. Bowers JE, Chapman BA, Rong J and Paterson AH. Unravelling angiosperm genome evolution by phylogenetic analysis of chromosomal duplication events. Nature. 2003;422 6930:433-8.

8. Otto SP. The evolutionary consequences of polyploidy. Cell. 2007;131 3:452-62.

9. Jiao Y, Wickett NJ, Ayyampalayam S, Chanderbali AS, Landherr L, Ralph PE, et al. Ancestral polyploidy in seed plants and angiosperms. Nature. 2011;473 7345:97-100.

10. Soltis PS and Soltis DE. The role of hybridization in plant speciation. Annu Rev Plant Biol. 2009;60:561-88.

11. Fawcett JA, Maere S and Van de Peer Y. Plants with double genomes might have had a better chance to survive the Cretaceous-Tertiary extinction event. Proc Natl Acad Sci U S A. 2009;106 14:5737-42.

12. Vanneste K, Baele G, Maere S and Van de Peer Y. Analysis of 41 plant genomes supports a wave of successful genome duplications in association with the Cretaceous-Paleogene boundary. Genome Res. 2014;24 8:1334-47.

13. Chen ZJ. Molecular mechanisms of polyploidy and hybrid vigor. Trends Plant Sci. 2010;15 2:57-71.

14. Chen ZJ. Genomic and epigenetic insights into the molecular bases of heterosis. Nat Rev Genet. 2013;14 7:471-82.

15. Chen ZJ and Birchler JA. Polyploid and hybrid genomics. Ames, Iowa: Wiley-Blackwell; 2013.

16. Solhaug EM, Ihinger J, Jost M, Gamboa V, Marchant B, Bradford D, et al. Environmental Regulation of Heterosis in the Allopolyploid Arabidopsis suecica. Plant Physiol. 2016;170 4:2251-63.

17. Maricle BR, Crosier JJ, Bussiere BC and Lee RW. Respiratory enzyme activities correlate with

anoxia tolerance in salt marsh grasses. J Exp Mar Bio Ecol. 2006;337 1:30-7.

18. Ainouche ML, Fortune PM, Salmon A, Parisod C, Grandbastien MA, Fukunaga K, et al. Hybridization, polyploidy and invasion: lessons from Spartina (Poaceae). Biol Invasions. 2009;11 5:1159-73.

19. Dubcovsky J and Dvorak J. Genome plasticity a key factor in the success of polyploid wheat under domestication. Science. 2007;316 5833:1862-6.

20. Yang C, Zhao L, Zhang H, Yang Z, Wang H, Wen S, et al. Evolution of physiological responses to salt stress in hexaploid wheat. Proc Natl Acad Sci U S A. 2014;111 32:11882-7.

21. Stebbins GL. Variation and evolution in plants. New York: Columbia University Press; 1950.

22. Stebbins GL. Chromosomal evolution in higher plants. Reading, Massachusetts: Addison-Wesley; 1971.

23. Yoo MJ, Liu X, Pires JC, Soltis PS and Soltis DE. Nonadditive gene expression in polyploids. Annu Rev Genet. 2014;48:485-517.

24. Wang J, Tian L, Lee HS, Wei NE, Jiang H, Watson B, et al. Genomewide nonadditive gene regulation in Arabidopsis allotetraploids. Genetics. 2006;172 1:507-17.

25. Chague V, Just J, Mestiri I, Balzergue S, Tanguy AM, Huneau C, et al. Genome-wide gene expression changes in genetically stable synthetic and natural wheat allohexaploids. New Phytol. 2010;187 4:1181-94.

26. Kim ED and Chen ZJ. Unstable transcripts in Arabidopsis allotetraploids are associated with nonadditive gene expression in response to abiotic and biotic stresses. PLoS One. 2011;6 8:e24251.

27. Grover CE, Gallagher JP, Szadkowski EP, Yoo MJ, Flagel LE and Wendel JF. Homoeolog expression bias and expression level dominance in allopolyploids. New Phytol. 2012;196 4:966-71.

28. Madlung A. Polyploidy and its effect on evolutionary success: old questions revisited with new tools. Heredity (Edinb). 2013;110 2:99-104.

29. Yoo MJ, Szadkowski E and Wendel JF. Homoeolog expression bias and expression level dominance in allopolyploid cotton. Heredity (Edinb). 2013;110 2:171-80.

30. Zhang D, Pan Q, Tan C, Zhu B, Ge X, Shao Y, et al. Genome-Wide Gene Expressions Respond Differently to A-subgenome Origins in Brassica napus Synthetic Hybrids and Natural Allotetraploid. Front Plant Sci. 2016;7:1508.

31. Bardil A, de Almeida JD, Combes MC, Lashermes P and Bertrand B. Genomic expression dominance in the natural allopolyploid Coffea arabica is massively affected by growth temperature. New Phytol. 2011;192 3:760-74.

32. Li A, Liu D, Wu J, Zhao X, Hao M, Geng S, et al. mRNA and Small RNA Transcriptomes Reveal Insights into Dynamic Homoeolog Regulation of Allopolyploid Heterosis in Nascent Hexaploid Wheat. Plant Cell. 2014;26 5:1878-900.

33. Zhang H, Gou X, Zhang A, Wang X, Zhao N, Dong Y, et al. Transcriptome shock invokes disruption

of parental expression-conserved genes in tetraploid wheat. Sci Rep. 2016;6:26363.

34. Akama S, Shimizu-Inatsugi R, Shimizu KK and Sese J. Genome-wide quantification of homeolog expression ratio revealed nonstochastic gene regulation in synthetic allopolyploid Arabidopsis. Nucleic Acids Res. 2014;42 6:e46.

35. Paape T, Hatakeyama M, Shimizu-Inatsugi R, Cereghetti T, Onda Y, Kenta T, et al. Conserved but Attenuated Parental Gene Expression in Allopolyploids: Constitutive Zinc Hyperaccumulation in the Allotetraploid Arabidopsis kamchatica. Mol Biol Evol. 2016;33 11:2781-800.

36. Lopez-Alvarez D, Lopez-Herranz ML, Betekhtin A and Catalan P. A DNA barcoding method to discriminate between the model plant Brachypodium distachyon and its close relatives B. stacei and B. hybridum (Poaceae). PLoS One. 2012;7 12:e51058.

37. Lopez-Alvarez D, Manzaneda AJ, Rey PJ, Giraldo P, Benavente E, Allainguillaume J, et al. Environmental niche variation and evolutionary diversification of the Brachypodium distachyon grass complex species in their native circum-Mediterranean range. Am J Bot. 2015;102 7:1073-88.

38. Catalan P, Muller J, Hasterok R, Jenkins G, Mur LA, Langdon T, et al. Evolution and taxonomic split of the model grass Brachypodium distachyon. Ann Bot. 2012;109 2:385-405.

39. Catalan P, Chalhoub B, Chochois V, Garvin DF, Hasterok R, Manzaneda AJ, et al. Update on the genomics and basic biology of Brachypodium International Brachypodium Initiative (IBI). Trends Plant Sci. 2014;19 7:414-8.

40. Catalan P, Lopez-Alvarez D, Bellosta C and Villar L. Updated taxonomic descriptions, iconography, and habitat preferences of Brachypodium distachyon, B-stacei, and B-hybridum (Poaceae). An Jardin Bot Madrid. 2016;73 1.

41. Betekhtin A, Jenkins G and Hasterok R. Reconstructing the Evolution of Brachypodium Genomes Using Comparative Chromosome Painting. PLoS One. 2014;9 12:e115108.

42. Vogel J. Genetics and genomics of Brachypodium. New York, New York: Springer Science+Business Media; 2016.

43. Dinh Thi VH, Coriton O, Le Clainche I, Arnaud D, Gordon SP, Linc G, et al. Recreating Stable Brachypodium hybridum Allotetraploids by Uniting the Divergent Genomes of B. distachyon and B. stacei. PLoS One. 2016;11 12:e0167171.

44. Schramm F, Ganguli A, Kiehlmann E, Englich G, Walch D and von Koskull-Doring P. The heat stress transcription factor HsfA2 serves as a regulatory amplifier of a subset of genes in the heat stress response in Arabidopsis. Plant Mol Biol. 2006;60 5:759-72.

45. Charng YY, Liu HC, Liu NY, Chi WT, Wang CN, Chang SH, et al. A heat-inducible transcription factor, HsfA2, is required for extension of acquired thermotolerance in Arabidopsis. Plant Physiol. 2007;143 1:251-62.

46. Chauhan H, Khurana N, Agarwal P and Khurana P. Heat shock factors in rice (Oryza sativa L.): genome-wide expression analysis during reproductive development and abiotic stress. Mol Genet

Genomics. 2011;286 2:171-87.

47. Lowry E and Lester SE. The biogeography of plant reproduction: potential determinants of species' range sizes. J Biogeogr. 2006;33 11:1975-82.

48. te Beest M, Le Roux JJ, Richardson DM, Brysting AK, Suda J, Kubesova M, et al. The more the better? The role of polyploidy in facilitating plant invasions. Ann Bot. 2012;109 1:19-45.

49. Marchant DB, Soltis DE and Soltis PS. Patterns of abiotic niche shifts in allopolyploids relative to their progenitors. New Phytol. 2016;212 3:708-18.

50. Bakker EG, Montgomery B, Nguyen T, Eide K, Chang J, Mockler TC, et al. Strong population structure characterizes weediness gene evolution in the invasive grass species Brachypodium distachyon. Mol Ecol. 2009;18 12:2588-601.

51. Meimberg H, Rice KJ, Milan NF, Njoku CC and McKay JK. Multiple origins promote the ecological amplitude of allopolyploid Aegilops (Poaceae). Am J Bot. 2009;96 7:1262-73.

52. Song LL, Jiang YL, Zhao HQ and Hou MF. Acquired thermotolerance in plants. Plant Cell Tiss Org. 2012;111 3:265-76.

53. Driedonks N, Xu J, Peters JL, Park S and Rieu I. Multi-Level Interactions Between Heat Shock Factors, Heat Shock Proteins, and the Redox System Regulate Acclimation to Heat. Front Plant Sci. 2015;6:999.

54. Jacob P, Hirt H and Bendahmane A. The heat-shock protein/chaperone network and multiple stress

resistance. Plant Biotechnol J. 2017;15 4:405-14.

55. Ohama N, Sato H, Shinozaki K and Yamaguchi-Shinozaki K. Transcriptional Regulatory Network of Plant Heat Stress Response. Trends Plant Sci. 2017;22 1:53-65.

56. Bolger AM, Lohse M and Usadel B. Trimmomatic: a flexible trimmer for Illumina sequence data. Bioinformatics. 2014;30 15:2114-20.

57. Phytozome (Brachypodium distachyon v3.1). http://genome.jgi.doe.gov/pages/dynamicOrganismDownload.jsf?organism=Bdistachyon. Accessed 19 JUL 2016.

58. Li H, Handsaker B, Wysoker A, Fennell T, Ruan J, Homer N, et al. The Sequence Alignment/Map format and SAMtools. Bioinformatics. 2009;25 16:2078-9.

59. Quinlan AR. BEDTools: The Swiss-Army Tool for Genome Feature Analysis. Curr Protoc Bioinformatics. 2014;47:11 2 1- 2 34.

60. Koboldt DC, Chen K, Wylie T, Larson DE, McLellan MD, Mardis ER, et al. VarScan: variant detection in massively parallel sequencing of individual and pooled samples. Bioinformatics. 2009;25 17:2283-5.

61. Cingolani P, Platts A, Wang le L, Coon M, Nguyen T, Wang L, et al. A program for annotating and predicting the effects of single nucleotide polymorphisms, SnpEff: SNPs in the genome of Drosophila melanogaster strain w1118; iso-2; iso-3. Fly (Austin). 2012;6 2:80-92.

62. Wang D, Zhang Y, Zhang Z, Zhu J and Yu J. KaKs_Calculator 2.0: a toolkit incorporating gamma-series methods and sliding window strategies. Genomics Proteomics Bioinformatics. 2010;8 1:77-80.

63. Zhang Z, Li J and Yu J. Computing Ka and Ks with a consideration of unequal transitional substitutions. BMC Evol Biol. 2006;6:44.

64. Lynch M and Conery JS. The evolutionary fate and consequences of duplicate genes. Science. 2000;290 5494:1151-5.

65. He N, Zhang C, Qi X, Zhao S, Tao Y, Yang G, et al. Draft genome sequence of the mulberry tree Morus notabilis. Nat Commun. 2013;4:2445.

66. Liao Y, Smyth GK and Shi W. featureCounts: an efficient general purpose program for assigning sequence reads to genomic features. Bioinformatics. 2014;30 7:923-30.

67. Schleinitz D, Distefano JK and Kovacs P. Targeted SNP genotyping using the TaqMan(R) assay. Methods Mol Biol. 2011;700:77-87.

68. Love MI, Huber W and Anders S. Moderated estimation of fold change and dispersion for RNA-seq data with DESeq2. Genome Biol. 2014;15 12:550.

69. Koda S, Onda Y, Matsui H, Takahagi K, Uehara-Yamaguchi Y, Shimizu M, et al. Diurnal Transcriptome and Gene Network Represented through Sparse Modeling in Brachypodium distachyon. Front Plant Sci. 2017;8:2055.

70. Phytozome (Arabidopsis thaliana TAIR10). http://genome.jgi.doe.gov/pages/dynamicOrganismDownload.jsf?organism=Athaliana. Accessed 12 OCT 2016.

71. Phytozome (Oryza sativa v7_JGI). http://genome.jgi.doe.gov/pages/dynamicOrganismDownload.jsf?organism=Osativa. Accessed 12 OCT 2016.

72. Conesa A, Gotz S, Garcia-Gomez JM, Terol J, Talon M and Robles M. Blast2GO: a universal tool for annotation, visualization and analysis in functional genomics research. Bioinformatics. 2005;21 18:3674-6.

73. Supek F, Bosnjak M, Skunca N and Smuc T. REVIGO summarizes and visualizes long lists of gene ontology terms. PLoS One. 2011;6 7:e21800.

74. Takahagi K, Inoue K, Shimizu M, Uehara-Yamaguchi Y, Onda Y and Mochida K. Supporting data for "Homoeolog-specific activation of genes for heat acclimation in the allopolyploid grass Brachypodium hybridum". *GigaScience* Database 2018. http://dx.doi.org/10.5524/100413

75 Takahagi K, Inoue K, Shimizu M, Uehara-Yamaguchi Y, Onda Y and Mochida K. Detection of allele frequencies in the cDNA sample. Protocols.io 2018. dx.doi.org/10.17504/protocols.io.nafdabn

Figure legends

Figure 1. Effect of heat stress on the growth of the three *Brachypodium* species.

Fresh weight of *B. distachyon*, *B. stacei*, and *B. hybridum* grown under normal (22°C) and heat stress (32°C) conditions at 3 (**A**) and 15 (**B**) days after stress exposure. Data are the mean value ± standard deviation for twelve individuals. Statistical differences are indicated by an asterisk ($P < 0.01$, *t*-test). Bd, *B. distachyon*; Bs, *B. stacei*; Bh, *B. hybridum*.

Figure 2. Summary of genome sequence data.

Mapping results and polymorphic features of the genomic reads from *B. hybridum* and *B. stacei* superimposed to the reference genome sequence of Bd21 (100-kb sliding windows). (1) Number of homoeologous SNPs. (2) Number of heterogenic SNPs between *B. distachyon* and *B. hybridum*. (3) Average number of *B. hybridum* reads mapped to the reference genome. (4) Number of homogenic SNPs between *B. distachyon* and *B. stacei*. (5) Average number of *B. stacei* reads mapped to the reference genome. (6) Density distribution of the Bd21 annotated genes.

Figure 3. Global gene expression patterns in *B. hybridum* compared with its ancestors.

(**A** and **B**) Venn diagram of genes expressed in the MAV (left) and homoeolog groups expressed in *B. hybridum* (right) in leaf (**A**) and root (**B**) tissues. (**C** and **D**) Additive and non-additive gene expression

patterns in leaf (**C**) and root (**D**) tissues of *B. hybridum*. Genes (homoeolog groups) expressed in both the MAV and *B. hybridum* (17,256 genes (homoeolog groups)) were analyzed. (**E** and **F**) Ancestral and non-ancestral gene expression patterns in leaf (**E**) and root (**F**) tissues in *B. hybridum*. Genes (homoeolog groups) expressed in both the MAV and *B. hybridum* (18,021 genes (homoeolog groups)) were analyzed. “=” indicates that the gene expression level is not significantly different between the compared groups. “>” and “<” indicate that the gene expression level is significantly different between the compared groups. The significance threshold is set at FDR $\leq$ 0.001. Bd, *B. distachyon*; Bs, *B. stacei*; MAV, Mid-ancestral value; BhBd, Bd-homoeologs in *B. hybridum*; BhBs, Bs-homoeologs in *B. hybridum*; Bh, *B. hybridum*.

Figure 4. Heat stress response in *B. hybridum*.

(**A**) Heatmap of Pearson’s correlation coefficients based on the gene expression profiles of each species at 3 days after heat stress exposure. Pearson correlation coefficient values between different conditions are shown in yellow squares. (**B**) Gene expression profiles of the Bd- and Bs-homoeologs in homoeolog groups showing significantly higher expression in *B. hybridum* than in *B. distachyon* under heat stress condition at 3 days after stress exposure. Each dot represents the average expression value of three biological replicates. Blue dots represent genes showing significantly higher expression in Bd-homoeologs than in Bs-homoeologs and red dots represent genes showing significantly higher expression in Bs-homoeologs than in Bd-homoeologs (FDR $\leq$ 0.001). (**C**) Genes showing significantly higher expression in the *B. stacei* genome than in the *B.*

distachyon genome in the three *Brachypodium* species under heat stress condition for 3 days. The left circle represents genes showing significantly higher expression in *B. stacei* than in *B. distachyon*. The right circle represents genes showing significantly higher expression in Bs-homoeologs than in Bd-homoeologs in homoeolog groups showing significantly higher expression in *B. hybridum* than in *B. distachyon*. (**D**) Enriched GO terms in the biological process ontology of 1,791 intersectional genes in the Venn diagram shown in (**C**). Bd, *B. distachyon*; Bs, *B. stacei*; BhBd, Bd-homoeologs in *B. hybridum*; BhBs, Bs-homoeologs in *B. hybridum*; Bh, *B. hybridum*.

Figure 5. Maintenance of transcriptional states in *B. hybridum* and *B. stacei* under long-term heat stress. (**A**) Heatmap of Pearson's correlation coefficients based on the gene expression profiles of each species at 15 days after heat stress exposure. Pearson correlation coefficient values between different conditions are marked by yellow squares. (**B** and **C**) Top 10 enriched GO terms in the biological process ontology of genes showing significantly higher expression in *B. stacei* than in *B. distachyon* (**B**) and homoeolog groups showing significantly higher expression in *B. hybridum* than in *B. distachyon* (**C**) at 15 days after heat stress exposure. All enriched GO terms for these genes and homoeolog groups are shown in additional file 2: Table S7 and S8. Bd, *B. distachyon*; Bs, *B. stacei*; BhBd, Bd-homoeologs in *B. hybridum*; BhBs, Bs-homoeologs in *B. hybridum*; Bh, *B. hybridum*.

Figure 6. Model of phenotype and transcriptome of the three *Brachypodium* species under long-term heat stress.

Bd, *B. distachyon*; Bs, *B. stacei*; BhBd, Bd-homoeologs in *B. hybridum*; BhBs, Bs-homoeologs in *B. hybridum*; Bh, *B. hybridum*.

Figure 1

Click here to download Figure Fig1.pdf

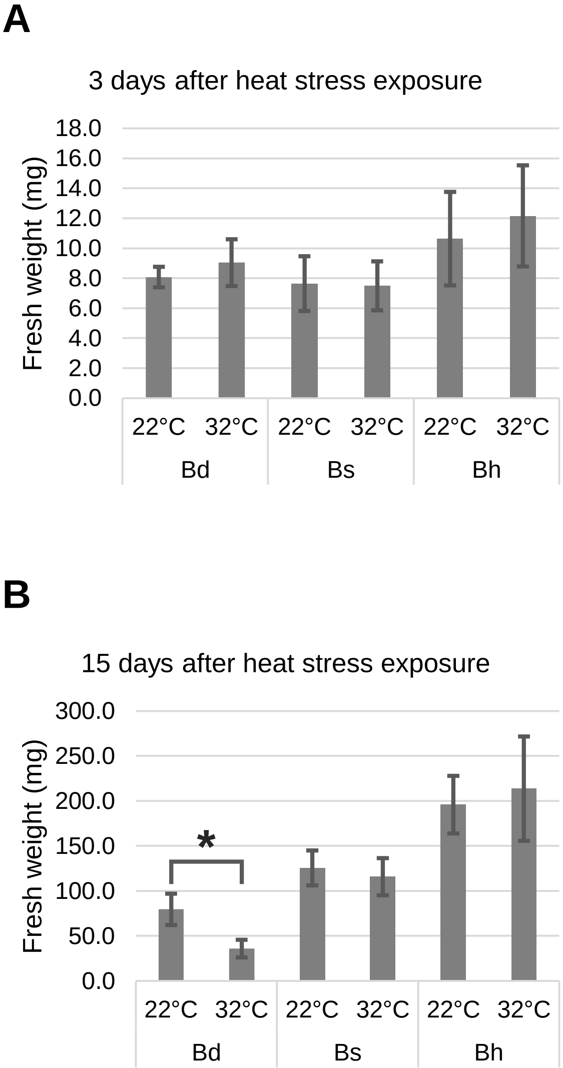

Figure 2

Click here to download Figure Fig2.pdf

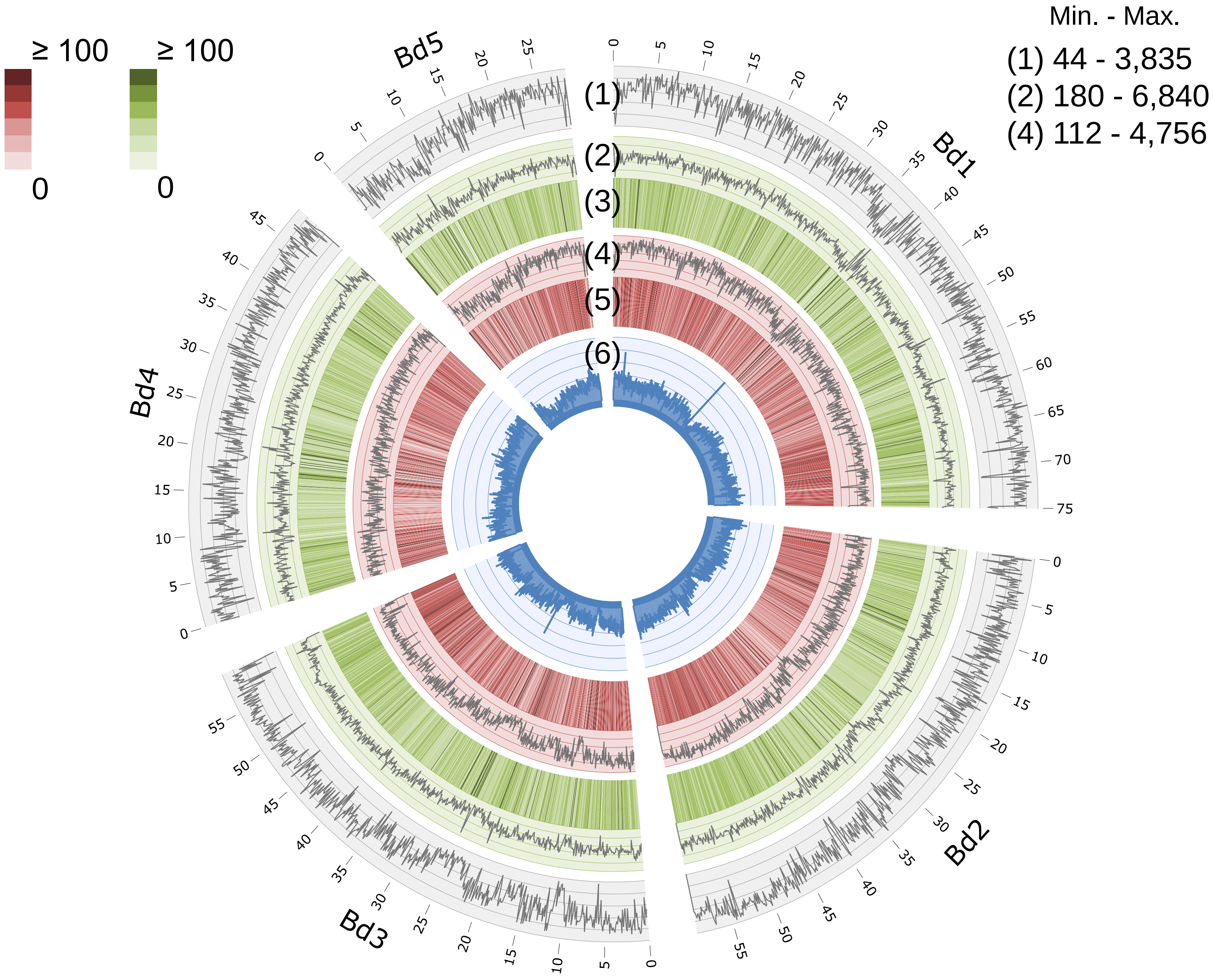

Figure 3

Click here to download Figure Fig3.pdf

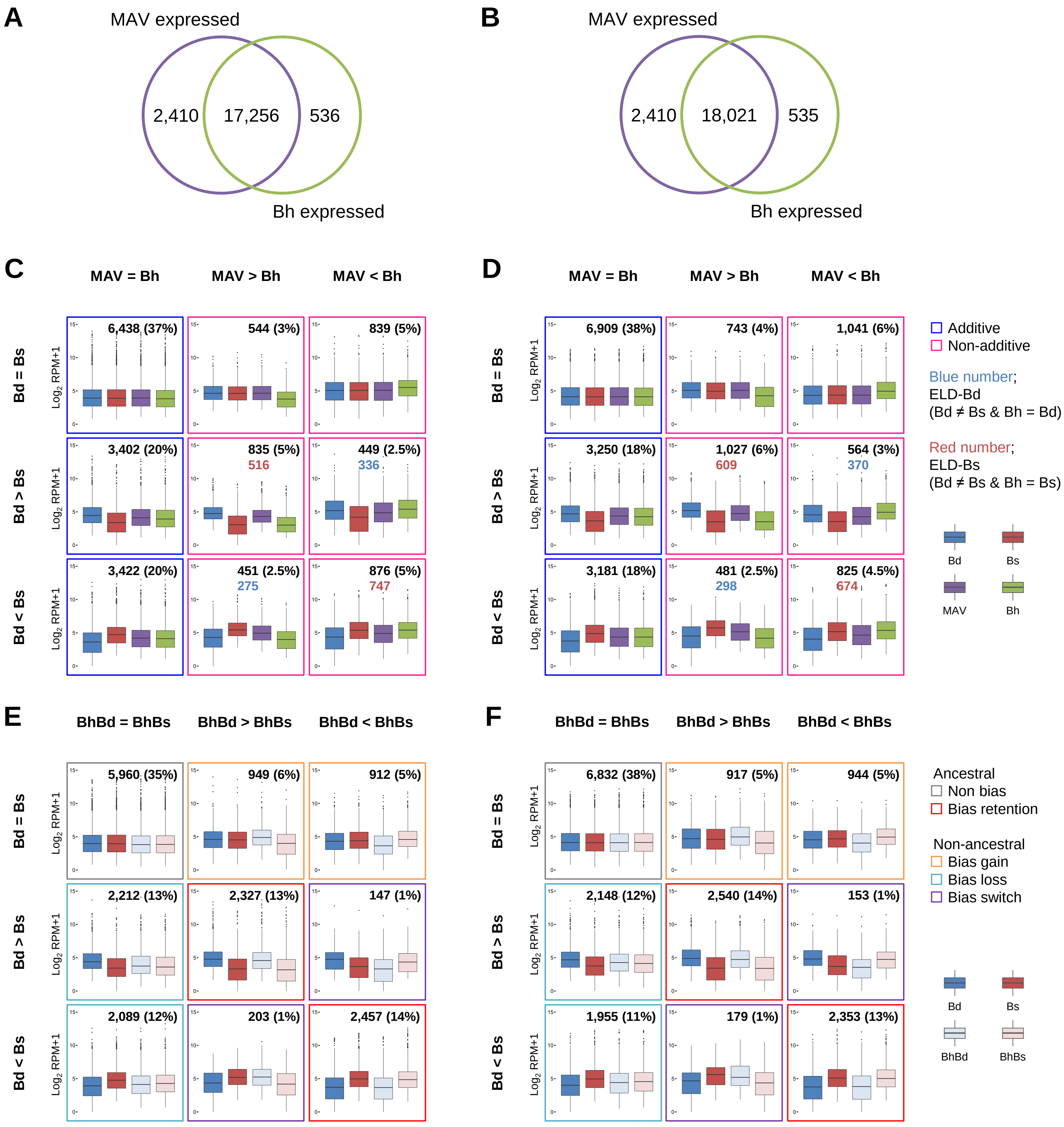

Figure 4

Click here to download Figure Fig4.pdf

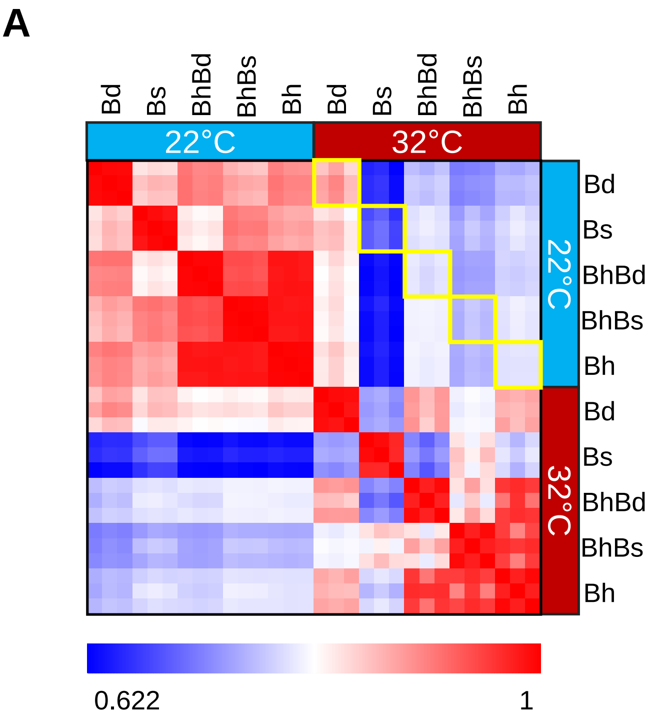

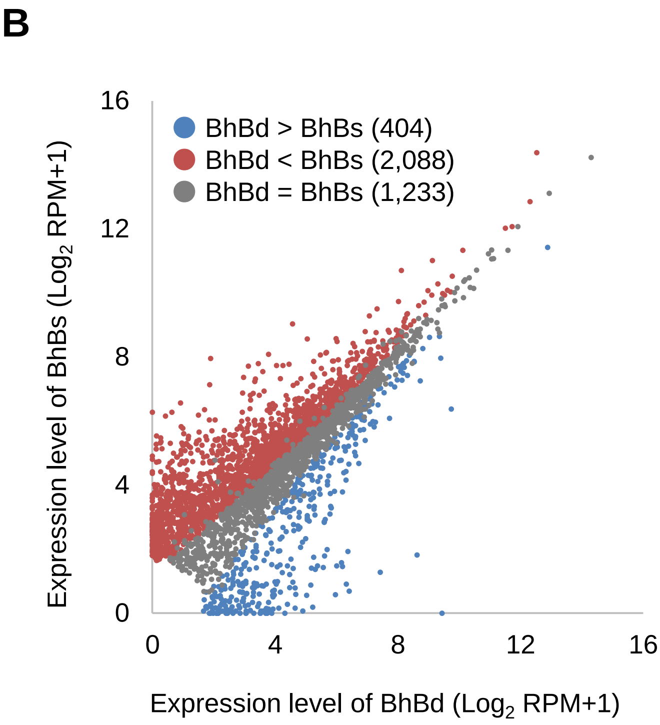

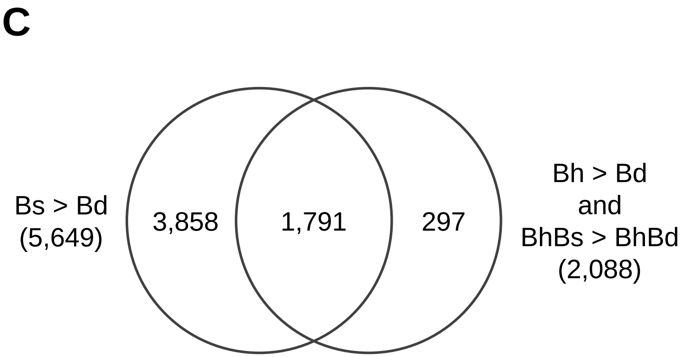

D

Genes showing higher expression in the Bs genome than in the Bd genome in the three *Brachypodium* species (1,791)

GO-ID	Term	FDR
GO:0006259	DNA metabolic process	8.26E-03
GO:0006974	cellular response to DNA damage stimulus	1.99E-02
GO:0006796	phosphate-containing compound metabolic process	2.22E-02
GO:0006793	phosphorus metabolic process	2.29E-02
GO:0033554	cellular response to stress	2.74E-02
GO:0006281	DNA repair	3.02E-02

Figure 5

Click here to download Figure Fig5.pdf

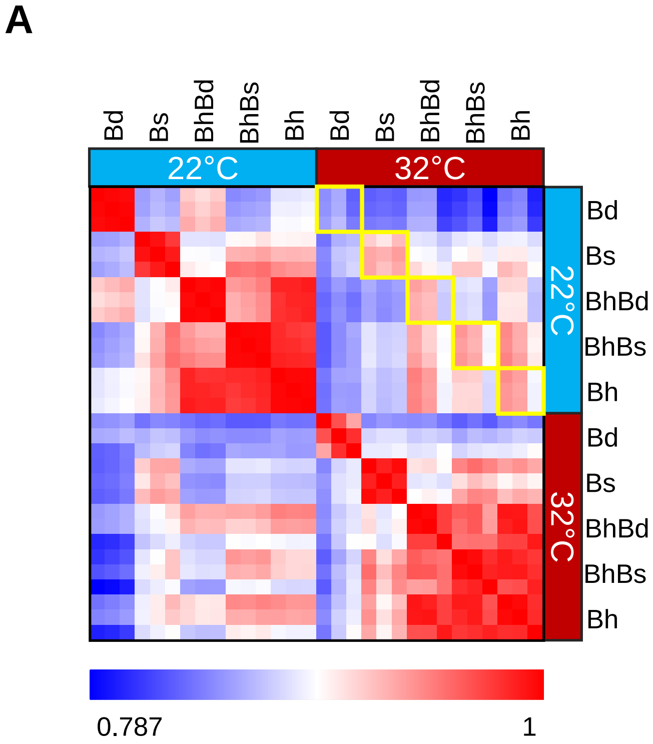

B

Genes showing higher expression in Bs than in Bd (5,517)

GO-ID	Term	FDR
GO:0015979	photosynthesis	1.03E-12
GO:0044710	single-organism metabolic process	3.46E-12
GO:0006091	generation of precursor metabolites and energy	6.73E-09
GO:0044281	small molecule metabolic process	5.69E-07
GO:0044711	single-organism biosynthetic process	6.37E-06
GO:0005975	carbohydrate metabolic process	4.21E-05
GO:0009765	photosynthesis, light harvesting	6.85E-05
GO:0044283	small molecule biosynthetic process	1.35E-04
GO:0006082	organic acid metabolic process	2.44E-04
GO:0051186	cofactor metabolic process	3.85E-04

C

Homoeolog groups showing higher expression in Bh than in Bd (4,009)

GO-ID	Term	FDR
GO:0044710	single-organism metabolic process	4.06E-15
GO:0015979	photosynthesis	2.61E-14
GO:0006091	generation of precursor metabolites and energy	2.63E-14
GO:0044281	small molecule metabolic process	2.94E-08
GO:0044711	single-organism biosynthetic process	6.21E-07
GO:0009765	photosynthesis, light harvesting	1.02E-06
GO:0006629	lipid metabolic process	2.49E-06
GO:0055114	oxidation-reduction process	2.54E-06
GO:0051186	cofactor metabolic process	4.61E-05
GO:0006732	coenzyme metabolic process	8.67E-05

Figure 6 Click here to download Figure Fig6.pdf

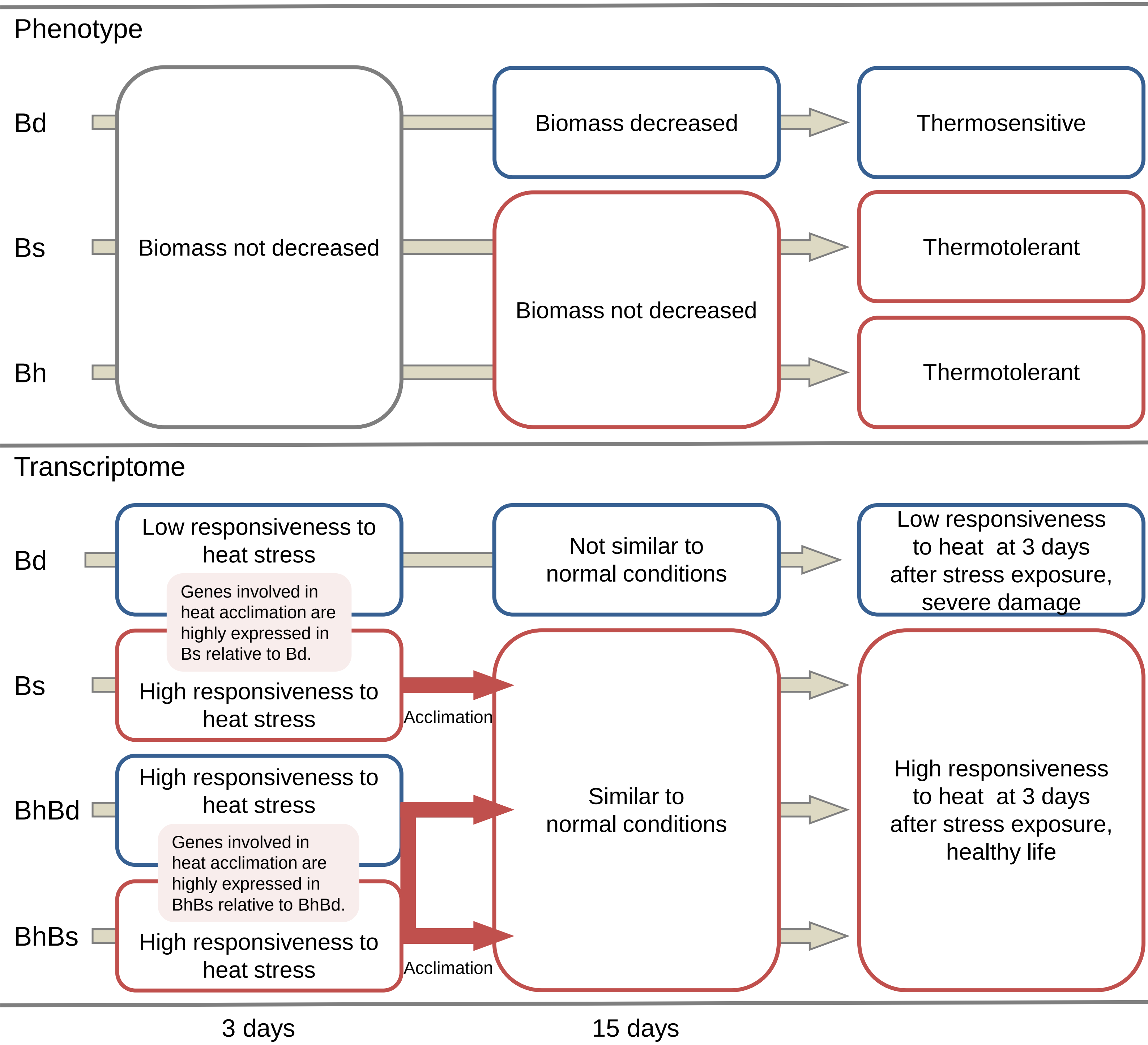

Additional file 1: Supplementary Figures

Click here to access/download
Supplementary Material
Additional file 1.pdf

Additional file 2: Supplementary Tables

Click here to access/download
Supplementary Material
Additional file 2.xlsx

Additional file 3: Homoeologous SNP dataset used in this study

Click here to access/download
Supplementary Material
Additional file 3.txt

Additional file 4: Original Perl script code used to construct the virtual <i>B. stacei</i> genome by replacing the nucleotides of the

Click here to access/download
Supplementary Material
Additional file 4.txt

Additional file 5: Original Perl script code used to classify the RNA-Seq reads of <i>B. hybridum</i> into the Bd-subgenome origin

Click here to access/download
Supplementary Material
Additional file 5.txt

Additional file 6: GO annotations of the <i>B. distachyon</i> genes used in this study

Click here to access/download
Supplementary Material
Additional file 6.txt
