# Supplementary material for: Homoeolog-specific activation of genes for heat acclimation in the allopolyploid grass Brachypodium hybridum
Source: Gigascience. 2018 Mar 8;7(4):giy020. doi: 10.1093/gigascience/giy020 (PMC5915950; doi:10.1093/gigascience/giy020)
Supplement: Additional Files [file giy020_supp.zip › Additional file 1.pdf]

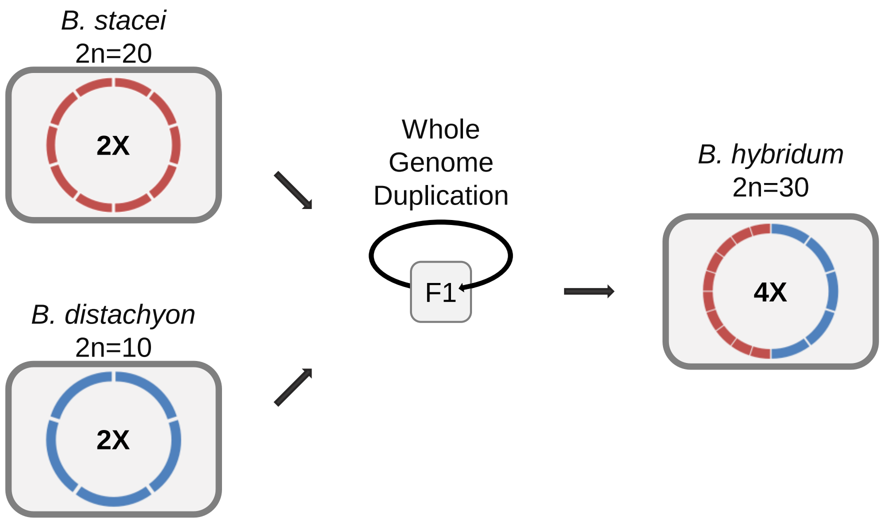

Figure S1. Phylogenetic relationships among the three *Brachypodium* species.

A

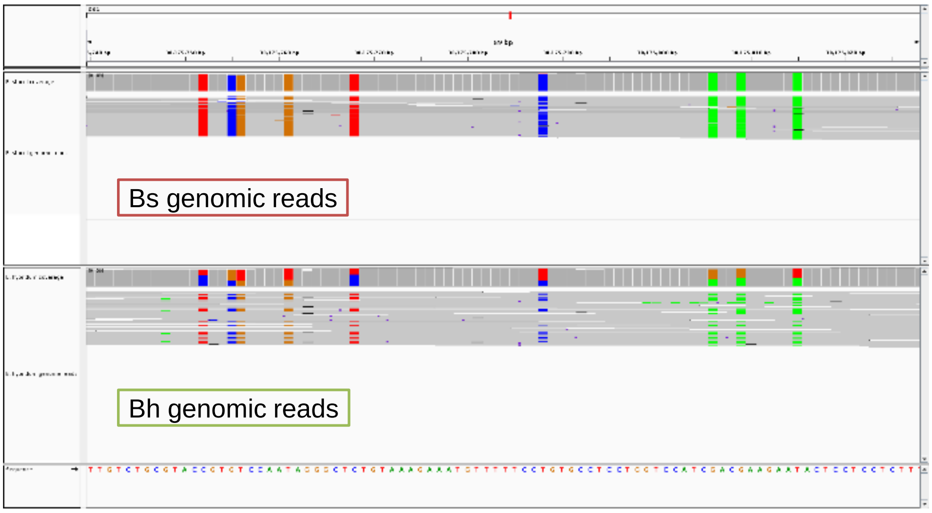

B

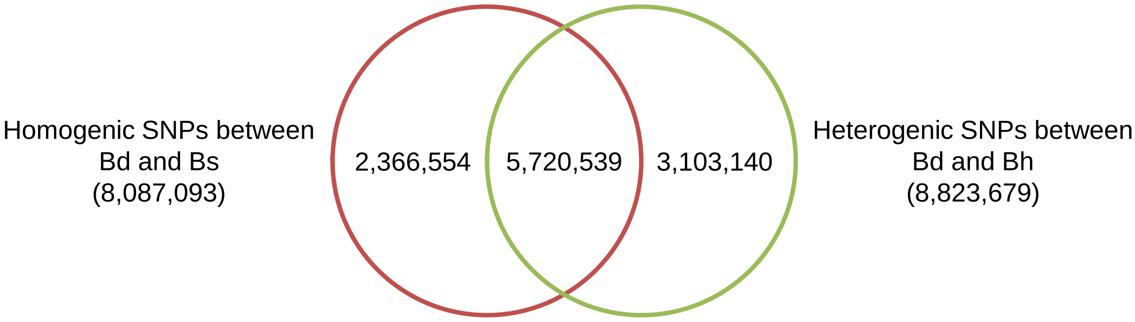

Figure S2. Results of homoeologous SNP identification. (**A**) The genomic reads of *B. stacei* and *B. hybridum* mapped to the reference genome sequence of Bd21. Different bases of the genomic reads against the reference genome are coloured. (**B**) Venn diagram of homogenic SNPs between *B. distachyon* and *B. stacei* (left) and heterogenic SNPs between *B. distachyon* and *B. hybridum* (right). The intersectional SNPs in the Venn diagram represent homoeologous SNPs in the three *Brachypodium* species used in this study. Bd, *B. distachyon*; Bs, *B. stacei*; Bh, *B. hybridum*.

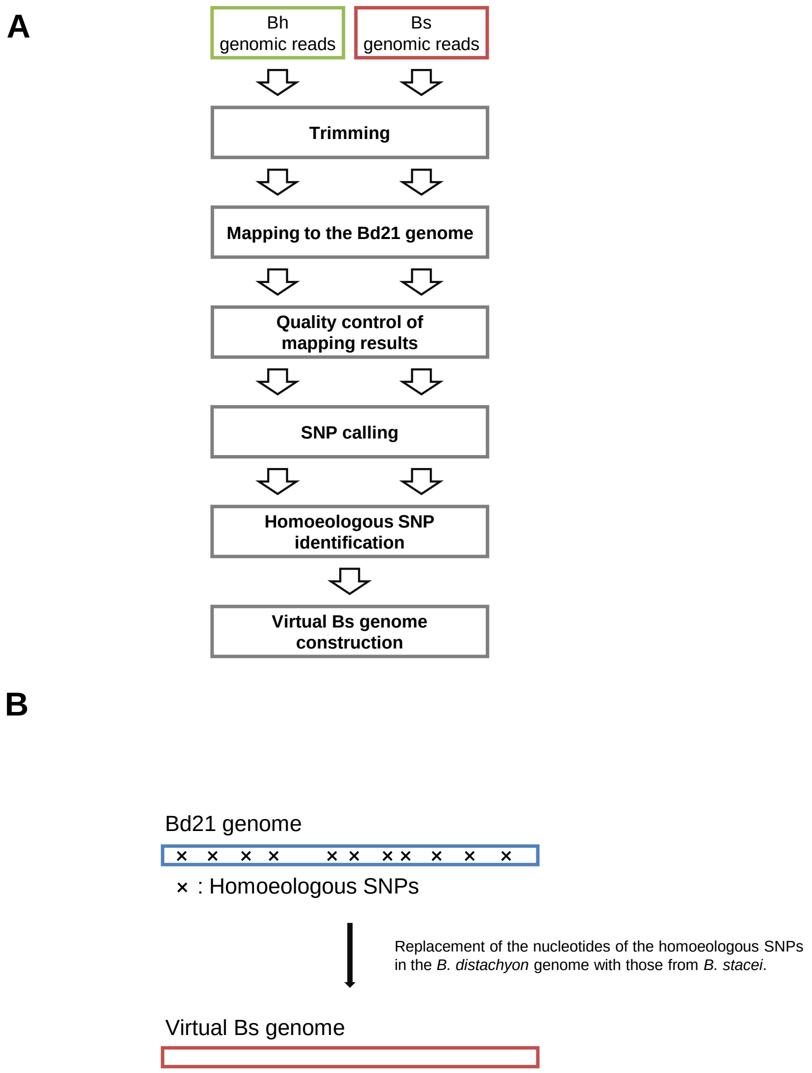

Figure S3. Overview of genomic sequence data analysis. (**A**) A flowchart depicting the construction of the virtual *B. stacei* genome. (**B**) Concrete method for the construction of the virtual *B. stacei* genome. Bd, *B. distachyon*; Bs, *B. stacei*; Bh, *B. hybridum*.

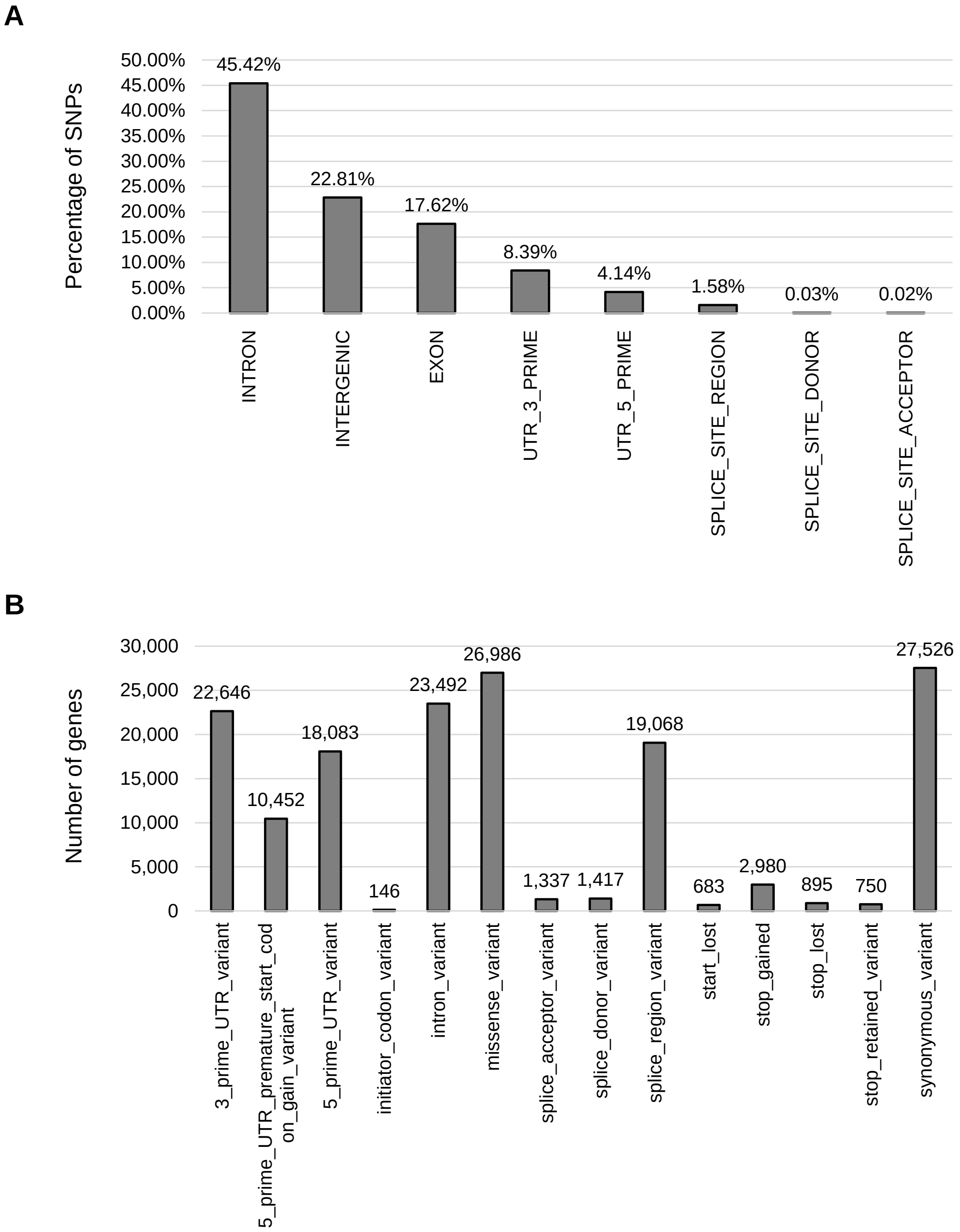

Figure S4. Location and effect of homoeologous SNPs. (**A**) Percentage of SNPs located in the Bd21 genomic region. (**B**) Number of genes possessing each variant effect.

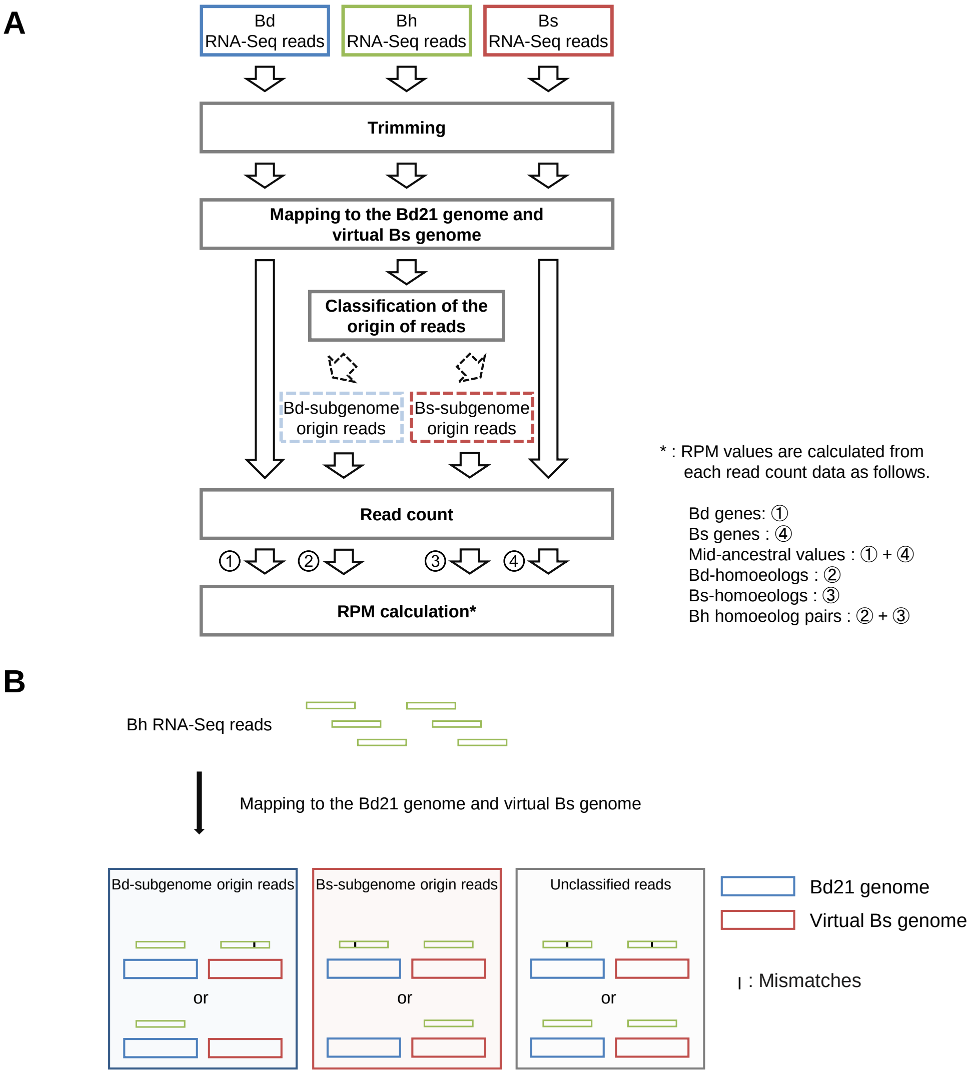

Figure S5. Overview of RNA-Seq data analysis. (**A**) A flowchart depicting RPM calculation. (**B**) Concrete method for the classification of the origin of the *B. hybridum* reads. Bd, *B. distachyon*; Bs, *B. stacei*; Bh, *B. hybridum*.

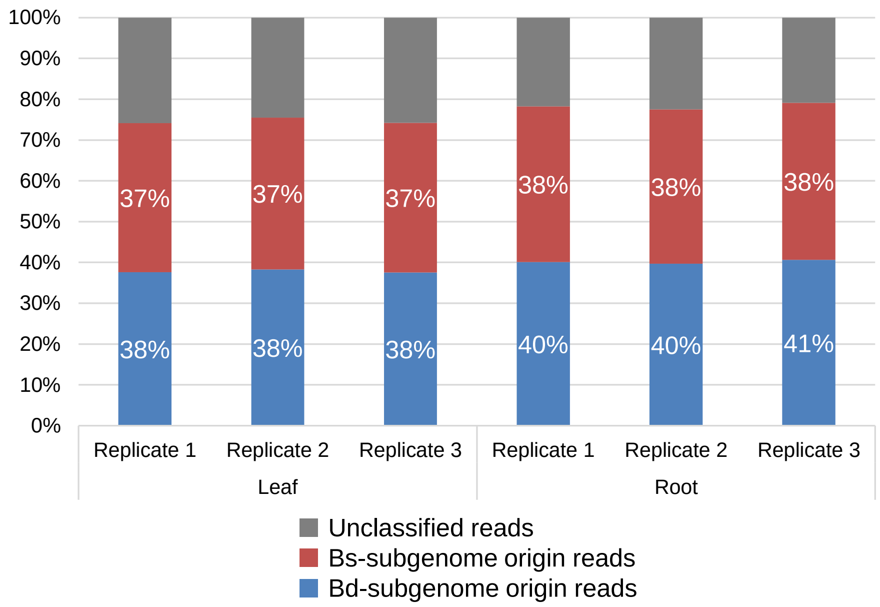

Figure S6. Classification of the read origin of the *B. hybridum* RNA reads from leaf and root tissues. The bars show the percentage of the Bd-subgenome origin reads (blue), Bs-subgenome origin reads (red), and unclassified reads (grey) of the *B. hybridum* RNA reads. Bd, *B. distachyon*; Bs, *B. stacei*.

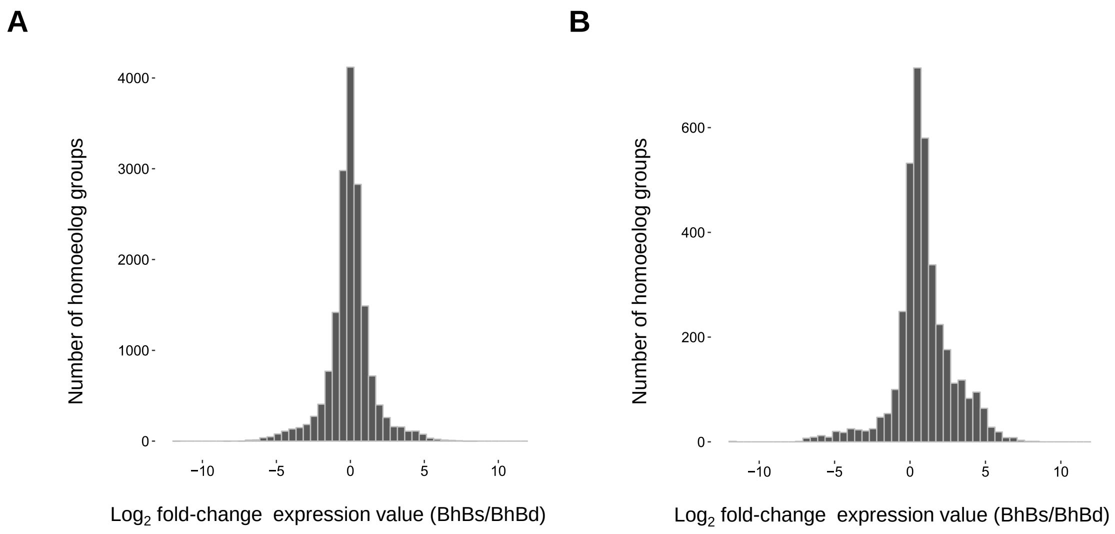

Figure S7. Log_2 fold-change distribution of homoeolog expression in *B. hybridum* under heat stress condition for 3 days. Histogram of log_2 fold-change in homoeolog expression (Bs-homoeologs/Bd-homoeologs) in all expressed homoeolog groups in *B. hybridum* (**A**) and those showing significantly higher expression in *B. hybridum* than in *B. distachyon* (**B**). BhBd, Bd-homoeologs in *B. hybridum*; BhBs, Bs-homoeologs in *B. hybridum*.

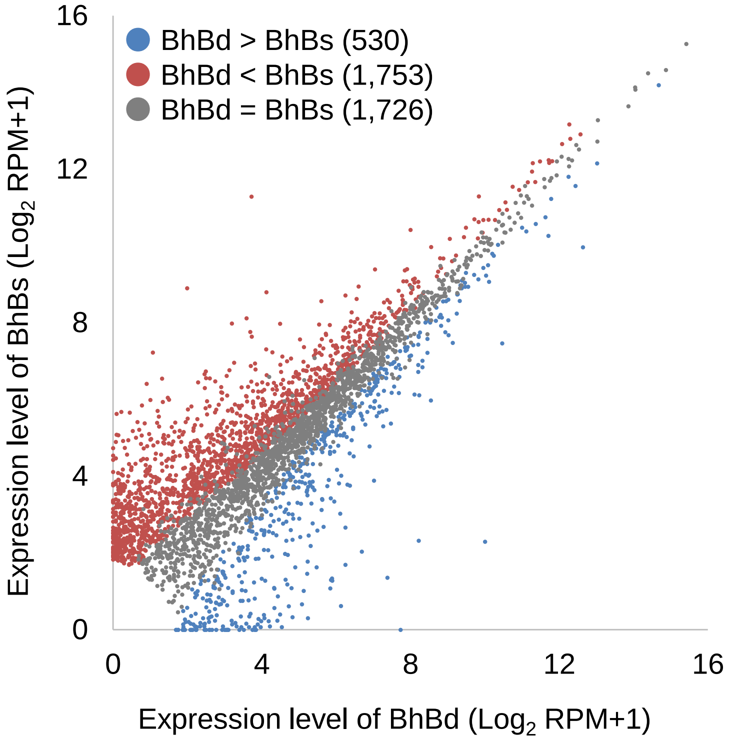

Figure S8. Gene expression profiles of the Bd- and Bs-homoeologs in homoeolog groups showing significantly higher expression in *B. hybridum* than in *B. distachyon* under heat stress condition for 15 days. Each dot represents the average expression value of three biological replicates. Blue dots represent genes showing significantly higher expression in Bd-homoeologs than in Bs-homoeologs and red dots represent genes showing significantly higher expression in Bs-homoeologs than in Bd-homoeologs (FDR ≤ 0.001). BhBd, Bd-homoeologs in *B. hybridum*; BhBs, Bs-homoeologs in *B. hybridum*.

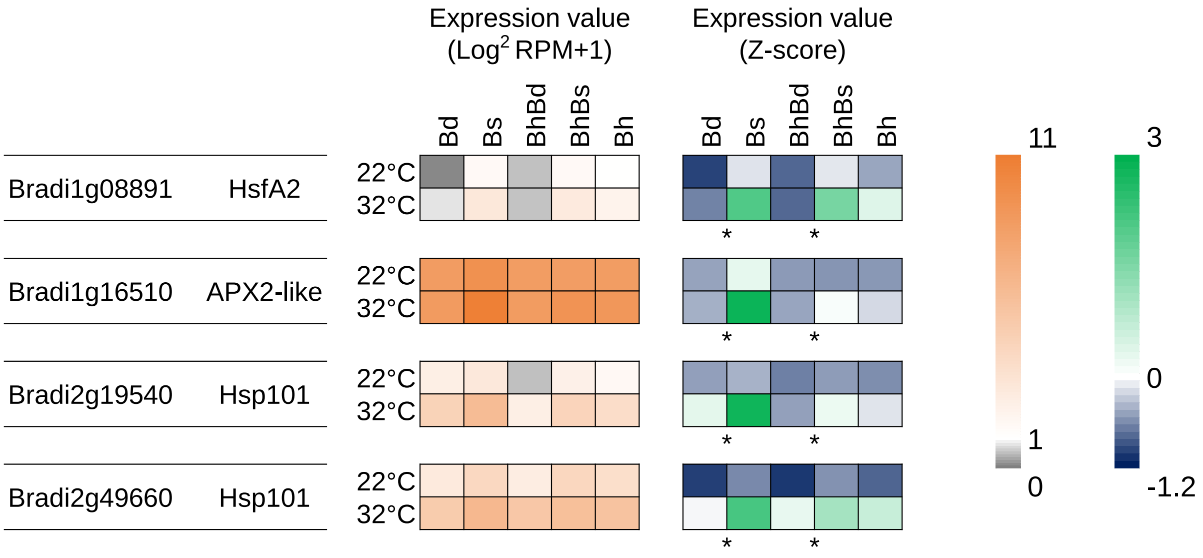

Figure S9. Gene expression profiles of the *Brachypodium HsfA2* and putative *HsfA2*-targeted genes at 3 days after heat stress exposure. The *HsfA2* is key regulator in heat response, and *APX2* and *Hsp101* are well-known *HsfA2*-targeted genes in *Arabidopsis* (see Charng et al. 2007; Schramm et al., 2006). The heat map shows gene expression profiles of *Brachypodium HsfA2* (*HsfA2a*; http://bioinfo.bti.cornell.edu/cgi-bin/itak/db_family_gene_list.cgi?acc=HSF&plant=15368), *APX2*-like (homologs of *Arabidopsis APX1* and rice *APX2*; Phytozome, Bdistachyon_314_v3.1.annotation_info.txt, http://genome.jgi.doe.gov/pages/dynamicOrganismDownload.jsf?organism=Bdistachyon) and *Hsp101* (Phytozome, Bdistachyon_314_v3.1.defline.txt, http://genome.jgi.doe.gov/pages/dynamicOrganismDownload.jsf?organism=Bdistachyon). Asterisks indicate genes and Bs-homoeologs showing significantly higher expression in *B. stacei* and *B. hybridum*, respectively, compared with their counterparts in *B. distachyon* under heat stress condition (FDR $\leq$ 0.001). Bd, *B. distachyon*; Bs, *B. stacei*; BhBd, Bd-homoeologs in *B. hybridum*; BhBs, Bs-homoeologs in *B. hybridum*; Bh, *B. hybridum*.

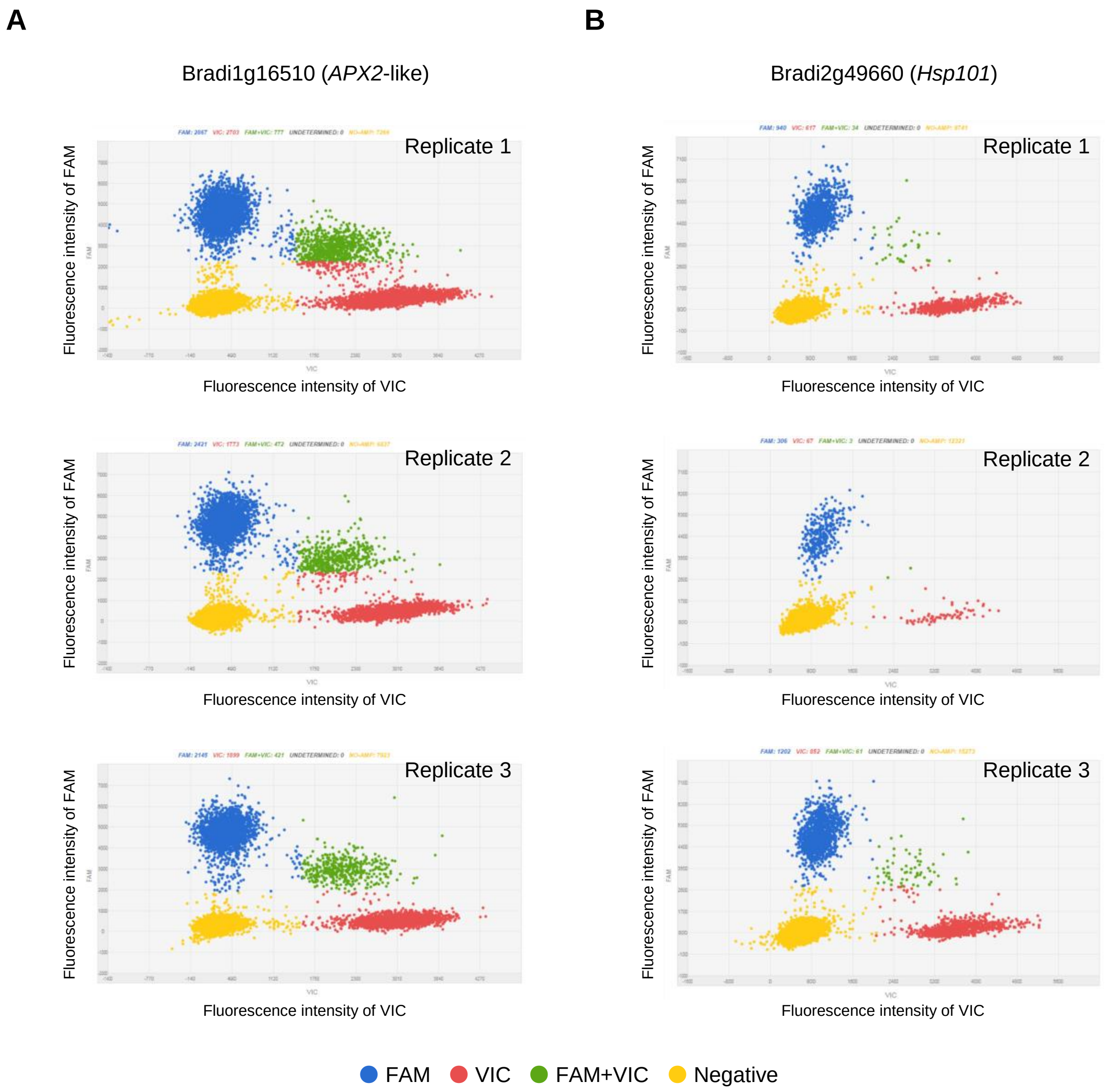

Figure S10. Quantitative detection of fluorescence of homoeologs in *B. hybridum*.

Fluorescence intensities of VIC (x-axis) and FAM (y-axis) for SNPs between two homoeolog groups in *B. hybridum* corresponding to the genes annotated in the Bd21 genome; Bradi1g16510 (*APX2*-like) (**A**) and Bradi2g49660 (*Hsp101*) (**B**). The SNPs were detected by the TaqMan SNP Genotyping Assay, and their fluorescence intensities were quantified by a digital PCR system. The data points in the plots are color-coded according to the following call types: FAM (homozygous of *B. stacei* type allele; blue), VIC (homozygous of *B. distachyon* type allele; red), FAM + VIC (hetelozygoous of *B. stacei* and *B. distachyon* type alleles; green) and Negative (not amplified; yellow).

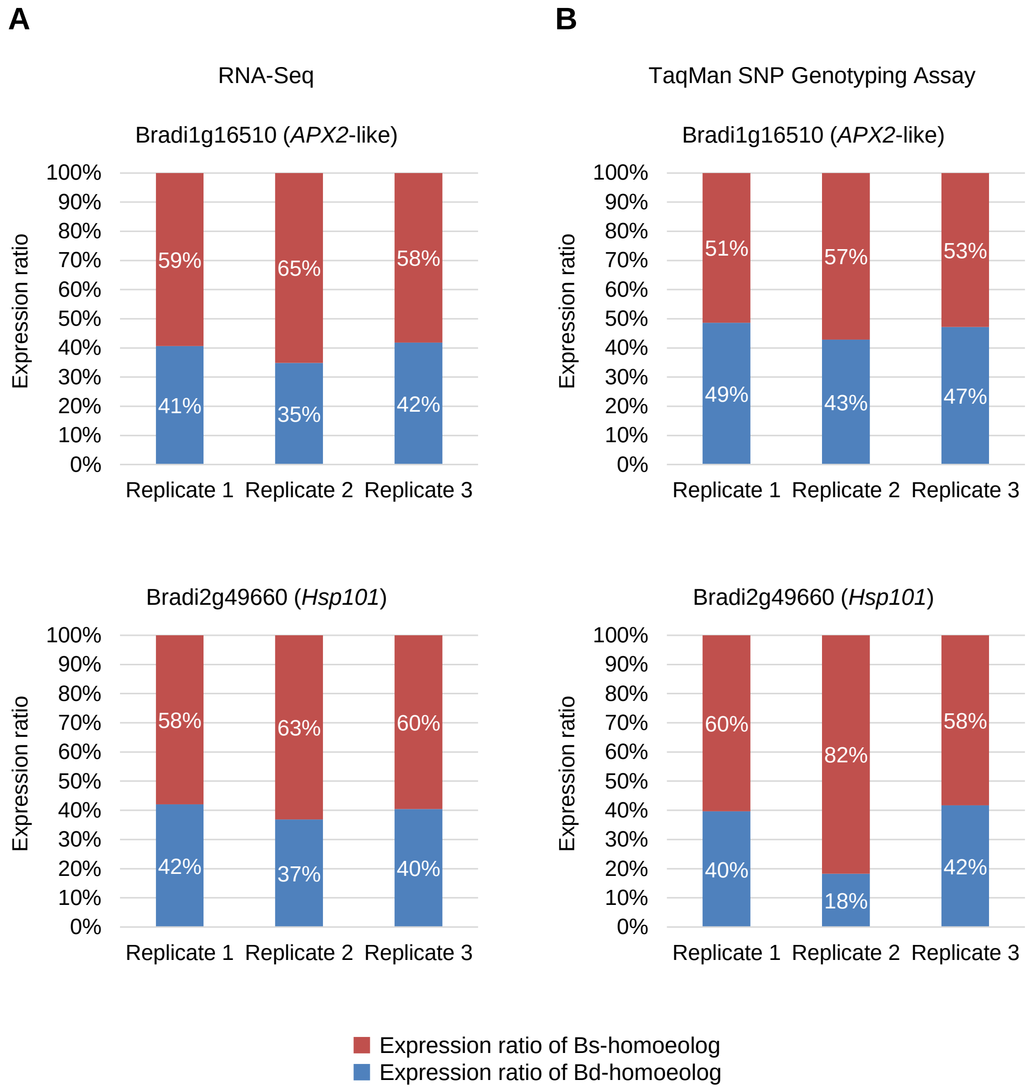

Figure S11. Expression ratios of Bd- and Bs-homoeologs based on RNA-Seq analysis and TaqMan SNP Genotyping Assay.

Expression ratios of Bd- and Bs-homoeologs in two homoeolog groups in *B. hybridum* corresponding to the genes annotated in the Bd21 genome, Bradi1g16510 (*APX2*-like) and Bradi2g49660 (*Hsp101*), based on RNA-Seq analysis (**A**) and TaqMan SNP Genotyping Assay (**B**) under heat stress conditions at 3 days after stress exposure.
