# Supplementary material for: Homoeolog-specific activation of genes for heat acclimation in the allopolyploid grass Brachypodium hybridum
Source: Gigascience. 2018 Mar 8;7(4):giy020. doi: 10.1093/gigascience/giy020 (PMC5915950; doi:10.1093/gigascience/giy020)
Supplement: Response_to_Reviewer_Comments_Original_Submission.pdf [file giy020_response_to_reviewer_comments_original_submission.pdf]

Author's Response To Reviewer Comments

December 15, 2017

Hans Zauner
Assistant Editor
GigaScience

Dear Dr. Hans Zauner,
We wish to express our appreciation to the editor and reviewers for their insightful comments on our paper. Their suggestions helped us significantly to improve the quality of the manuscript. To address the major concerns from both reviewers, we validated our results through a genomic analysis with B. stacei genome sequence dataset available at Phytozome and verified our results on homoeolog specific gene expressions through a quantification of homoeologous SNPs in cDNA amplicons by using TaqMan assay with a digital PCR. In addition to these major points, we have addressed all the comments from Reviewers #1 and #2 as detailed in a point-by-point list below. We hope the revised manuscript is acceptable for publication in *GigaScience*.

Sincerely,
Keiichi Mochida
Cellulose Production Research Team, Biomass Engineering Research Division, RIKEN Center for Sustainable Resource Science, 1-7-22 Suehiro-cho, Tsurumi-ku, Yokohama, Kanagawa 230-0045, Japan
Tel: +81-45-503-9111
Fax: +81-45-503-9182
E-mail: keiichi.mochida@riken.jp

Reviewer #1
Major comments
1. The major concern I want to raise is the confused use throughout the manuscript of the different terms regarding the evolution of duplicated genes. As explained in Grover et al. 2012 (and cited in the manuscript), it is essential to distinguish global gene expression from homoeo-specific expression. I would advise the authors to revise the manuscript accordingly and explain in details in the introduction the possible outcomes of allopolyploidy on gene expression. Briefly, one can look at global gene expression and detect events of additivity, genome dominance or transgressivity. Secondly, as the authors put a lot of efforts in detecting the homoeologues, one can compare their expression to a standard; either to a theoretical 50:50 ratio or from the ancestral gene patterns (homologous gene expression levels in both parental species). Thus, comparison of homoeo-specific expression in the allotetraploid can be balanced or unbalanced (skewed towards one or the other parent). As such when the authors disclaim "non-additive and non-parental gene expression patterns in B. hybridum" they could be much more precise when describing gene and homoeo-specific expression patterns. Thereafter, it would be interesting to investigate if specific pathways are enriched in the set of transgressively/over-expressed genes, for instance. I expect that this will reinforce considerably the impact of the

manuscript.

We appreciate your comment. As suggested, the possible outcomes of allopolyploidization on gene expression that were addressed in several previous reports and reviews, including Grover et al. (2012) and Yoo et al. (2014), were presented in the Introduction (Lines 80-104).
According to your suggestion, we identified the genes (homoeolog group) that show ELDs and homoeolog expression bias in the leaf and root tissues of B. hybridum. Specifically, we found that the number of homoeolog groups of ELD-Bs is almost twice that of ELD-Bd, suggesting that gene expression patterns of B. stacei are preferentially inherited by the B. hybridum transcriptome in its leaf and root tissues. These results are presented in newly added Figure 3 and elaborated in the Results section (Lines 233-240 and 254-266).
We were also interested in the enriched functions found in the set of transgressively/over expressed homoeolog groups. To identify enriched function in such genes, we performed GO enrichment analyses and found that GO terms such as primary metabolic processes, primary cellular processes, stress response, transport, localization, and translation were enriched in the transgressively/over expressed homoeolog groups. These results are presented in newly added Tables S5 and S6 and discussed in the Results section (Lines 240-246).

2. The ecology of the three species as well as their evolutionary history is understated in the manuscript. This would help discuss results. For instance, what is the expected time of divergence between both ancestral species and are the mapping results and number of SNPs identified in accordance with expectations? To what extend can the physiology and ecology of the species explain the enrichment of specific pathways.

To examine whether the divergence time between the two ancestral species and the number of SNPs identified in our study is as expected, we calculated the non-synonymous substitution rate (Ka) and synonymous substitution rate (Ks) between the homoeologs, and inferred the divergence time of the diploid ancestors of B. distachyon and B. stacei based on the Ks values. The divergence time was estimated to approximately 6.4 Mya, which falls within the divergence time of B. stacei (5.8–16.4 Mya) previously estimated by Catalan et al. (2012). We have added these results in the Results section (Lines 206-208).
Moreover, we identified 1,741 genes whose Ka/Ks values between homoeologs were greater than 1, supporting their accelerated evolution. We pursed the enrichment functions of those genes and found that genes involved in DNA mismatch repair with GO terms such as proliferating cell nuclear antigen complex and DNA polymerase processivity factor activity were enriched in those genes, which suggested that DNA damage tolerance may reflect selection and adaptive divergence between B. distachyon and B. stacei. These results were discussed in the Results section (Lines 208-214).

3. As previously said, the in silico assembly of the B. stacei genome is quite elegant. Nevertheless, I would appreciate some more details on the set of genes detected using the >5M homoeo-SNPs identified. Also, in the results section, authors are using the terms 'genes' and 'gene groups' that are not defined nor used previously.

We assessed the distribution of the SNPs within various genomic regions and applied the data to gain insights into genetic diversity of the two ancestral genomes. We found that 26,986 genes

(79% of the annotated genes in the Bd21 genome) contain nonsynonymous variations. Of those, 4,558 genes (13% of the annotated genes in the Bd21 genome) contained sever variations in the B. stacei genome: 683 genes contained no start codon, 2,980 genes had the stop codon, and 895 genes lacked the stop codon. These results were presented in Figure S4 and Table S4 and discussed in the Results (Lines 198-206).
Furthermore, we defined each of the homoeologs or homoeolog groups in B. hybridum as follows. Homoeolog groups: genes on Bd and Bs sub-genome in B. hybridum, Homoeologs: each gene in each of the subgenomes in B. hybridum.

Minor comments
Introduction
1. The introduction would take advantage of clear hypotheses regarding the evolution of global gene and homoeo-specific expression in B. hybridum.

We revised the introduction to explain the hypotheses regarding the evolution of transcriptome in B. hybridum as follows: "The broader growth area of B. hybridum compared to its ancestors led us to the hypothesis that its duplicated genes may have contributed to its adaptive and ecological fitness through gene expression changes following allotetraploidization. Specifically, in the B. hybridum transcriptome, each of the homoeologs may show particular expression patterns in response to abiotic stimuli, which are associated with the ancestral growth habitats and gene expression." (Lines 112-116).

Methods
2. It is not clear how the MAV was calculated.

We revised the sentences describing the calculation of MAV as follows: "As the MAV, which is the average of the expression values in both ancestors, the total read count data from the B. distachyon and B. stacei reads were used" (Lines 427-428).

3. Could the authors provide more details on the trimming step. For both DNA and RNA-Seq, it is also unclear how the coverage was calculated and what were the parameters used to detect the SNPs (minimum read coverage and number of reads).

We added additional information about the trimming and the SNP calling steps (Lines 380-381, 394-396, and 416-417).

4. Line 307-308: What were the identity thresholds used to divide the three groups?

We described the identity thresholds used to divide the RNA-Seq reads from B. hybridum into the three groups as follows: "The expression levels of the homoeologs in B. hybridum were quantified by classifying the RNA-Seq reads into the following three groups based on the number of mismatches between read and both genomes using original Perl script (Additional file 5): Bd-subgenome origin reads, Bs-genome origin reads, and unclassified reads (Additional file 1; Figure S5B)." (Lines 421-424).

5. Line 318: Differentially and not deferentially

We revised the term.

6. DESeq2: Did the authors perform a filtration step of the least expressed data?

For the filtration step, we used genes that showed significant expression with the RPM values more than 1 (Lines 431-432 and 451).

7. GO enrichment: What was the reference set of genes used in the GO term enrichment analysis?

We added sentences that explain the reference set of genes used in the GO term enrichment analysis in the Methods section (Lines 465-466).

8. Line 308: Genome instead of gnome.

We revised the typographical error.

Results

9. Lines 172-188: Additionally to the proportions of genes (or homoeologues) differentially expressed, would be useful to add the number of genes altered.

We added the data to explain the proportions of homoeolog groups showing non-additive and non-ancestral expressions in B. hybridum in Lines 230, 234, 237, and 241. We also listed the numbers and proportions of genes and homoeologs differentially expressed in the revised Figure 3.

10. Lines 201 to 209: Sentences are not clear.

We revised the sentences for clarity as follows: "We then dissected the expression patterns of the 3,725 homoeolog groups from B. hybridum into Bd- and Bs-homoeologs, and found that 2,088 homoeolog groups (56% of the 3,725 homoeolog groups) were preferentially expressed by the Bs-homoeologs (Figure 4B). The distribution of fold-change expression values between Bd- and Bs-homoeologs in all expressed homoeolog groups in B. hybridum under heat stress for 3 days indicated that most of the homoeologs were evenly expressed by both subgenomes (Additional file 1: Figure S7A), whereas the 3,725 homoeolog groups showed abundant expression in the Bs-homoeologs (Additional file 1: Figure S7B). Additionally, of the 2,088 Bs-homoeologs, 1,791 genes were shared with the 5,649 genes that showed higher expression in B. stacei than in B. distachyon; these genes included those specifically involved in metabolic processes as well as cellular response to stress and damage stimulus (Figures 4C and D)." (Lines 279-289).

11. Figure 6 is unclear, needs more explanations in the caption and the manuscript.

We revised the part in the manuscript related to Figure 6 (Lines 360-363).

Reviewer #2
Major comments
1. One of the main observations in the study is that "B. distachyon was transcriptionally insensitive to heat after 3 days of exposure to heat stress…" (pg.11, ln. 190), while B. stacei and B. hybridum are responsive. And, this lead to the conclusion that B. stacei and B. hybridium may be better acclimated to heat stress, through the early activation of heat stress responsive genes. This conclusion was primarily based on a Pearson correlation analyses of the global expression changes among the three species under stressed vs. unstressed conditions (Fig. 4A). I am skeptical about this conclusion---given that other published studies show that B. distachyon transcriptome is highly responsive to heat stress, as early as 10-24 h, and >1000 genes are differentially expressed (Priest et al., 2014, PLoS One, 9(1): e87499). It would be highly implausible that there would be no quantitative or qualitative differences in gene expression profiles in B. distachyon, even after 3 days of heat stress. And, if there are some changes in gene expression, it cannot be concluded that B. distachyon transcriptome is 'transcriptionally insensitive".

We appreciate the comments and agree with the suggestions. We revised the sentences as follows: "B. distachyon transcriptomes were not significantly different between normal (22°C) and heat stress (32°C) conditions after 3 days of exposure to heat stress, whereas the B. hybridum transcriptome was noticeably altered under the heat stress as compared to the normal condition; this might reflect physiological traits inherited from B. stacei. When gene expression patterns of B. distachyon, B. stacei, and B. hybridum, as well as the Bd- and Bs-homoeologs of B. hybridum after 3 days of exposure to heat stress were compared, we found that the transcriptomes of B. stacei and B. hybridum showed lower correlations between normal and heat stress conditions than those of B. distachyon (Pearson's correlation coefficient (PCC) 0.68–0.75, 0.87, and 0.92–0.95 in B. stacei, B. hybridum, and B. distachyon, respectively; Figure 4A), suggesting that B. stacei and B. hybridum respond more strongly to the heat stress than B. distachyon." (Lines 269-277).

2-1. B. stacei and B. distachyon genomes are publicly available at Phytozome (available since 2015). It would have been easier and appropriate to compare and identify homeologs of B. hybridium by mapping the reads to the parental genomes, rather re-sequencing and constructing a virtual B. stacei genome based on SNPs.

We are aware that B. stacei genome sequence is available at Phytozome. However, according to the policy issued by JGI, we are not allowed to publish any articles containing the analyses of genes or genomic data on a whole genome or chromosome scale using the B. stacei genome sequence prior to publication by JGI and/or its collaborators of a comprehensive genome analysis (https://phytozome.jgi.doe.gov/pz/portal.html#). Therefore, we identified genome-wide homoeologous SNPs using our own sequencing data of B. stacei and B. hybridum and constructed a virtual B. stacei genome. To verify the results of our homoeolog specific gene expression analysis, we calculated the Pearson's correlation coefficient between the expression values of each homoeolog calculated by using our virtual B. stacei genome and the B. stacei genome available at Phytozome. The Pearson's correlation coefficient was more than 0.97, suggesting that our homoeolog specific gene expression analysis based on our virtual B. stacei

genome was comparable to that of the B. stacei genome.

2-2. Besides, for the SNP calling, the authors utilized SAMtools. However, for polyploid genomes, SAMtools is not appropriate, as it is designed for diploid species. The authors should have used tools like GATK, FreeBayes that are superior and better for polyploid SNP calling. Further, the methods and parameters used for SNP calling are not provided in sufficient detail (i.e., coverage, depth etc.). To corroborate these analyses, suggest authors to compare and validate their SNPs/homeologous transcript models with the existing draft B. stacei genome.

We had considered using several tools for SNP calling, including GATK and FreeBayes. However, although B. hybridum is a polyploid species, it is also a self-fertilizing species (Catalán et al., 2012 and 2016) and its homoeologous genomes are homozygous. Therefore, we decided to use samtools and VarScan programs to call SNPs between mapped reads of B. stacei and B. hybridum and the reference genome sequence of the B. distachyon Bd21.

3. It's not clear why the authors did not provide details of interesting differentially regulated homeologous genes, and their respective expression fold-changes. They did mention four genes in the supplemental Table S8, but even those are described without any mention of the fold-change cutoffs. So, what is the fold-change cutoff used in the entire study for the various comparisons. Also, the authors use GO term descriptions to predict overall pathways enriched among the differentially expressed homeologs. The GO terms are good starting points, but can be broad and the enriched categories need to be filtered to remove redundant/overlapping terms.

Following your suggestions, we mentioned the genes in Table S8 (Table S13 in the revised manuscript) as follows “The comprehensive list of Bs-homoeolog and B. stacei genes with significantly higher expression than the B. distachyon genes after 3-day exposure to 32°C included genes involved in the regulation of acclimation to heat in plants, such as heat shock transcription factor (Hsf), heat shock protein (Hsp), and DNAJ [52-55] (1,791 genes in Figure 4C, Additional file 2: Table S13).”(Lines 355-358). Moreover, we also noted that we used FDR as a threshold to identify DEGs in the Methods section (Line 451). Finally, we summarized the enriched GO terms using REVIGO (http://revigo.irb.hr/) and added this information in the Methods section (Lines 466-467).

4. The narrative corresponding to the results presented in Fig. 3 need to be elaborated. In this section, the authors write that "the homoeologous transcripts were almost evenly expressed between the B. distachyon and B. stacei genomes in the B. hybridum transcriptome of the leaf and root tissues under normal conditions" (Pg.10-11, ln 177-179), but at the same time conclude that "~26% and ~38% of the homoeologous gene pairs in B. hybridum changed its expression pattern towards non-additive expression and non-parental expression" (Pg.11, ln 181). It's not clear to me what can be concluded from these statements, and how the specific analysis pertaining to Fig. 3 was performed.
Authors need to define and describe the methodology for estimation of mid-ancestral values (MAVs), non-additive, non-parental expression terms so that non-evolutionary biologists can also understand this section. Besides this analysis was performed only using unstressed tissues. It would have been more
informative to analyze how these different polyploid gene expression patterns (non-additive or

non-parental) change under heat stress.

Following the comment pertaining to the proportion of genes that show non-ancestral expression Figure 3 and Figure S6, we rephrased the related text in the revised manuscript (Lines 249-254 and 263-266). To address the comments about the method used to estimate MAV, we revised the text that describes the method for MAV calculation as follows: “As the MAV, which is the average of the expression values in both ancestors, the total read count data from the B. distachyon and B. stacei reads were used” (Lines 427-428).
Moreover, to facilitate the understanding of gene expression patterns in allopolyploid species such as non-additive expression, we added the possible outcomes of transcriptome in allopolyploidy on gene expression in the Introduction section (Lines 80-104).
As per the comments on our identification of differentially expressed homoeologs in response to heat stress, we identified genes showing non-additive expression under heat stress conditions and listed enriched functions based on their GO annotations. We found that genes involved in biological functions such as response to stimulus and abiotic stimulus were enriched in those genes, which suggests that increased expression divergence of genes related to these functions enhanced their ability to respond to environmental change and adapt to ecological niches. These results were presented in newly added Tables S11 and S12 and discussed in the Discussion (Lines 333-337).

5. There was no validation of the RNA-seq data by alternate approaches such as RT-qPCR. This is very important QC for any transcriptome-level study. This is even more relevant for this study that relates to homeolog reconstruction and expression differences of homeologous genes. I would suggest validating the expression differences of the homeologs, that can corroborate both the genome-level and RNA-seq analysis.

As suggested, we validated the expression ratios of homoeologs found in B. hybridum by using the TaqMan SNP Genotyping Assay in combination with a digital PCR system. Specifically, we assessed the quantitative distribution of the decisive SNPs in the two homoeolog groups corresponding to the genes annotated in the Bd21 genome; Bradi1g16510 (APX2-like) and Bradi2g49660 (Hsp101). We found that the proportions of the SNPs in the cDNA amplicons from the B. hybridum RNAs sampled after 3 days of exposure to 32°C were comparable to their expression bias observed in the RNA-seq analysis. These results were presented in a newly added Figure S10 and S11and discussed in the Results (Lines 313-322).
