# Supplementary material for: Homoeolog-specific activation of genes for heat acclimation in the allopolyploid grass Brachypodium hybridum
Source: Gigascience. 2018 Mar 8;7(4):giy020. doi: 10.1093/gigascience/giy020 (PMC5915950; doi:10.1093/gigascience/giy020)
Supplement: Response_to_Reviewer_Comments_Revision_1.pdf [file giy020_response_to_reviewer_comments_revision_1.pdf]

Author's Response To Reviewer Comments

February 9, 2018

Dear Dr. Hans Zauner

We thank you so much for handling and careful reading our manuscript as well as for your valuable comments and suggestions.

We addressed the points that you suggested.

1) GigaDB citation:
We included a citation to our upcoming GigaDB dataset (including the dummy DOI link) to the reference list, and cited this in the data availability section (Lines 464 and 707-709).

2) Please read over your manuscript carefully again to correct typos and minor grammatical issues (a few here and there).
We read our manuscript again and made a few corrections.

3) I wonder whether the title needs a minor clarification?
According to your suggestion, we changed our title to "Homoeolog-specific activation of heat acclimation mechanisms in the allopolyploid grass *Brachypodium hybridum*" to clarify the meaning of "Activation".

4) Protocols.io:
We are able to provide our methods used in this study. I would appreciate it if you could send us the spreadsheet. We'll fill out and send the spreadsheet.

5) URLs:
We moved all URLs within the text of the manuscript to the reference list and provided their access dates.

We hope that the revised manuscript meets the quality of *GigaSciense*.

Sincerely yours,

Keiichi Mochida
