# Supplementary material for: Homoeolog-specific activation of genes for heat acclimation in the allopolyploid grass Brachypodium hybridum
Source: Gigascience. 2018 Mar 8;7(4):giy020. doi: 10.1093/gigascience/giy020 (PMC5915950; doi:10.1093/gigascience/giy020)
Supplement: Reviewer_1_Report_(Original_Submission) -- Julie Ferreira de Carvalho — 07 Sep 2017 Reviewed [file giy020_reviewer_1_report_(original_submission).pdf]

Reviewer Report

Title: Homoeolog-specific activation of genes for heat acclimation in the allopolyploid grass *Brachypodium hybridum*

Version: Original Submission **Date:** 07 Sep 2017

Reviewer name: Julie Ferreira de Carvalho

Reviewer Comments to Author:

In this study, Takahagi et al. investigate gene expression evolution in an allotetraploid species after heat exposure. The Brachypodium is a perfect model to elucidate the faith of homoeologous genes after interspecific merger. The contrasted ecology of the parental species makes it all the more interesting to assess sub-genome evolution in the allotetraploid B. hybridum. I particularly praise the clever low coverage sequencing of the species B. stacei to overcome the lack of a reference genome for this parental species. I have nonetheless, substantial comments notably on the conceptual framework of gene expression evolution in a polyploid species.
Major comments
1. The major concern I want to raise is the confused use throughout the manuscript of the different terms regarding the evolution of duplicated genes. As explained in Grover et al. 2012 (and cited in the manuscript), it is essential to distinguish global gene expression from homoeo-specific expression. I would advise the authors to revise the manuscript accordingly and explain in details in the introduction the possible outcomes of allopolyploidy on gene expression. Briefly, one can look at global gene expression and detect events of additivity, genome dominance or transgressivity. Secondly, as the authors put a lot of efforts in detecting the homoeologues, one can compare their expression to a standard; either to a theoretical 50:50 ratio or from the ancestral gene patterns (homologous gene expression levels in both parental species). Thus, comparison of homoeo-specific expression in the allotetraploid can be balanced or unbalanced (skewed towards one or the other parent). As such when the authors disclaim "non-additive and non-parental gene expression patterns in B. hybridum" they could be much more precise when describing gene and homoeo-specific expression patterns. Thereafter, it would be interesting to investigate if specific pathways are enriched in the set of transgressively/over- expressed genes, for instance. I expect that this will reinforce considerably the impact of the manuscript.
2. The ecology of the three species as well as their evolutionary history is understated in the manuscript. This would help discuss results. For instance, what is the expected time of divergence between both ancestral species and are the mapping results and number of SNPs identified in accordance with expectations? To what extend can the physiology and ecology of the species explain the enrichment of specific pathways.
3. As previously said, the in silico assembly of the B. stacei genome is quite elegant. Nevertheless, I would appreciate some more details on the set of genes detected using the >5M homoeo-SNPs identified. Also, in the results section, authors are using the terms 'genes' and 'gene groups' that are not defined nor used previously.

Minor comments
Introduction
1. The introduction would take advantage of clear hypotheses regarding the evolution of global gene and homoeo-specific expression in B. hybridum.
Methods
2. It is not clear how the MAV was calculated.
3. Could the authors provide more details on the trimming step. For both DNA and RNA-Seq, it is also unclear how the coverage was calculated and what were the parameters used to detect the SNPs (minimum read coverage and number of reads).
4. Line 307-308: What were the identity thresholds used to divide the three groups?
5. Line 318: Differentially and not deferentially
6. DESeq2: Did the authors perform a filtration step of the least expressed data?
7. GO enrichment: What was the reference set of genes used in the GO term enrichment analysis?
8. Line 308: Genome instead of gnome.
Results
9. Lines 172-188: Additionally to the proportions of genes (or homoeologues) differentially expressed, would be useful to add the number of genes altered.
10. Lines 201 to 209: Sentences are not clear.
11. Figure 6 is unclear, needs more explanations in the caption and the manuscript.

Methods

Are the methods appropriate to the aims of the study, are they well described, and are necessary controls included? Yes

Conclusions

Are the conclusions adequately supported by the data shown? No

Reporting Standards

Does the manuscript adhere to the journal's guidelines on minimum standards of reporting? No

Statistics

Are you able to assess all statistics in the manuscript, including the appropriateness of statistical tests used? Yes, and I have assessed the statistics in my report.

Quality of Written English

Please indicate the quality of language in the manuscript: Acceptable

Declaration of Competing Interests

Please complete a declaration of competing interests, considering the following questions:

- Have you in the past five years received reimbursements, fees, funding, or salary from an organisation that may in any way gain or lose financially from the publication of this manuscript, either now or in the future?
- Do you hold any stocks or shares in an organisation that may in any way gain or lose financially from the publication of this manuscript, either now or in the future?
- Do you hold or are you currently applying for any patents relating to the content of the manuscript?
- Have you received reimbursements, fees, funding, or salary from an organization that holds or has applied for patents relating to the content of the manuscript?
- Do you have any other financial competing interests?
- Do you have any non-financial competing interests in relation to this paper?

If you can answer no to all of the above, write 'I declare that I have no competing interests' below. If your reply is yes to any, please give details below.

I declare that I have no competing interests.

I agree to the open peer review policy of the journal. I understand that my name will be included on my report to the authors and, if the manuscript is accepted for publication, my named report including any attachments I upload will be posted on the website along with the authors' responses. I agree for my report to be made available under an Open Access Creative Commons CC-BY license (http://creativecommons.org/licenses/by/4.0/). I understand that any comments which I do not wish to be included in my named report can be included as confidential comments to the editors, which will not be published.

I agree to the open peer review policy of the journal

To further support our reviewers, we have joined with Publons, where you can gain additional credit to further highlight your hard work (see: https://publons.com/journal/530/gigascience). On publication of this paper, your review will be automatically added to Publons, you can then choose whether or not to claim your Publons credit. I understand this statement.

Yes
