# Supplementary material for: Homoeolog-specific activation of genes for heat acclimation in the allopolyploid grass Brachypodium hybridum
Source: Gigascience. 2018 Mar 8;7(4):giy020. doi: 10.1093/gigascience/giy020 (PMC5915950; doi:10.1093/gigascience/giy020)
Supplement: Reviewer_2_Report_(Original_Submission) -- Kranthi Mandadi — 14 Sep 2017 Reviewed [file giy020_reviewer_2_report_(original_submission).pdf]

Reviewer Report

Title: Homoeolog-specific activation of genes for heat acclimation in the allopolyploid grass *Brachypodium hybridum*

Version: Original Submission **Date:** 14 Sep 2017

Reviewer name: Kranthi Mandadi

Reviewer Comments to Author:

Reviewer Comments- GIGA-D-17-00181

This study describes genome-wide transcriptomic changes and differential gene expression patterns during heat acclimation in the allopolyploid grass Brachypodium hybridum. Specifically, the authors analyzed the impact of heat stress on homeologous gene expression of B. hybridum by comparing the expression changes of B. hybridum and its ancestral species, B. distachyon and B. stacei. The proposed work has a good rationale, and follows recent advances in utilizing Brachypodium as a model grass to understand grass evolutionary biology. Overall, the manuscript was well-written and has novel elements with regards to homeologous transcriptome ananlysis. However, there are some critical aspects that need to be addressed related to the analysis, interpretation, and presentation of the results.
* One of the main observations in the study is that "B. distachyon was transcriptionally insensitive to heat after 3 days of exposure to heat stress…" (pg.11, ln. 190), while B. stacei and B. hybridum are responsive. And, this lead to the conclusion that B. stacei and B. hybridium may be better acclimated to heat stress, through the early activation of heat stress responsive genes. This conclusion was primarily based on a Pearson correlation analyses of the global expression changes among the three species under stressed vs. unstressed conditions (Fig. 4A). I am skeptical about this conclusion---given that other published studies show that B. distachyon transcriptome is highly responsive to heat stress, as early as 10-24 h, and >1000 genes are differentially expressed (Priest et al., 2014, PLoS One, 9(1): e87499). It would be highly implausible that there would be no quantitative or qualitative differences in gene expression profiles in B. distachyon, even after 3 days of heat stress. And, if there are some changes in gene expression, it cannot be concluded that B. distachyon transcriptome is 'transcriptionally insensitive".
* B. stacei and B. distachyon genomes are publicly available at Phytozome (available since 2015). It would have been easier and appropriate to compare and identify homeologs of B. hybridium by mapping the reads to the parental genomes, rather re-sequencing and constructing a virtual B. stacei genome based on SNPs. Besides, for the SNP calling, the authors utilized SAMtools. However, for polyploid genomes, SAMtools is not appropriate, as it is designed for diploid species. The authors should have used tools like GATK, FreeBayes that are superior and better for polyploid SNP calling. Further, the methods and parameters used for SNP calling are not provided in sufficient detail (i.e., coverage, depth etc.). To corroborate these analyses, suggest authors to compare and validate their SNPs/homeologous transcript models with the existing draft B. stacei genome.
* It's not clear why the authors did not provide details of interesting differentially regulated

homeologous genes, and their respective expression fold-changes. They did mention four genes in the supplemental Table S8, but even those are described without any mention of the fold-change cutoffs. So, what is the fold-change cutoff used in the entire study for the various comparisons. Also, the authors use GO term descriptions to predict overall pathways enriched among the differentially expressed homeologs. The GO terms are good starting points, but can be broad and the enriched categories need to be filtered to remove redundant/overlapping terms.
* The narrative corresponding to the results presented in Fig. 3 need to be elaborated. In this section, the authors write that "the homoeologous transcripts were almost evenly expressed between the B. distachyon and B. stacei genomes in the B. hybridum transcriptome of the leaf and root tissues under normal conditions" (Pg.10-11, ln 177-179), but at the same time conclude that "~26% and ~38% of the homoeologous gene pairs in B. hybridum changed its expression pattern towards non-additive expression and non-parental expression" (Pg.11, ln 181). It's not clear to me what can be concluded from these statements, and how the specific analysis pertaining to Fig. 3 was performed. Authors need to define and describe the methodology for estimation of mid-ancestral values (MAVs), non-additive, non-parental expression terms so that non-evolutionary biologists can also understand this section. Besides this analysis was performed only using unstressed tissues. It would have been more informative to analyze how these different polyploid gene expression patterns (non-additive or non-parental) change under heat stress.
* There was no validation of the RNA-seq data by alternate approaches such as RT-qPCR. This is very important QC for any transcriptome-level study. This is even more relevant for this study that relates to homeolog reconstruction and expression differences of homeologous genes. I would suggest validating the expression differences of the homeologs, that can corroborate both the genome-level and RNA-seq analysis.

Methods

Are the methods appropriate to the aims of the study, are they well described, and are necessary controls included? No

Conclusions

Are the conclusions adequately supported by the data shown? No

Reporting Standards

Does the manuscript adhere to the journal's guidelines on minimum standards of reporting? Yes

Statistics

Are you able to assess all statistics in the manuscript, including the appropriateness of statistical tests used? Yes, and I have assessed the statistics in my report.

Quality of Written English

Please indicate the quality of language in the manuscript: Acceptable

Declaration of Competing Interests

Please complete a declaration of competing interests, considering the following questions:

- Have you in the past five years received reimbursements, fees, funding, or salary from an organisation that may in any way gain or lose financially from the publication of this manuscript, either now or in the future?
- Do you hold any stocks or shares in an organisation that may in any way gain or lose financially from the publication of this manuscript, either now or in the future?
- Do you hold or are you currently applying for any patents relating to the content of the manuscript?
- Have you received reimbursements, fees, funding, or salary from an organization that holds or has applied for patents relating to the content of the manuscript?
- Do you have any other financial competing interests?
- Do you have any non-financial competing interests in relation to this paper?

If you can answer no to all of the above, write 'I declare that I have no competing interests' below. If your reply is yes to any, please give details below.

I declare that I have no competing interests

I agree to the open peer review policy of the journal. I understand that my name will be included on my report to the authors and, if the manuscript is accepted for publication, my named report including any attachments I upload will be posted on the website along with the authors' responses. I agree for my report to be made available under an Open Access Creative Commons CC-BY license (http://creativecommons.org/licenses/by/4.0/). I understand that any comments which I do not wish to be included in my named report can be included as confidential comments to the editors, which will not be published.

I agree to the open peer review policy of the journal

To further support our reviewers, we have joined with Publons, where you can gain additional credit to further highlight your hard work (see: https://publons.com/journal/530/gigascience). On publication of this paper, your review will be automatically added to Publons, you can then choose whether or not to claim your Publons credit. I understand this statement.

Yes
